# Organosolv Pretreatment of Cocoa Pod Husks: Isolation, Analysis, and Use of Lignin from an Abundant Waste Product

**DOI:** 10.1021/acssuschemeng.2c03670

**Published:** 2023-09-21

**Authors:** Daniel J. Davidson, Fei Lu, Laura Faas, Daniel M. Dawson, Geoffrey P. Warren, Isabella Panovic, James R. D. Montgomery, Xiaoyan Ma, Boris G. Bosilkov, Alexandra M. Z. Slawin, Tomas Lebl, Afroditi Chatzifragkou, Steve Robinson, Sharon E. Ashbrook, Liz J. Shaw, Smilja Lambert, Isabella Van Damme, Leonardo D. Gomez, Dimitris Charalampopoulos, Nicholas J. Westwood

**Affiliations:** † School of Chemistry and Biomedical Sciences Research Complex, University of St Andrews and EaStCHEM, North Haugh, St Andrews, Fife, KY16 9ST, United Kingdom; ‡ Department of Food and Nutritional Sciences, University of Reading, Reading, Berkshire, RG6 6AP, United Kingdom; § Centre for Novel Agricultural Products, Department of Biology, University of York, York, North Yorkshire, YO10 5DD, United Kingdom; ∥ Soil Research Centre, Department of Geography and Environmental Sciences, University of Reading, Reading, Berkshire, RG6 6AB, United Kingdom; ⊥ Mars Wrigley Confectionery UK Ltd., Slough, Berkshire, SL1 4LG, United Kingdom; ▽ Mars Wrigley Australia, Ring Road, Wendouree, VIC 3355, Australia

**Keywords:** cocoa pod husk, biorefinery, waste product, organosolv, lignin, flame-retardant, organophosphorus, acetylation

## Abstract

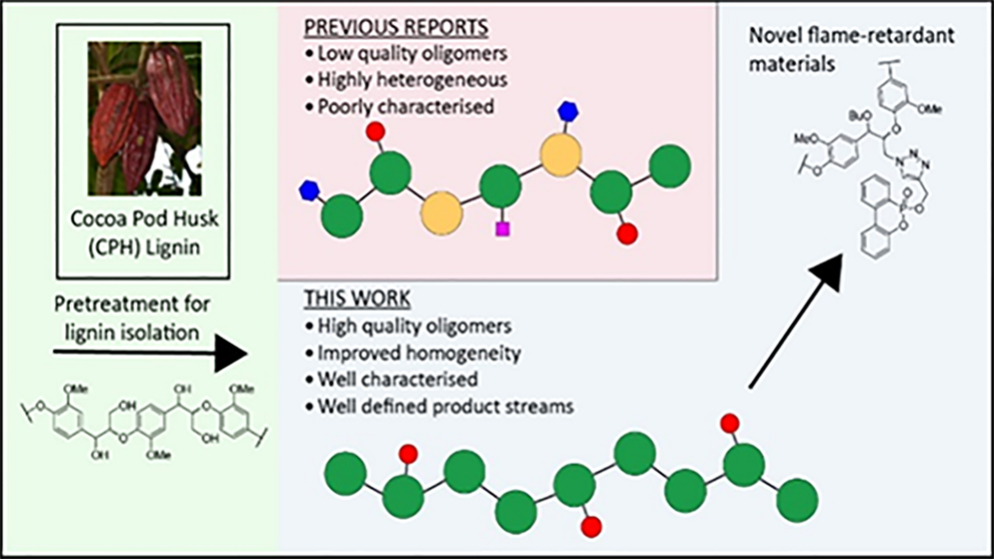

Cocoa pod husks (CPHs) represent an underutilized component of the chocolate manufacturing process. While industry’s current focus is understandably on the cocoa beans, the husks make up around 75 wt % of the fruit. Previous studies have been dominated by the carbohydrate polymers present in CPHs, but this work highlights the presence of the biopolymer lignin in this biomass. An optimized organosolv lignin isolation protocol was developed, delivering significant practical improvements. This new protocol may also prove to be useful for agricultural waste-derived biomasses in general. NMR analysis of the high quality lignin led to an improved structural understanding, with evidence provided to support deacetylation of the lignin occurring during the optimized pretreatment. Chemical transformation, using a tosylation, azidation, copper-catalyzed click protocol, delivered a modified lignin oligomer with an organophosphorus motif attached. Thermogravimetric analysis was used to demonstrate the oligomer’s potential as a flame-retardant. Preliminary analysis of the other product streams isolated from the CPHs was also carried out.

## Abstract 2

Lignin derived from cocoa pod husk waste from chocolate production was isolated, characterized, and modified to produce novel flame-retardant materials.

## Introduction

**Figure 1:**
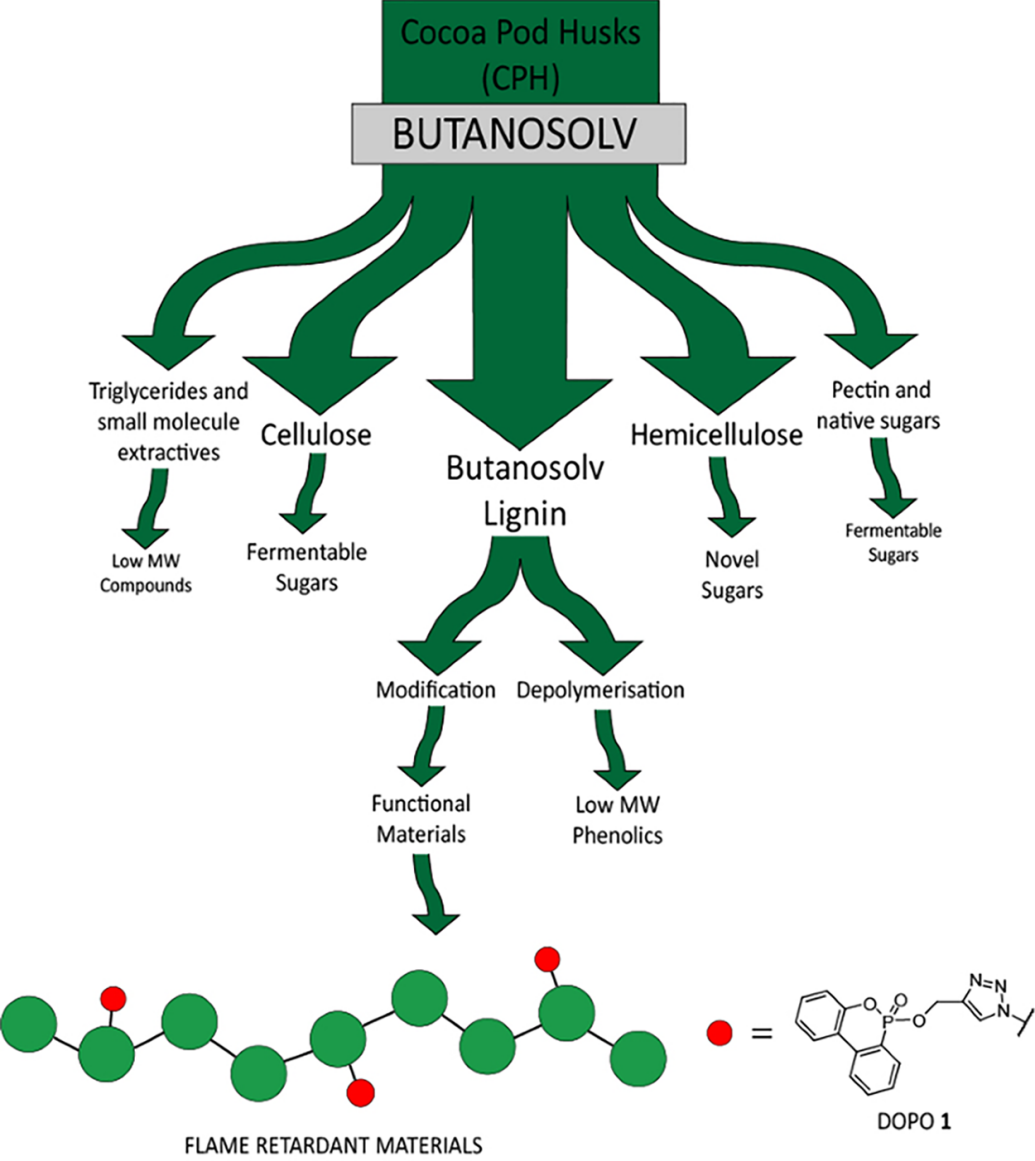
Summary of the fractions obtained during cocoa pod husk processing using the optimized butanosolv pretreatment developed in this report. Potential applications of the isolated fractions are proposed (e.g., conversion of the lignin to a potential flame retardant material).

**Scheme 1:**
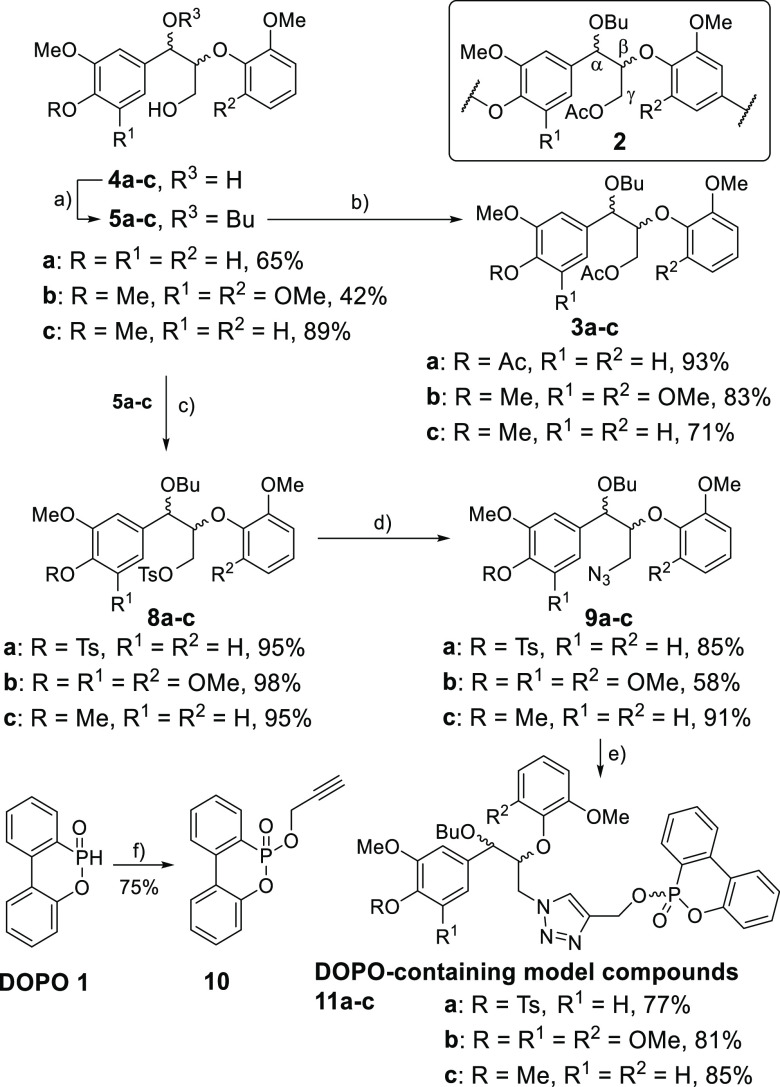
Synthesis of β-O-4 Model Compounds Reagents and conditions: (a) 9:1 BuOH/4M HCl, reflux, 20 min; (b) 10.0 equiv Ac_2_O, Pyr, rt, 8 h; (c) 3.0 equiv TsCl, 3.0 equiv NEt_3_, 0.5 equiv DMAP, DCM, rt, 18 h; (d) 5.0 equiv NaN_3_, DMF, 50 °C, 18 h; (e) 1.1 equiv 7, 1.1 equiv sodium ascorbate, 0.3 equiv CuSO_4_·5H_2_O, MeOH, rt, 12 h; (f) (i) 1.1 equiv NCS, toluene, rt, 18 h; (ii) 1.1 equiv NEt_3_, 1.1 equiv propargyl alcohol, DCM, rt, 18 h.

Biorefining, the process by which renewable biomass feedstocks are converted into marketable products in an integrated manner, remains a challenge.^1^ In several biorefinery designs, a pretreatment process is used to simplify the starting biomass by addressing both its recalcitrant nature and its inherent complexity (Figure 1).^2−8^ While a wide range of approaches to biomass pretreatment exist,^9−14^ the use of mild organosolv pretreatments^15−20^ often delivers high quality intact lignin as well as cellulose, hemicellulose-derived, and other fractions. Consideration of possible uses for an intact organosolv lignin illustrates the flexibility inherent in using a pretreatment strategy. Potential lignin applications range from alternative lignin depolymerization protocols that give different aromatic monomers,^21,22^ to a number of approaches for building on the existing lignin template.^22−25^ For example, studies have described the preparation of flame retardant materials from lignin.^26,27^ These recent reports have inspired us to describe our complementary studies on the incorporation of 9,10-dihydro-9-oxa-10-phosphaphenanthrene-10-oxide (DOPO) **1** into lignin. DOPO **1** is an organophosphorus molecule known to be important in fire management strategies^28^ (Figure 1 and Scheme 1).

Another part of the biorefinery challenge is its application to less mainstream biomass sources. While many studies use soft or hardwoods, researchers continue to explore less common biomass. Recent reports have described, for example, the processing of coffee husks,^29^ rice husks,^30^ and pomegranate peel,^31^ but the list of possible starting materials continues to increase.^7^ Here the focus is on an understudied waste product from the chocolate industry: the cocoa pod husk (CPH). The total global harvest of cocoa beans for the 2021/22 growing season was 4.8 million tonnes with this estimated to increase slightly for the 2022/23 growing season.^32^ As the CPH represents the bulk (70–75%) of the fruit produced by the *Theobroma cacao* tree, this means that more than 20 million tonnes of cocoa pod husks are produced p.a. worldwide. The husk is usually left on the farm to biodegrade. However, the value of this practice is contested, as it may have an overall negative impact by allowing proliferation of the “*cacao* disease trilogy”.^33,34^ Some cocoa farmers are considering preparing and selling CPH for alternative uses if collection is feasible and the price is sufficient.^35^ In terms of the current technology associated with CPH biorefining, reports have focused on the analysis and application of carbohydrate components,^36,37^ particularly studies on CPH pectin.^38−42^ Here, while a number of fractions from the biomass are considered, we focus instead on the CPH lignin component. Limited precedent for the isolation of lignin from CPH is available which is surprising given that current estimates of lignin content place it at around 20% of the weight of the husk.^43−46^

Following a preliminary comparison of potential methods for the efficient pretreatment of CPH (Figures S1–S3), an optimized protocol for the butanosolv pretreatment of CPH was developed. This protocol delivered a high quality lignin that was characterized in detail providing novel insights into its structure and purity. In addition, the isolated lignin was used to prepare a potential flame-retardant material. Preliminary characterization of the six other product streams (Figure 1) is also included. While it remains unclear what the optimal way to process CPHs is, our approach delivered a high quality lignin and a number of other defined product streams for further study.

## Results and Discussion

### Optimized Isolation of Butanosolv Lignin from CPH

**Figure 2:**
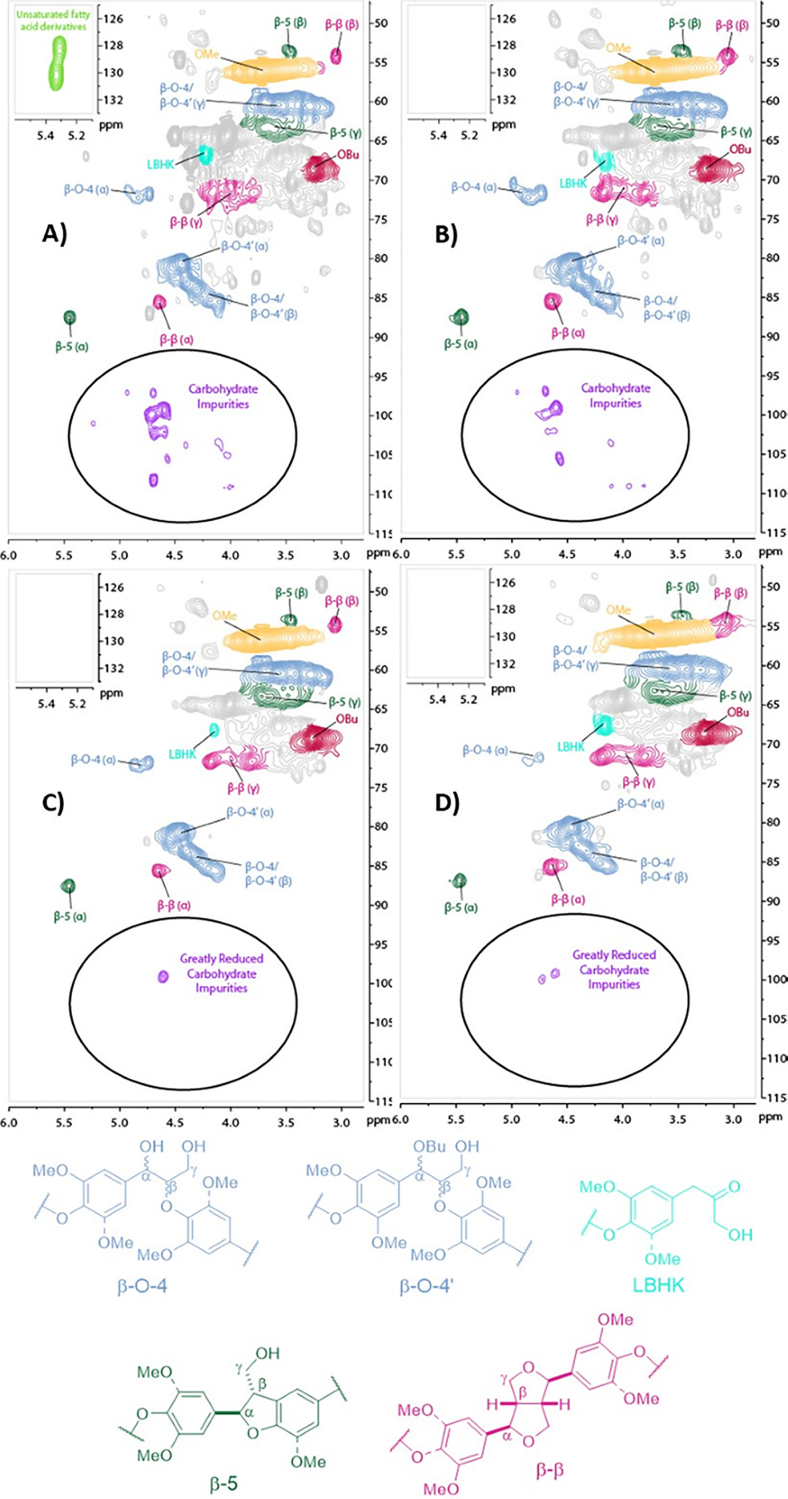
HSQC NMR (700 MHz, DMSO-*d*_6_) analysis of Cocoa Pod Husk (CPH) lignin obtained by butanosolv pretreatment using a literature method:^47^ (A) before the final purification step; (B) after purification by reprecipitation using organic solvents; (C) after purification using an alternative caustic soda purification method.^52^ (D) Analysis of the CPH lignin obtained using the optimized butanosolv pretreatment developed in this work for comparison. The relevant structures that correspond to interunit linkages are shown. The aromatic regions of the HSQC NMR spectra are shown in Figure S4.

A mild butanosolv pretreatment for use with soft or hardwoods typically involves the heating of a suspension of the biomass in 95% butanol/5% aqueous hydrochloric acid at reflux for 6 h. This literature protocol^47^ was applied to CPH biomass to obtain a butanosolv CPH lignin. A sample of this lignin was analyzed by HSQC NMR^48,49^ prior to the final purification step to assess impurity levels. Signals corresponding to unsaturated fatty acid derivatives,^50^ likely based on linoleic acid,^51^ and aryl-ring containing small molecules were the major contaminants (*c.f.*Figures 2A,B and S4A,B for analysis of lignin prior to and postpurification). Washing the starting biomass with ethanol before butanosolv pretreatment reduced the number of impurities and resulted in a lignin with improved purity (*c.f.*Figures 2B and S4B,E,F). The ethanol prewash also resolved practical challenges associated with precipitation of the lignin, and this step was therefore incorporated into an “optimized pretreatment” method.

Carbohydrates are known impurities in butanosolv lignins and recent studies^52^ have shown that caustic soda treatment of walnut shell butanosolv lignin decreased carbohydrate contamination. A modified caustic soda treatment was applied to the lignin to assess its effect on lignin purity. The lignin was recovered in good yield and had a lower carbohydrate content as expected^52^ (Figures 2C and S4C) while maintaining high levels of β-O-4 content. No debutoxylation of the lignin was observed, in contrast to the previous report.^52^ The addition of this step did provide a higher purity lignin; however, alternatives were also considered.

The efficient aqueous extraction of one of the major carbohydrates in CPH, pectin, has been reported.^38−44^ In our study, hot water extraction of CPH prior to butanosolv pretreatment was found to decrease the carbohydrate content in the isolated lignin (*c.f.*Figures 2B and S4G). Pectin and a second fraction that contained fermentable sugars (referred to here as PESF) were also obtained. By combining (i) the ethanol prewash with (ii) the hot aqueous extraction and (iii) butanosolv pretreatment, a CPH lignin was obtained in 3.7 wt % that retained a high β-O-4 content and had low carbohydrate and fatty acid contaminants (Figure 2D, Table S1). In addition, all the practical challenges encountered on applying the literature pretreatment^47^ to CPH were addressed. For example, the lignin from the optimized pretreatment was a fine powder easily isolated by filtration (Figure S5). DOSY NMR analysis demonstrated that the diffusivity and estimated MW of the lignin obtained did not vary as the changes in the pretreatment were made^53^ (Figure S6 and Tables S2 and S3). The MW values were within error (e.g., MW = 3700 ± 900 Da for the lignin from the optimized pretreatment). The optimized pretreatment was suitable for lignin generation from CPH biomass and seems likely to be applicable to a range of nonwoody biomasses.

### Further Analysis of the Lignin

**Figure 3:**
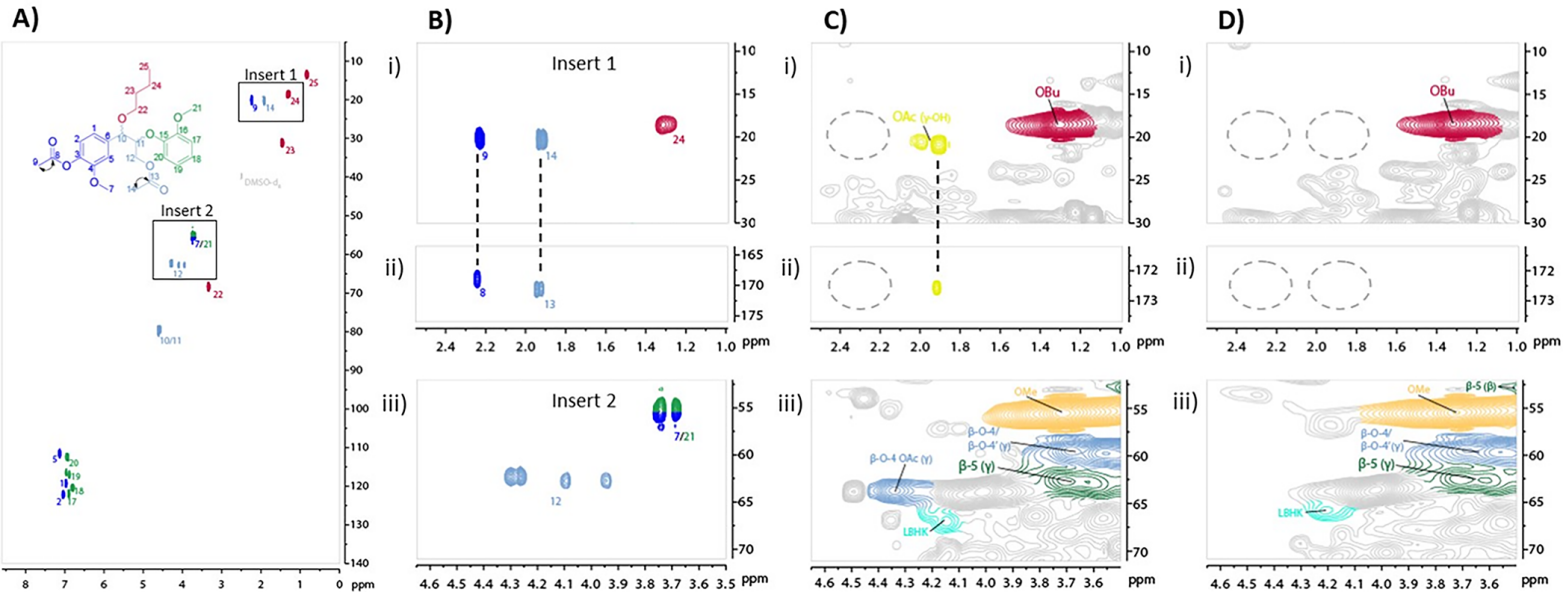
(A) HSQC NMR (700 MHz, DMSO-*d*_6_) analysis of acetylated β-O-4 model **3a**. Signal assignments are numbered and color-coded. Relevant HMBC correlations are indicated by arrows; (B) expansion of (i) the alkyl region shown in A (insert 1) and corresponding signals in the (ii) HMBC NMR and (iii) the modified γ-primary alcohol region shown in A (insert 2); (C) expansion of a region of the HSQC NMR (700 MHz, DMSO-*d*_6_) analysis of CPH lignin isolated using the literature pretreatment^47^ showing (i) alkyl region with signals corresponding to the methyl group of acetylated β-O-4 linkages indicated (yellow), (ii) the corresponding carbonyl signals in band selective HMBC NMR analysis, and (iii) expansion of modified γ-primary alcohol region with signals unambiguously assigned as corresponding to acetylated β-O-4 linkages indicated (blue); (D) expansion of HSQC NMR (700 MHz, DMSO-*d*_6_) analysis of CPH lignin from the optimized pretreatment showing (i) alkyl region with no signals corresponding to acetylated β-O-4 linkages, (ii) the corresponding absence of signals in the HMBC NMR analysis, and (iii) expansion of modified γ-primary alcohol region with no signals corresponding unambiguously to acetylated β-O-4 linkages. Lignin from both the literature^47^ and optimized protocols show no signals corresponding to acetylation at terminal phenolic oxygen positions. Regions in light gray are unassigned or cannot be assigned to a single structural feature in the lignin (see Figures S7–S9 for a more detailed discussion). Signals in the region ^1^H 4.55–3.85/^13^C 65.0–62.5 are known to correspond to cinnamate, *p*-coumarate,^54,55^ and *p*-hydroxybenzoate esters.^56,57^

In addition to increasing the purity of the lignin, use of the optimized pretreatment also altered the CPH lignin’s structure. Comparison of HSQC NMR spectra found that signals at ^1^H: 4.34/^13^C: 63.7 ppm and ^1^H: 1.96/^13^C: 20.7 ppm were present only in the lignin obtained from the literature pretreatment^47^ (Figure 3C,D). This observation was rationalized based on the presence or absence of an acetylated β-O-4 unit (acetylated on the primary γ-hydroxy group, generalized structure **2**, Scheme 1) in the lignin. Novel model compounds **3a**–**c** were prepared (via **4a**–**c** and **5a**–**c**, Scheme 1) and comparison of their NMR spectra with those of the lignin confirmed that **2** was only present in the lignin obtained using the literature pretreatment^47^ and NOT the optimized pretreatment (Figure 3A,B for **3a** and Figures S7–S9). This fortuitous difference in lignin structure helps the lignin modification studies discussed below, as more β-O-4 γ-hydroxyls are available when the optimized pretreatment is used. The controlled removal of acetyl and other ester groups from the β-O-4 γ-hydroxy position in lignins has been explored previously (e.g., triethylamine-catalyzed de-esterification on Birch bark biomass,^58^ and deacetylation of Poplar biomass using deep eutectic solvents^59^), although not with CPH lignin.

### Modification of CPH Lignin

**Scheme 2:**
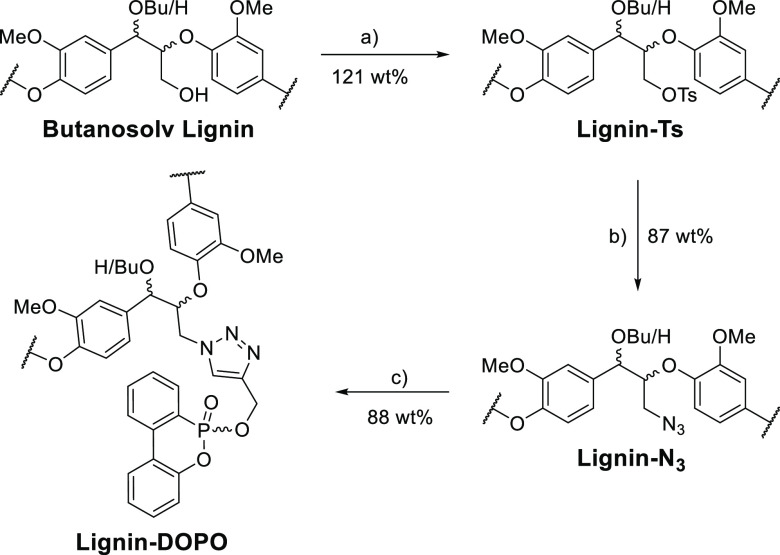
Synthetic Procedure Used to Convert CPH Lignin to DOPO-Modified Lignin Reagents and conditions: (a) 2.25 wt equiv TsCl, Pyr, rt, 18 h; (b) 5 wt equiv NaN_3_, DMF, 50 °C, 18 h; (c) 0.5 wt equiv **10**, 0.5 wt equiv sodium ascorbate, 0.03 wt equiv CuSO_4_·5H_2_O, 5:1 DMF/H_2_O, rt, 18 h.

**Figure 4:**
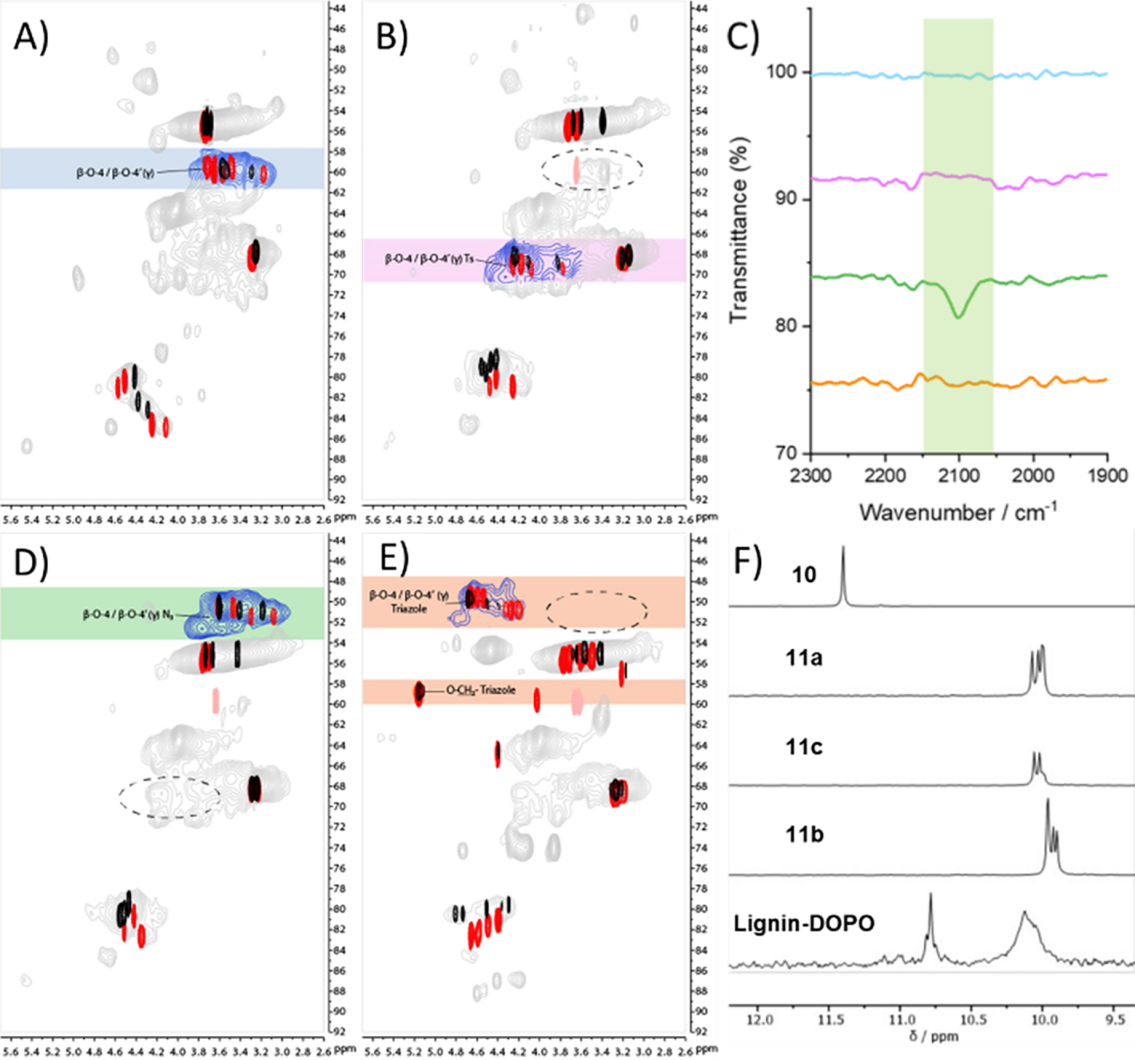
Analysis of modified lignins. HSQC NMR (700 MHz, DMSO-*d*_6_) analysis of the linkage region of the following: (A) Starting CPH lignin with signals corresponding to the CH_2_ protons of the unmodified β-O-4 γ-alcohols highlighted (blue), overlaid with NMR analysis of model compounds **5a** (black) and **5b** (red). See Schemes 1 and 2 for all chemical structures. (B) Lignin-Ts with signals corresponding to the CH_2_ protons of the tosyl modified β-O-4 γ-alcohols highlighted (pink), overlaid with model compounds **8a** (black) and **8b** (red). (C) FTIR spectra of CPH butanosolv lignin (blue), Lignin-Ts (pink), Lignin-N_3_ (green), and Lignin-DOPO (orange). Highlighted region shows appearance of azide stretching frequency at 2100 cm^–1^ in Lignin-N_3_ and disappearance in Lignin-DOPO indicating CuAAC click reaction was successful. (D) Lignin-N_3_ with signals corresponding to the CH_2_ protons of the azide modified β-O-4 γ-alcohols highlighted (green), overlaid with model compounds **9a** (black) and **9b** (red). (E) Lignin-DOPO with signals corresponding to CH_2_ protons of the DOPO-triazole modified β-O-4 γ-alcohols highlighted (orange), overlaid with model compounds **11a** (black) and **11b** (red). Signal at ^1^H 4.40/^13^C 64.4 ppm in **11a** corresponds to the O–CH_2_–triazole signal in a minor stereoisomer not observed in the lignin. (F) ^31^P NMR spectra (202 MHz, DMSO-*d*_6_) of **10**, **11a**–**c**, and Lignin-DOPO. The pale pink signal in B, D, and E corresponds to the additional methoxy group at the 4-position of model compounds that is not present in the lignins. The aromatic regions of CPH lignin, Lignin-Ts, Lignin-N_3_, and Lignin-DOPO are shown in Figure S10.

The use of lignin as a template for novel oligomers/materials synthesis is increasingly reported, for example the use of lignin as a scaffold in a variety of resins.^60−63^ In this case, CPH Lignin from the optimized pretreatment was modified (Scheme 2) to give first a tosylated lignin (Lignin-Ts, *c.f.*Figures 4A,B, S10A, and S4D) and then an azidated lignin (Lignin-N_3_, Figure 4D and S10B). The reactions were monitored using HSQC NMR, including a comparison with the spectra of the synthesized model compounds. For example, the conversion of CPH lignin to Lignin-Ts was confirmed using model compounds **8a** and **8b** (Figure 4B and Scheme 1 for structures). Lignin-N_3_ (*c.f.***9a** and **9b**, Figure 4D) was then reacted in a Cu-catalyzed alkyne–azide cycloaddition (CuAAC) reaction with the previously used 1-nitro-4-(prop-2-yn-1-yloxy)benzene (Figure S10D,E),^60^ confirming that the CPH lignin was a viable substrate.

Lignin-N_3_ was then reacted with the DOPO-derivative **10** (Schemes 1 and 2). DOPO **1** and its derivatives are incorporated into polymers to introduce flame-retardant properties via char formation, with their use offering a greener alternative to halogenated flame retardants.^64^ The preparation of Lignin-DOPO was confirmed by IR (Figure 4C), HSQC NMR (Figure 4E and S10C, *c.f.***11a** and **11b**) and ^31^P NMR analysis (Figure 4F). The broad signal in the ^31^P NMR spectrum (10.4–9.8 ppm) was consistent with the attachment of the DOPO unit onto the lignin with additional broadness resulting from the modification of G- and S-containing β-O-4 units.

**Figure 5:**
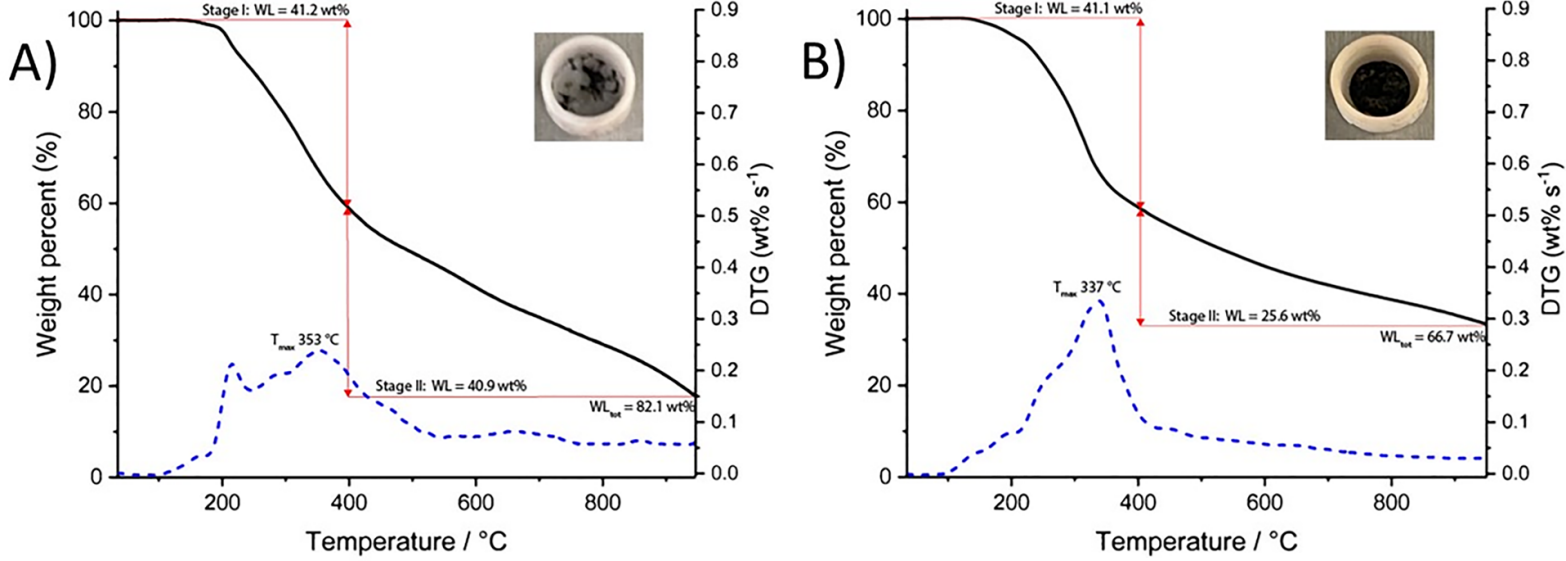
TGA (black) and DTG (blue, dashed) curves (N_2_, 10 °C/min) obtained (A) CPH lignin and (B) Lignin-DOPO. The percentage weight loss of each sample during stage I and stage II pyrolysis is given by the red lines.^65,66^ The TGA curve for CPH lignin was directly comparable with the control lignin sample prepared from an alkyne without a phosphorus-containing unit (Figure S11), emphasizing the importance of the P-containing DOPO motif in the char formation and not the triazole motif. The char residues recovered after thermogravimetric analysis are shown (inset); CPH lignin and a second control lignin (Figure S11) gave little to no residue, while Lignin-DOPO gave a pellet of char.

Thermogravimetric analysis (TGA) was carried out to assess the flame-retardant properties of CPH Lignin-DOPO. The TGA curves of lignin and Lignin-DOPO (Figure 5) showed similar expected mass losses (41.2 and 41.1 wt %) during stage I pyrolysis between 200 and 400 °C, where alkyl C–O bonds in interunit ether linkages are cleaved.^65,66^ During stage II pyrolysis above 400 °C, O–Me bonds are first broken, giving increased phenolic content, followed by cleavage and rearrangement of aromatic C–O and C–C bonds, ultimately leading to gasification.^65,66^ For lignin, the stage II pyrolysis led to a large mass loss (40.9 wt %), giving a total loss of 82.1 wt % at 950 °C. This sample underwent further mass loss as it cooled, leading to effectively no residual material (Figure 5A, inset). Lignin-DOPO behaved differently in the stage II pyrolysis, with a smaller mass loss (25.6 wt %) observed. This gave a lower overall mass loss of 66.7 wt % at 950 °C.

The temperature at which the greatest rate of mass loss occurred, *T*^max^, was obtained from DTG curves^66^ and was comparable for the lignin and Lignin-DOPO (353 and 337 °C, respectively), occurring during stage I pyrolysis. Attachment of DOPO-derivative **10** presumably promoted char formation on the surface of the sample during stage II pyrolysis to inhibit further decomposition. A char was recovered following TGA of Lignin-DOPO (Figure 5B, inset), supportive of potential flame-retardant properties that are not inherent to the lignin itself. Comparison with the TGA and DTG curves of a control lignin, prepared by reaction with an alternative alkyne that did not contain a phosphorus-based unit (Figure S11), highlighted the importance of the organophosphorus DOPO motif in the flame-retardant properties. While these preliminary results were encouraging, future work will focus on the scaled-up synthesis and testing of CPH Lignin-DOPO.

### Analysis of the Additional Fractions Obtained Using the Optimized Pretreatment

**Figure 6:**
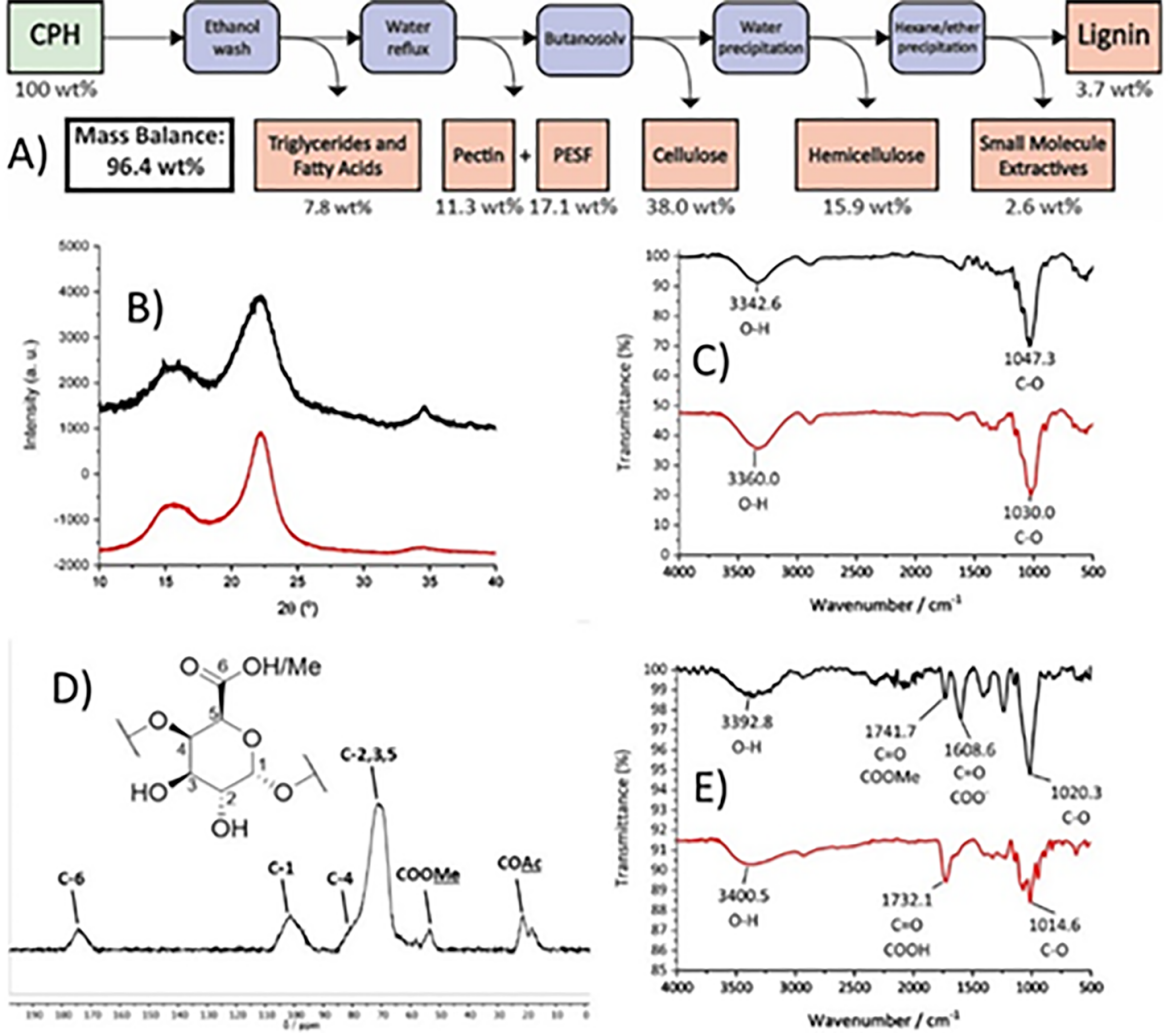
(A) Summary of fractions produced from the optimized pretreatment and the observed mass balance. PESF = pectin ethanol soluble fraction; (B) PXRD pattern of the cellulose pulp (black) and a commercial sample of cellulose I (red); (C) FTIR spectra of the cellulose pulp (black) and a commercial sample of cellulose I (red); (D) Solid-state ^13^C CP/MAS NMR spectrum of pectin obtained from the optimized pretreatment; (E) FTIR spectra of pectin (black) and a commercial sample of polygalacturonic acid (red) used as a model for a homogalactan pectin.

In the final stage of this study, more detailed analysis of several of the other fractions generated in the optimized pretreatment of CPH was carried out (Figures 6A and S12). In brief, the powder X-ray diffraction pattern of the CPH cellulose pulp was compared to that of a commercial sample of cellulose I (Figure 6B).^67^ This technique, FTIR studies (Figure 6C), and acetyl bromide derivatization (which solubilized the pulp to enable assessment of potential lignin content by solution-state 2D HSQC NMR,^68^Figure S13), indicated that the cellulose formed was lignin-free and type I.

The monosaccharide composition of the pectin was determined (HPAEC analysis, Figure S14 and Table S4) with galacturonic acid being the major component, as expected.^44^ FTIR analysis of the pectin showed a signal at 1608 cm^–1^ (Figure 6E, black) assigned to carboxylate (COO^–^) groups.^69^ This requires the presence of metal counterions (possibly K^+^ given the large amount present in CPH,^35^Table S5). In contrast, carboxylic acid (COOH) functional groups were present in a commercial sample of a model pectin, polygalacturonic acid (Figure 6E, red, peak at 1732 cm^–1^). The pectin was also analyzed using solid-state ^13^C NMR, allowing the calculation of the degree of methylation (DM) using the method of Zhu et al.^70^ (Figure 6D). The pectin that was extracted here using hot water was a low-methoxy pectin, in contrast to previous reports that used different extraction conditions.^43^ The signal at 21.2 ppm (Figure 6D) was consistent with pectin acetylation^70^ (Figures S15 and S16 and Table S6). The second fraction obtained during pectin removal (PESF) was surprisingly abundant (17.1 wt %, Figure 6A). Its monosaccharide composition was determined (Figure S14 and Table S4) with glucose being the most abundant. NMR analysis indicated that PESF was a complex mixture of oligo- and polysaccharides, with both α-glucose and α-mannose units present (Figure S17).

Despite the ethanol prewash, fatty acid derivatives were also found in the filtrate obtained during the final lignin purification step (Figure S18). However, the major component (73%) of the filtrate was a sample of lower MW lignin (Table S3), which was isolated by column chromatography. If taken into account, this extra lignin increased the total lignin yield from 3.7 to 5.4 wt %. Recovery of two batches of lignin with different MWs showed that the CPH lignin was fractionated in the final purification step and could likely be fractionated further.^71,72^

## Conclusions

Many challenges remain as biorefinery development continues. Here high quality lignin was isolated from cocoa pod husks (CPHs), a waste product connected with chocolate production. No previous studies have reported details of the structure and reactivity. Reasonable yields of lignin were obtained, although significantly less lignin was isolated than expected based on literature reports.^35,44,46,73,74^ This discrepancy may reflect an overestimation of the isolable lignin content in CPH. The isolated lignin was in a deacetylated form, and this facilitated its modification at the γ-position of the β-O-4 unit. Use of novel model compounds confirmed the structure of the modified lignin, and thermogravimetric analysis highlighted interesting potential flame-retardant properties. The optimized pretreatment also led to the formation of six other potential product streams, several of which were studied. While detailed LCA, techno-economic, and agronomic analysis of the reported process is outside the scope of this report, a recently reported study discusses several key aspects of developing a conceptual novel value chain from CPH (which could lead to a number of products, such as modified lignin or lignin-derived aromatics, ethanol, food ingredients, etc.).^33^ This report assessed (i) the economic viability of CPH valorization from a farmer’s perspective and (ii) the consequences on soil quality of diverting CPH from its role as a natural fertilizer through an agronomic trial.

Based on preliminary work reported here, future application of high quality CPH lignin will focus on the production of novel flame-retardant materials (lignin-DOPO). This application for lignin is of current interest and this work provides complementary methodology to recent alternative approaches.^26,27^

## Experimental Section

### Materials

The cocoa pod husk biomass material was provided by Mars Wrigley Confectionery from the Mars Cocoa Research Station in Indonesia. Cocoa pod husk (CPH) biomass was frozen upon arrival, then defrosted just prior to milling, and was milled using a Retsch SM 300 SM mill equipped with a 1 mm screen. The milled CPH was stored frozen and defrosted just prior to use in pretreatment protocols. Commercially available compounds were purchased and used as received unless otherwise stated in the SI.

### Methods

Full description of the pretreatment methods is given in the General Methods section of the SI. Optimized butanosolv pretreatment for CPH: CPH biomass was suspended in ethanol (10 mL/g) and stirred at room temperature for 18 h. The suspension was then filtered, and the recovered CPH pulp was suspended in fresh ethanol (10 mL/g) and stirred for an additional 4 h. The suspension was filtered and the CPH pulp dried in vacuo at 60 °C for 24 h. The dried CPH pulp was then suspended in water (25 mL/g) and heated at reflux for 4 h. The suspension was cooled to room temperature, centrifuged at 5500 rpm at 4 °C for 1 h, and strained through cheesecloth, washed with fresh water (3 × 10 mL/g), and squeezed until dry. The CPH pulp was dried in vacuo at 60 °C for 24 h and then butanosolv pretreatment was carried out according to a literature procedure.^47^ Tosylation and azidation reactions were carried out according to a literature procedure.^47^ For the CuAAC click reactions, based on a literature procedure,^47^ azidated butanosolv lignin (1 wt equiv), novel DOPO alkyne derivative **11** (0.5 wt equiv), sodium ascorbate (0.5 wt equiv), and CuSO_4_·5H_2_O (0.03 wt equiv) were stirred in DMF/water (5:1, 10 mL/g of lignin) at room temperature for 24 h. The solution was added dropwise to 0.1 M HCl (10 v/v equiv), and the resulting precipitate isolated by filtration, washed with water (30 mL/g), and dried under vacuum at 60 °C for 24 h. The crude lignin was purified by column chromatography on silica gel (30 g/g) eluting with DCM/hexane (0–100%), MeOH/DCM (0–10%), and then 100% acetone. See SI for full details for the synthesis and analytical characterization of the model compounds.

## Supporting Information Available

Supplementary figures mentioned throughout the text, including additional NMR spectra, synthetic procedures for model compounds, characterization of novel compounds and associated spectra, and full lignin HSQC NMR spectra (PDF)
Crystallographic data of **10** (CIF)

## Supplementary Material

sc2c03670_si_001.pdf

sc2c03670_si_002.cif

## Dedication

This manuscript is dedicated to the late Prof. Simon McQueen-Mason.

## Abbreviations

DM: degree of methylation
FTIR: Fourier transform infrared
HMBC: heteronuclear multiple bond correlation spectroscopy
HSQC: heteronuclear single quantum coherence spectroscopy
MW: molecular weight
NMR: nuclear magnetic resonance
PESF: pectin ethanol soluble sugar fraction
PXRD: powder X-ray diffraction

## References

[ref1] Espinoza Pérez A. T.; Camargo M.; Narváez Rincón P. C.; Alfaro Marchant M. Key Challenges and Requirements for Sustainable and Industrialized Biorefinery Supply Chain Design and Management: A Bibliographic Analysis. Renewable and Sustainable Energy Reviews 2017, 69, 350–359. 10.1016/j.rser.2016.11.084.

[ref2] Zakzeski J.; Bruijnincx P. C. A.; Jongerius A. L.; Weckhuysen B. M. The Catalytic Valorization of Lignin for the Production of Renewable Chemicals. Chem. Rev. 2010, 110 (6), 3552–3599. 10.1021/cr900354u. 20218547

[ref3] Alonso D. M.; Wettstein S. G.; Dumesic J. A. Bimetallic Catalysts for Upgrading of Biomass to Fuels and Chemicals. Chem. Soc. Rev. 2012, 41, 8075–8098. 10.1039/c2cs35188a. 22872312

[ref4] Prasad B. R.; Padhi R. K.; Ghosh G. A review on key pretreatment approaches for lignocellulosic biomass to produce biofuel and value-added product. International Journal of Environmental Science and Technology 2023, 20 (6), 6929–6944. 10.1007/s13762-022-04252-2.

[ref5] Yoo C. G.; Meng X.; Pu Y.; Ragauskas A. J. The critical role of lignin in lignocellulosic biomass conversion and recent pretreatment strategies: A comprehensive review. Bioresour. Technol. 2020, 301, 122784 10.1016/j.biortech.2020.122784. 31980318

[ref6] Pre-treatments to enhance the enzymatic saccharification of lignocellulose: technological and economic aspects. https://www.bbnet-nibb.co.uk/wp-content/uploads/2021/02/BBNet-Pretreatment-Tech-Review-Feb2021-.pdf.

[ref7] Dharmaraja J.; Shobana S.; Arvindnarayan S.; Francis R. R.; Jeyakumar R. B.; Saratale R. G.; Ashokkumar V.; Bhatia S. K.; Kumar V.; Kumar G. Lignocellulosic biomass conversion via greener pretreatment methods towards biorefinery applications. Bioresour. Technol. 2023, 369, 128328 10.1016/j.biortech.2022.128328. 36402280

[ref8] Baruah J.; Nath B. K.; Sharma R.; Kumar S.; Deka R. C.; Baruah D. C.; Kalita E. Recent Trends in the Pretreatment of Lignocellulosic Biomass for Value-Added Products. Frontiers in Energy Research 2018, 6, 141 10.3389/fenrg.2018.00141.

[ref9] Mankar A. R.; Pandey A.; Modak A.; Pant K.K. Pretreatment of lignocellulosic biomass: A review on recent advances. Bioresour. Technol. 2021, 334, 125235 10.1016/j.biortech.2021.125235. 33957458

[ref10] van Aelst K.; van Sinay E.; Vangeel T.; Cooreman E.; van den Bossche G.; Renders T.; van Aelst J.; van den Bosch S.; Sels B. F. Reductive Catalytic Fractionation of Pine Wood: Elucidating and Quantifying the Molecular Structures in the Lignin Oil. Chemical Science 2020, 11 (42), 11498–11508. 10.1039/D0SC04182C. 34094394 PMC8162782

[ref11] Bartling A. W.; Stone M. L.; Hanes R. J.; Bhatt A.; Zhang Y.; Biddy M. J.; Davis R.; Kruger J. S.; Thornburg N. E.; Luterbacher J. S.; Rinaldi R.; Samec J. S. M.; Sels B. F.; Román-Leshkov Y.; Beckham G. T. Techno-Economic Analysis and Life Cycle Assessment of a Biorefinery Utilizing Reductive Catalytic Fractionation. Energy Environ. Sci. 2021, 14 (8), 4147–4168. 10.1039/D1EE01642C. 36324336 PMC9562980

[ref12] Jindal M.; Uniyal P.; Thallada B. Reductive catalytic fractionation as a novel pretreatment/lignin-first approach for lignocellulosic biomass valorization: A review. Bioresour. Technol. 2023, 385, 129396 10.1016/j.biortech.2023.129396. 37369316

[ref13] Raikwar D.; Van Aelst K.; Vangeel T.; Corderi S.; Van Aelst J.; Van den Bosch S.; Servaes K.; Vanbroekhoven K.; Elst K.; Sels B. F. Elucidating the effect of the physicochemical properties of organosolv lignin on its solubility and reductive catalytic depolymerisation. Chem. Eng. J. 2023, 461, 141999–142016. 10.1016/j.cej.2023.141999.

[ref14] Lu X.; Lagerquist L.; Eranen K.; Hemming J.; Eklund P.; Estel L.; Leveneur S.; Grenman H. Reductive Catalytic Depolymerization of Semi-industrial Wood-Based Lignin. Ind. Eng. Chem. Res. 2021, 60 (47), 16827–16838. 10.1021/acs.iecr.1c03154. 34880549 PMC8641393

[ref15] Meng X.; Wang Y.; Conte A. J.; Zhang S.; Ryu J.; Wie J. J.; Pu Y.; Davison B. H.; Yoo C. G.; Ragauskas A. J. Applications of biomass-derived solvents in biomass pretreatment - Strategies, challenges, and prospects. Bioresour. Technol. 2023, 368, 128280 10.1016/j.biortech.2022.128280. 36368492

[ref16] Borand M. N.; Karaosmanoglu F. Effects of Organosolv Pretreatment Conditions for Lignocellulosic Biomass in Biorefinery Applications: A Review. Journal of Renewable and Sustainable Energy 2018, 10 (3), 033104 10.1063/1.5025876.

[ref17] Shuai L.; Amiri M. T.; Questell-Santiago Y. M.; Héroguel F.; Li Y.; Kim H.; Meilan R.; Chapple C.; Ralph J.; Luterbacher J. S. Formaldehyde Stabilization Facilitates Lignin Monomer Production during Biomass Depolymerization. Science 2016, 354 (6310), 329–333. 10.1126/science.aaf7810. 27846566

[ref18] Yong K. J.; Wu T. Y. Recent advances in the application of alcohols in extracting lignin with preserved β-O-4 content from lignocellulosic biomass. Bioresour. Technol. 2023, 384, 129238 10.1016/j.biortech.2023.129238. 37245662

[ref19] vom Stein T.; Grande P. M.; Kayser H.; Sibilla F.; Leitner W.; Domínguez de María P. From Biomass to Feedstock: One-Step Fractionation of Lignocellulose Components by the Selective Organic Acid-Catalyzed Depolymerization of Hemicellulose in a Biphasic System. Green Chem. 2011, 13 (7), 1772–1777. 10.1039/c1gc00002k.

[ref20] Grande P. M.; Viell J.; Theyssen N.; Marquardt W.; Domínguez De María P.; Leitner W. Fractionation of Lignocellulosic Biomass Using the OrganoCat Process. Green Chem. 2015, 17 (6), 3533–3539. 10.1039/C4GC02534B.

[ref21] Sun Z.; Fridrich B.; de Santi A.; Elangovan S.; Barta K. Bright Side of Lignin Depolymerization: Toward New Platform Chemicals. Chem. Rev. 2018, 118, 614–678. 10.1021/acs.chemrev.7b00588. 29337543 PMC5785760

[ref22] Deuss P. J.; Lahive C. W.; Lancefield C. S.; Westwood N. J.; Kamer P. C. J.; Barta K.; de Vries J. G. Metal Triflates for the Production of Aromatics from Lignin. ChemSusChem 2016, 9 (20), 2974–2981. 10.1002/cssc.201600831. 27650221

[ref23] Lawoko M.; Berglund L.; Johansson M. Lignin as a Renewable Substrate for Polymers: From Molecular Understanding and Isolation to Targeted Applications. ACS Sustainable Chem. Eng. 2021, 9 (16), 5481–5485. 10.1021/acssuschemeng.1c01741.

[ref24] Zhao S.; Abu-Omar M. M. Materials Based on Technical Bulk Lignin. ACS Sustainable Chem. Eng. 2021, 9 (4), 1477–1493. 10.1021/acssuschemeng.0c08882.

[ref25] Bertella S.; Luterbacher J. S. Lignin Functionalization for the Production of Novel Materials. Trends in Chemistry 2020, 2 (5), 440–453. 10.1016/j.trechm.2020.03.001.

[ref26] Zhang Y. M.; Zhao Q.; Li L.; Yan R.; Zhang J.; Duan J. C.; Liu B. J.; Sun Z. Y.; Zhang M. Y.; Hu W.; Zhang N. N. Synthesis of a Lignin-Based Phosphorus-Containing Flame Retardant and Its Application in Polyurethane. RSC Adv. 2018, 8 (56), 32252–32261. 10.1039/C8RA05598J. 35547477 PMC9086252

[ref27] Lu X.; Yu M.; Wang D.; Xiu P.; Xu C.; Lee A. F.; Gu X. Flame-Retardant Effect of a Functional DOPO-Based Compound on Lignin-Based Epoxy Resins. Materials Today Chemistry 2021, 22, 100562 10.1016/j.mtchem.2021.100562.

[ref28] Salmeia K. A.; Gaan S. An overview of some recent advances in DOPO-derivatives: Chemistry and flame retardant applications. Polym. Degrad. Stab. 2015, 113, 119–134. 10.1016/j.polymdegradstab.2014.12.014.

[ref29] Massaya J.; Chan K. H.; Mills-Lamptey B.; Chuck C. J. Developing a Biorefinery from Spent Coffee Grounds Using Subcritical Water and Hydrothermal Carbonisation. Biomass Conversion and Biorefinery 2023, 13, 1279–1295. 10.1007/s13399-020-01231-w.

[ref30] Offei F.; Koranteng L. D.; Kemausuor F. Integrated Bioethanol and Briquette Recovery from Rice Husk: A Biorefinery Analysis. Biomass Conversion and Biorefinery 2023, 13, 7645–76611. 10.1007/s13399-021-01731-3.

[ref31] Talekar S.; Patti A. F.; Vijayraghavan R.; Arora A. An Integrated Green Biorefinery Approach towards Simultaneous Recovery of Pectin and Polyphenols Coupled with Bioethanol Production from Waste Pomegranate Peels. Bioresour. Technol. 2018, 266, 322–334. 10.1016/j.biortech.2018.06.072. 29982054

[ref32] ICCO Quarterly Bulletin of Cocoa Statistics, Vol. XLIX, No. 2, Cocoa year 2022/23. https://www.icco.org/may-2023-quarterly-bulletin-of-cocoa-statistics/.

[ref33] Picchioni F.; Warren G. P.; Lambert S.; Balcombe K.; Robinson J. S.; Srinivasan C.; Gomez L. D.; Faas L.; Westwood N. J.; Chatzifragkou A.; Charalampopoulos D.; Shaw L. J. Valorisation of Natural Resources and the Need for Economic and Sustainability Assessment: The Case of Cocoa Pod Husk in Indonesia. Sustainability 2020, 12, 8962 10.3390/su12218962.

[ref34] Campos-Vega R.; Nieto-Figueroa K. H.; Oomah B. D. Cocoa (*Theobroma cacao* L.) pod husk: Renewable source of bioactive compounds. Trends in Food Science & Technology 2018, 81, 172–184. 10.1016/j.tifs.2018.09.022.

[ref35] Lu F.; Rodriguez-Garcia J.; van Damme I.; Westwood N. J.; Shaw L.; Robinson J. S.; Warren G.; Chatzifragkou A.; McQueen Mason S.; Gomez L.; Faas L.; Balcombe K.; Srinivasan C.; Picchioni F.; Hadley P.; Charalampopoulos D. Valorisation Strategies for Cocoa Pod Husk and Its Fractions. Current Opinion in Green and Sustainable Chemistry 2018, 14, 80–88. 10.1016/j.cogsc.2018.07.007.

[ref36] Hozman-Manrique A. S.; Garcia-Brand A. J.; Hernández-Carrión M.; Porras A. Isolation and Characterization of Cellulose Microfibers from Colombian Cocoa Pod Husk via Chemical Treatment with Pressure Effects. Polymers 2023, 15, 664 10.3390/polym15030664. 36771964 PMC9919290

[ref37] Muharja M.; Darmayanti R. F.; Fachri B. A.; Palupi B.; Rahmawati I.; Rizkiana M. F.; Amini H. W.; Putri D. K. Y.; Setiawan F. A.; Asrofi M.; Widjaja A.; Halim A. Biobutanol production from cocoa pod husk through a sequential green method: Depectination, delignification, enzymatic hydrolysis, and extractive fermentation. Bioresource Technology Reports 2023, 21, 101298 10.1016/j.biteb.2022.101298.

[ref38] Huamani-Palomino R. G.; Ramos M P.; Oliveira G.; Kock F. V. C.; Venâncio T.; Córdova B. M. Structural elucidation of pectin extracted from cocoa pod husk (*Theobroma Cacao L.*): Evaluation of the degree of esterifcation using FT-IR and 1 H NMR. Biomass Conversion and Biorefinery 2023, na 10.1007/s13399-023-04082-3.

[ref39] Hennessey-Ramos L.; Murillo-Arango W.; Vasco-Correa J.; Paz Astudillo I. C. Enzymatic Extraction and Characterization of Pectin from Cocoa Pod Husks (*Theobroma cacao* L.) Using Celluclast® 1.5 L. Molecules 2021, 26, 1473 10.3390/molecules26051473. 33803082 PMC7963153

[ref40] Priyangini F.; Walde S. G.; Chidambaram R. Extraction Optimization of Pectin from Cocoa Pod Husks (*Theobroma Cacao L.*) with Ascorbic Acid Using Response Surface Methodology. Carbohydr. Polym. 2018, 202, 497–503. 10.1016/j.carbpol.2018.08.103. 30287028

[ref41] Adomako D. Cocoa Pod Husk Pectin. Phytochemistry 1972, 11, 1145–1148. 10.1016/S0031-9422(00)88468-X.

[ref42] Hutomo G. S.; Rahim A.; Kadir S. Pectin Isolation from Dry Pod Husk Cocoa with Hydrochloride Acid. International Journal of Current Microbiology and Applied Sciences 2016, 5 (11), 751–756. 10.20546/ijcmas.2016.511.086.

[ref43] Mollea C.; Chiampo F.; Conti R. Extraction and Characterization of Pectins from Cocoa Husks: A Preliminary Study. Food Chem. 2007, 107 (3), 1353–1356. 10.1016/j.foodchem.2007.09.006.

[ref44] Vriesmann L. C.; de Mello Castanho Amboni R. D.; de Oliveira Petkowicz C. L. Cacao Pod Husks (*Theobroma Cacao L.*): Composition and Hot-Water-Soluble Pectins. Ind. Crop Prod. 2011, 34 (1), 1173–1181. 10.1016/j.indcrop.2011.04.004.

[ref45] Nazir N.; Novelina; Juita E.; Amelia C.; Fatli R. Optimization of Pre-Treatment Process of Cocoa Pod Husk Using Various Chemical Solvents. International Journal on Advanced Science. Engineering and Information Technology 2016, 6 (3), 403–409. 10.18517/ijaseit.6.3.848.

[ref46] Mashuni; Hamid F. H.; Muzuni; Kadidae L. O.; Jahiding M.; Ahmad L. O.; Saputra D. The determination of total phenolic content of cocoa pod husk based on microwave-assisted extraction method. AIP Conf. Proc. 2020, 2243, 030013 10.1063/5.0001364.

[ref47] Lancefield C. S.; Panovic I.; Deuss P. J.; Barta K.; Westwood N. J. Pre-Treatment of Lignocellulosic Feedstocks Using Biorenewable Alcohols: Towards Complete Biomass Valorisation. Green Chem. 2017, 19 (1), 202–214. 10.1039/C6GC02739C.

[ref48] Ralph J.; Landucci L. L. NMR of Lignins. In Lignin and Lignans; Advances in Chemistry; Heitner C., Dimmel D. R., Schmidt J. A., Eds.; CRC Press (Taylor & Francis Group): Boca Raton, FL, 2010; pp 138–234.

[ref49] Giummarella N.; Pylypchuk I. v.; Sevastyanova O.; Lawoko M. New Structures in Eucalyptus Kraft Lignin with Complex Mechanistic Implications. ACS Sustainable Chem. Eng. 2020, 8 (29), 10983–10994. 10.1021/acssuschemeng.0c03776.

[ref50] Willker W.; Leibfritz D. Assignment of Mono- and Polyunsaturated Fatty Acids in Lipids of Tissues and Body Fluids. Magn. Reson. Chem. 1998, 36 (S1), S79–S84. 10.1002/(SICI)1097-458X(199806)36:13<S79::AID-OMR294>3.0.CO;2-Z.

[ref51] Rachmawaty; Mu’nisa A.; Hasri; Pagarra H.; Hartati; Maulana Z. Active Compounds Extraction of Cocoa Pod Husk (*Thebroma Cacao l.*) and Potential as Fungicides. Journal of Physics: Conference Series 2018, 1028 (1), 012013 10.1088/1742-6596/1028/1/012013.

[ref52] Zijlstra D. S.; de Korte J.; de Vries E. P. C.; Hameleers L.; Wilbers E.; Jurak E.; Deuss P. J. Highly Efficient Semi-Continuous Extraction and In-Line Purification of High β-O-4 Butanosolv Lignin. Frontiers in Chemistry 2021, 9, 329 10.3389/fchem.2021.655983. PMC8141753 34041222

[ref53] Montgomery J. R. D.; Lancefield C. S.; Miles-Barrett D. M.; Ackermann K.; Bode B. E.; Westwood N. J.; Lebl T. Fractionation and DOSY NMR as Analytical Tools: From Model Polymers to a Technical Lignin. ACS Omega 2017, 2 (11), 8466–8474. 10.1021/acsomega.7b01287. 31457383 PMC6645228

[ref54] Ralph J.; Hatfield R. D.; Quideau S.; Helm R. F.; Grabber J. H.; Jung H. J. G. Pathway of P-Coumaric Acid Incorporation into Maize Lignin As Revealed by NMR. J. Am. Chem. Soc. 1994, 116 (21), 9448–9456. 10.1021/ja00100a006.

[ref55] Wu Y.; Huang Z.; Lv K.; Rao Y.; Chen Z.; Zhang J.; Long J. Producing Methyl P-Coumarate from Herbaceous Lignin via a “Clip-Off” Strategy. J. Agric. Food Chem. 2022, 70, 5624–5633. 10.1021/acs.jafc.1c08353. 35473308

[ref56] Hilgers R.; Vincken J. P.; Kabel M. A. Facile Enzymatic Cγ-Acylation of Lignin Model Compounds. Catal. Commun. 2020, 136, 105919 10.1016/j.catcom.2019.105919.

[ref57] Kim H.; Padmakshan D.; Li Y.; Rencoret J.; Hatfield R. D.; Ralph J. Characterization and Elimination of Undesirable Protein Residues in Plant Cell Wall Materials for Enhancing Lignin Analysis by Solution-State Nuclear Magnetic Resonance Spectroscopy. Biomacromolecules 2017, 18 (12), 4184–4195. 10.1021/acs.biomac.7b01223. 29064677

[ref58] Kumaniaev I.; Navare K.; Mendes M. C.; Placet V.; Van Acker K.; Samec J. S. M. Conversion of birch bark to biofuels. Green Chem. 2020, 22, 2255–2263. 10.1039/D0GC00405G.

[ref59] Hong S.; Shen X.-J.; Pang B.; Xue Z.; Cao X.-F.; Wen J.-L.; Sun Z.-H.; Lam S. S.; Yuan T.-Q.; Sun R.-C. In-depth interpretation of the structural changes of lignin and formation of diketones during acidic deep eutectic solvent pretreatment. Green Chem. 2020, 22, 1851–1858. 10.1039/D0GC00006J.

[ref60] Panovic I.; Montgomery J. R. D.; Lancefield C. S.; Puri D.; Lebl T.; Westwood N. J. Grafting of Technical Lignins through Regioselective Triazole Formation on β-O-4 Linkages. ACS Sustain. Chem. Eng. 2017, 5 (11), 10640–10648. 10.1021/acssuschemeng.7b02575.

[ref61] Panovic I.; Miles-Barrett D. M.; Lancefield C. S.; Westwood N. J. Preparation and Reaction of β-O-4 γ-Aldehyde-Containing Butanosolv Lignins. ACS Sustain. Chem. Eng. 2019, 7 (14), 12098–12104. 10.1021/acssuschemeng.9b01188.

[ref62] Gioia C.; Colonna M.; Tagami A.; Medina L.; Sevastyanova O.; Berglund L. A.; Lawoko M. Lignin-Based Epoxy Resins: Unravelling the Relationship between Structure and Material Properties. Biomacromolecules 2020, 21 (5), 1920–1928. 10.1021/acs.biomac.0c00057. 32160463 PMC7997103

[ref63] Ribca I.; Sochor B.; Betker M.; Roth S. V.; Lawoko M.; Sevastyanova O.; Meier M. A. R.; Johansson M. Impact of lignin source on the performance of thermoset resins. Eur. Polym. J. 2023, 194, 112141 10.1016/j.eurpolymj.2023.112141.

[ref64] Wang X.; Hu Y.; Song L.; Yang H.; Xing W.; Lu H. Synthesis and Characterization of a DOPO-Substitued Organophosphorus Oligomer and Its Application in Flame Retardant Epoxy Resins. Prog. Org. Coat. 2011, 71 (1), 72–82. 10.1016/j.porgcoat.2010.12.013.

[ref65] Kawamoto H. Lignin Pyrolysis Reactions. Journal of Wood Science 2017, 63 (2), 117–132. 10.1007/s10086-016-1606-z.

[ref66] Fan Y.; Lei M.; Zhang Z.; Kong X.; Xu W.; Han Y.; Li M.; Liu C.; Xiao R. Unmasking Radical-Mediated Lignin Pyrolysis after Benzyl Hydroxyl Shielding. Bioresour. Technol. 2021, 342, 125944 10.1016/j.biortech.2021.125944. 34537528

[ref67] Gong J.; Li J.; Xu J.; Xiang Z.; Mo L. Research on Cellulose Nanocrystals Produced from Cellulose Sources with Various Polymorphs. RSC Adv. 2017, 7 (53), 33486–33493. 10.1039/C7RA06222B.

[ref68] Lu F.; Ralph J. Derivatization Followed by Reductive Cleavage (DFRC Method), a New Method for Lignin Analysis: Protocol for Analysis of DFRC Monomers. J. Agric. Food Chem. 1997, 45 (7), 2590–2592. 10.1021/jf970258h.

[ref69] Monsoor M. A.; Kalapathy U.; Proctor A. Improved Method for Determination of Pectin Degree of Esterification by Diffuse Reflectance Fourier Transform Infrared Spectroscopy. J. Agric. Food Chem. 2001, 49 (6), 2756–2760. 10.1021/jf0009448. 11409962

[ref70] Zhu X.; Liu B.; Zheng S.; Gao Y. Quantitative and Structure Analysis of Pectin in Tobacco by ^13^C CP/MAS NMR Spectroscopy. Anal. Methods 2014, 6, 6407–6413. 10.1039/C4AY01156B.

[ref71] Montgomery J. R. D.; Bazley P.; Lebl T.; Westwood N. J. Using Fractionation and Diffusion Ordered Spectroscopy to Study Lignin Molecular Weight. ChemistryOpen 2019, 8 (5), 601–605. 10.1002/open.201900129. 31110931 PMC6511914

[ref72] Duval A.; Vilaplana F.; Crestini C.; Lawoko M. Solvent screening for the fractionation of industrial kraft lignin. Holzforschung 2016, 70 (1), 11–20. 10.1515/hf-2014-0346.

[ref73] Mansur D.; Tago T.; Masuda T.; Abimanyu H. Conversion of cacao pod husks by pyrolysis and catalytic reaction to produce useful chemicals. Biomass and Bioenergy 2014, 66, 275–285. 10.1016/j.biombioe.2014.03.065.

[ref74] Alemawor F.; Dzogbefia V. P.; Oddoye E. O. K.; Oldham J. H. Enzyme cocktail for enhancing poultry utilisation of cocoa pod husk. Scientific Research and Essay 2009, 4 (6), 555–559. 10.5897/SRE.9000454.

