# Supplementary material for: Organosolv Pretreatment of Cocoa Pod Husks: Isolation, Analysis, and Use of Lignin from an Abundant Waste Product
Source: ACS Sustain Chem Eng. 2023 Sep 21;11(39):14323–33. doi: 10.1021/acssuschemeng.2c03670 (PMC10548466; doi:10.1021/acssuschemeng.2c03670)
Supplement: Supplementary file 1 — sc2c03670_si_001.pdf [file sc2c03670_si_001.pdf]

Supporting Information

Organosolv Pretreatment of Cocoa Pod Husks: Isolation, Analysis and Use of Lignin from an Abundant Waste Product

Daniel J. Davidson,[a] Fei Lu,[b] Laura Faas,[c] Daniel M. Dawson,[a] Geoffrey P. Warren,[d] Isabella Panovic,[a] James R.D. Montgomery,[a] Xiaoyan Ma,[a] Boris G. Bosilkov,[a] Alexandra M.Z. Slawin,[a] Tomas Lebl,[a] Afroditi Chatzifragkou,[b] Steve Robinson,[d] Sharon E. Ashbrook,[a] Liz J. Shaw,[d] Smilja Lambert,[e] Isabella Van Damme[f] Leonardo D. Gomez,[c] Dimitris Charalampopoulos,[b] Nicholas J. Westwood[a]*

[a]School of Chemistry and Biomedical Sciences Research Complex, University of St Andrews and EaStCHEM, North Haugh, St Andrews, Fife KY16 9ST (UK).

[b]Department of Food and Nutritional Sciences, University of Reading,UK

[c]Centre for Novel Agricultural Products, Department of Biology, University of York, York YO10 5DD, UK

[d]Soil Research Centre, Department of Geography and Environmental Science, University of Reading, Reading RG6 6DW, UK

[e]Mars Wrigley Australia, Ring Road, Wendouree, VIC 3355, Australia

[f]Mars Wrigley Confectionery UK Ltd, 3D Dundee Road, Slough, Berkshire, SL1 4LG, UK

Number of pages: 56

Number of figures: 61

Number of schemes: 3

Number of tables: 6

Contents

General considerations.................... S3

Cocoa Pod Husk (CPH) pretreatment general procedures S4-S5

Butanosolv lignin modification general procedures.................... S6

Alternative pretreatments.................... S7-S9

CPH lignin Analysis.................... S10-S13

NMR Comparison Analysis of CPH Lignin and Model S14-S17

CPH Lignin Modification and TGA.................... S18

Analysis of other CPH fractions S19-S26

Model compound synthesis S27-S34

Model compound ^{1}H, ^{13}C and ^{31}P NMR spectra.................... S35-S45

Lignin HSQC and ^{31}P NMR spectra S46-S55

References.................... S56

General Considerations

Commercially available compounds were purchased and used as received unless otherwise stated. Purified tosyl chloride was prepared as follows: commercially available tosyl chloride was dissolved in chloroform (2.5 mL/g) and diluted with hexane (12.5 mL/g). Activated charcoal was added and after stirring the suspension filtered through a Celite pad. The filtrate was concentrated under vacuum and dried under vacuum at 50 °C for 24 hours to afford purified tosyl chloride. Cocoa pod husk (CPH) biomass was received as three different clone batches that had been freeze dried and were frozen upon arrival. The individual clone batches were defrosted just prior to milling and were milled using a Retsch SM 300 SM mill equipped with a 1 mm screen. The batches were combined and thoroughly mixed to give a homogenous CPH biomass. The CPH was stored frozen and defrosted just prior to use in the pretreatment protocol.

Solution-state NMR spectra were obtained using Bruker AV-II 400, Bruker AV-III 500 fitted with CryoProbe Prodigy BBO, and Bruker AV-III-HD 700 fitted with CryProbe Prodigy TCI. NMR data were processed using MestReNova v12.0.3, TopSpin v4.1.1 and Dynamics Center 2.7.2. Further processing of spectra for figures was carried out using Adobe Illustrator 2022 v26.3.1.

Solid-state NMR spectra were recorded using Bruker Advance III spectrometers equipped with wide-bore superconducting 14.1 and 9.4 T magnets. Samples were packed into standard 4 mm rotors and rotated about the magic angle at a rate of 12.5 kHz. Spectra were recorded with cross polarisation from 1H with a contact pulse (ramped for 1H) of between 0.5 and 5 ms. High-power (n1 ≈ 100 kHz) TPPM-15 decoupling was applied during acquisition. Signal averaging was carried out for between 3200 and 4096 transients with a recycle interval of 3 s. Chemical shifts are reported in ppm relative to TMS using the CH_3 signal of L-alanine (δ = 20.5 ppm) as a secondary solid reference. Spectral decomposition and integration was performed using DMFit.

IR spectra were obtained using a Shimadzu IRAffinity 1S IR Spectrometer as ATR. The IR data were processed using OriginPro 2022 v9.9.0.225.

Mass spectrometry data were acquired through the University of St Andrews School of Chemistry mass spectrometry service.

Powder X-ray diffraction (PXRD) patterns were obtained using PANalytical Empyrean fitted with a Cu X-ray tube, a primary beam monochromator (CuKa1) and a X'celerator RTMS detector. PXRD data were processed using OriginPro 2022 v9.9.0.225.

TGA data were obtained using a Fdata wStanton Redcroft STA-780 simultaneous TG-DTA. All samples were dried under vacuum at 50 °C for 24 hours prior to TGA. Approximately 8 mg of sample was weighed into the crucible. Measurements were performed under nitrogen flow (22 mL/min) at a heating rate of 10 °C/min. TGA/DTG data were processed using OriginPro 2022 v9.9.0.225.

Cocoa Pod Husks (CPH) Pretreatment

Lignin extraction Protocols

Alkali Fractionation (see Figure S1)**:** CPH was fractionated under alkaline conditions according to previous protocols with some modifications.[S1,S2] Briefly, 5 g of dry-milled sample of CPH was mixed with 200 mL aqueous NaOH solution (0.75, 1.0 and 1.5 M) at 50°C, in a 1:20 solid to liquid ratio. The mixture was incubated at 50 °C and stirred at 200 rpm for 3 hours, followed by centrifugation at 17,105 x g for 20 minutes. The collected residue was washed with deionised water and freeze-dried (Virtis SP scientific model 2KBTES, USA) to give a fraction designated as the cellulose rich fraction. The alkali-soluble supernatant was adjusted to pH 5.5 using 6 M HCl and concentrated to about one-third of its original volume in a rotary evaporator. Then, three volumes of ethanol (95%, v/v) were slowly poured into the solution under constant stirring. The precipitated solid was separated and washed with 95% ethanol using a filter paper. This solid was designated as the alkali-soluble hemicellulose fraction. The ethanol solution was designated as the soluble lignin fraction and was collected as a solid after evaporating the ethanol under vacuum.

Steam explosion: (see Figure S2)**:** Initially, the pectin was removed from the CPH by mixing the CPH material (milled to 1mm particle size) with deionised water in a 1:30 solid/liquid ratio and incubating the mixture for 4h at 80°C, with stirring (180 rpm). The washed solids were recovered by centrifugation (Beckman Avanti J265 centrifuge, 10 min at 15,900 g). For the steam explosion pretreatment, the solid loading was adjusted to 15% by the addition of water. The material was pretreated in a 2L Parr vessel at 140 °C and 50 rpm for 1 h, followed by rapid release of the pressure *(i.e.* steam explosion).

Ethanosolv Pretreatment: CPH was suspended in EtOH/4M HCl (95:5, 10 mL/g) and heated at reflux for 6 hours. The mixture was allowed to cool and filtered at room temperature. The solid residue was washed with acetone (5 x 10 mL/g). The filtrate was concentrated under reduced pressure, dissolved in the minimum volume of acetone/water (9:1) and added slowly to ice-cold water (10 v/v eq.). The resulting suspension was then filtered and the residue washed with cold water (3 x 15 mL/g). The residue was then dissolved in the minimum volume of acetone/methanol (9:1) and added slowly to hexane/ether (1:1, 10 v/v eq.), before filtration and washing of the solid with hexane (5 x 10 mL/g). The residue was dried under vacuum at 60 °C for 24 h to afford EthanoSolv Lignin.

Butanosolv Pretreatment:

Literature Butanosolv pretreatment protocol: Following a literature procedure used with other biomass samples,[S3] CPH was suspended in *n*-butanol/4M HCl (95:5, 10 mL/g) and heated at reflux for 6 hours. The mixture was allowed to cool and filtered at room temperature, washing the solid residue with acetone (5 x 10 mL/g). The residue was dried under vacuum at 60 °C for 24 h to afford the cellulose pulp fraction. The filtrate was concentrated under reduced pressure, dissolved in the minimum volume of acetone/water (9:1) and added slowly to ice-cold water (10 v/v eq.), using solid Na_2SO_4 as a flocculant. The resulting solid was isolated by filtration and washed with cold water (3 x 15 mL/g). The filtrate was concentrated under reduced pressure and freeze dried to afford the **hemi**cellulose-derived fraction. The residue was dissolved in the minimum volume of acetone/methanol (9:1) and added slowly to hexane/ether (1:1, 10 v/v eq.). The resulting suspension was then filtered, and the residue washed with hexane (5 x 10 mL/g). The filtrate was concentrated under reduced pressure and dried under vacuum at 60 °C for 24 h to afford the **O**rganic **S**oluble **E**xtractives (**OSE**) fraction. The residue was dried under vacuum at 60 °C for 24 hours to afford the lignin fraction.

Butanosolv pretreatment with ethanol pre-wash: CPH was suspended in EtOH (10 mL/g) and stirred at room temperature for 18 h. The mixture was then filtered and the recovered CPH pulp was suspended in fresh EtOH (10 mL/g) and stirred for 4 hours. The mixture was filtered and the filtrates combined, concentrated *in vacuo* and then dried under vacuum at 60 °C for 24 hours to afford a triglyceride and Fatty Acid-containing fraction. The CPH pulp was dried under vacuum at 60 °C for 24 hours then treated as in the original butanosolv pretreatment described above.

Butanosolv pretreatment with aqueous extraction: CPH was suspended in water (25 mL/g) and heated at reflux for 4 hours. The mixture was cooled to room temperature, centrifuged at 5500 rpm at 4 °C for 1 hour, strained through cheesecloth, washed with fresh water (3 x 10 mL/g) and squeezed until dry. The CPH pulp was dried under vacuum at 60 °C for 24 hours then treated as in the original butanosolv pretreatment described above. The filtrate was precipitated into 2 V/V eq. of ice cold EtOH, centrifuged at 5500 rpm at 4 °C for 1 h and strained through cheesecloth. The filtrate was concentrated under reduced pressure and freeze dried to afford the Pectin **E**thanol **S**oluble **F**raction (**PESF**). The crude pectin gel residue was dried under vacuum at 60 °C for 24 h then ground into a fine powder. The powder was suspended in MeOH (5 mL/g) and treated with ultrasound for 5 minutes. at room temperature and then filtered. This was repeated twice more and then three times using EtOH (5 mL/g). The filtrates were combined with the **PESF**. The powder was dried under vacuum at 60 °C for 24 hours to afford the pectin fraction.

Optimised butanoslv pretreatment protocol: CPH was pre-washed with EtOH, then an aqueous extraction was carried out and finally the original butanosolv pretreatment was used as described above. See Materials and Methods section in manuscript for full experimental.

Dioxasolv Pretreatment: CPH was suspended in 1,4-dioxane/2M HCl (9:1, 8 mL/g) and heated at reflux for 2 hours. The mixture was cooled and filtered at room temperature, washing the solid residue with acetone (5 x 10 mL/g). The filtrate was concentrated under reduced pressure, dissolved in the minimum volume of acetone/water (9:1) and added slowly to ice-cold water (10 v/v eq.), then filtered. The residue was washed with cold water (3 x 15 mL/g), dissolved in the minimum volume of acetone/methanol (9:1) and added slowly to hexane/ether (1:1, 10 v/v eq.), then filtered with the resulting solid being washed with hexane (3 x 10 mL/g). The solid was dried under vacuum at 60 °C for 24 hours to afford DioxaSolv Lignin.

Caustic Soda Treatment: Adapted from a literature procedure,[S4] Butanosolv lignin (0.50 g) was suspended in sodium hydroxide solution (10 mL/g, 0.1 M) and heated at 50 °C for 24 hours. The suspension was neutralised with sat. aq. NH_4Cl solution and the resulting precipitate washed with water (5 x 15 mL/g) then dried under vacuum at 60 °C for 24 hours to afford **C**austic **S**oda **T**reatment (**CST**)-processed butanosolv lignin.

Butanosolv Lignin Modification General Procedures:

Step 1 - Tosylation: Based on a literature procedure,[S5] CPH lignin (1 wt eq.) and tosyl chloride (3 wt eq.) were stirred in pyridine (10 mL/g of lignin) at room temperature for 24 hours. The reaction was then added dropwise to 0.1 M HCl (10 v/v eq.) and the resulting precipitate isolated by filtration under vacuum, washed with water (30 mL/g) and dried under vacuum at 60 °C for 2 hours. The crude lignin was redissolved in the minimum volume of acetone and was then added dropwise to cold EtOH (10 v/v eq.). The resulting precipitate was isolated by filtration under vacuum, washed with cold EtOH (30 mL/g) and dried under vacuum at 60 °C for 24 hours to afford Lignin-Ts.

Step 2 - Azidation: Based on a literature procedure,[S5] Lignin-Ts (1 wt eq.) and sodium azide (2 wt eq.) were stirred in DMF (10 mL/g Lignin-Ts) at 50 °C for 24 hours. The resulting suspension was added dropwise to 0.1 M HCl (10 v/v eq.) and the reaction filtered. The residue was washed with water (30 mL/g) and dried under vacuum at 50 °C for 24 hours to afford Lignin-N_3.

Step 3 - CuAAC Click reaction: Based on a literature procedure,[S5] Lignin-N_3 (1 wt eq.), DOPO alkyne derivative **10** or alternative alkynes (0.5 wt eq.), sodium ascorbate (0.5 wt eq.) and $CuSO_4.5H_2O$ (0.03 wt eq.) were stirred in DMF/water (5:1, 10 mL/g Lignin-N_3) at room temperature for 24 hours. The solution was added dropwise to 0.1 M HCl (10 v/v eq.) and the resulting precipitate isolated by filtration, washed with water (30 mL/g) and dried under vacuum at 60 °C for 24 hours. The crude lignin was purified by column chromatography on silica gel (30 g/g) eluting with DCM/hexane (0-100%), MeOH/DCM (0-10%) then 100% acetone to afford Lignin-DOPO.

Lignin NMR Sample Preparation: Lignin (60.0 ± 0.1 mg) was dissolved in DMSO-d_6 (0.70 mL) and treated with ultrasound for 15 minutes at room temperature to ensure complete dissolution.

Alternative Pretreatments

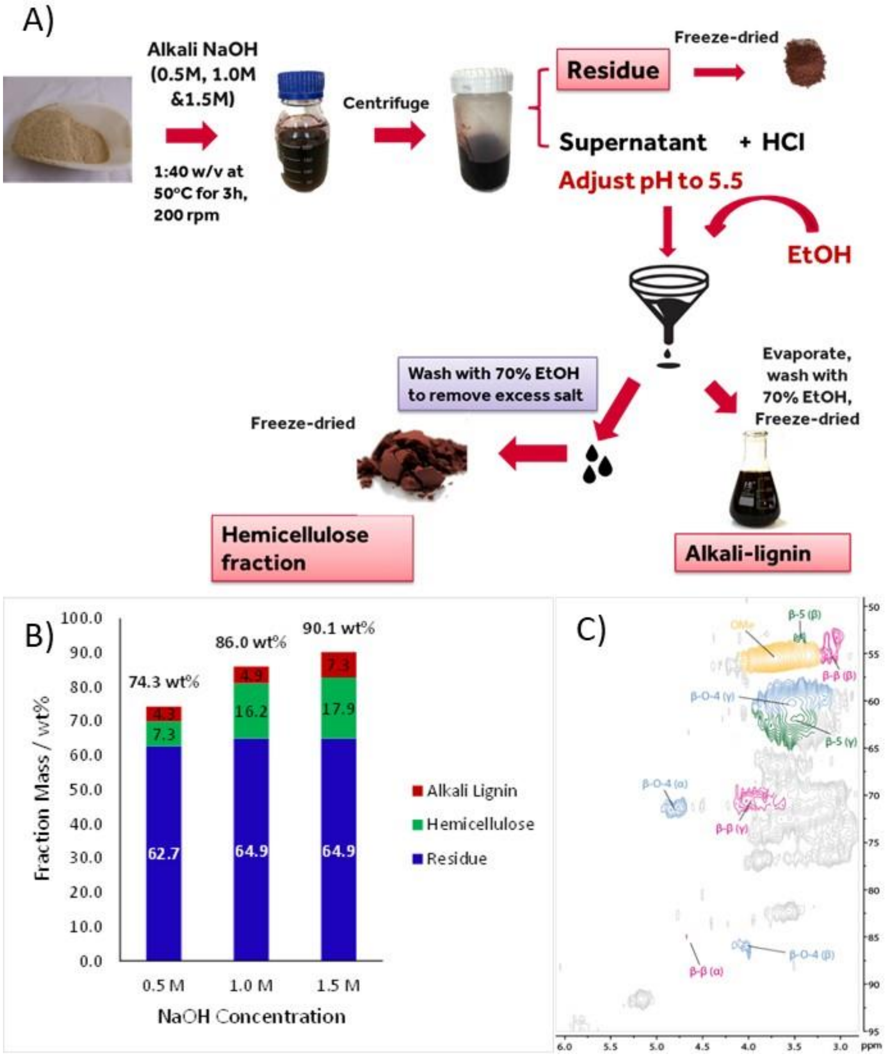

Figure S1: Summary of the outcomes from the alkali pretreatment applied to cocoa pod husks: See General Considerations section above for detailed description of the Experimental Protocol. A) overview of pretreatment protocol and the formation of 3 product streams entitled (1) residue, (2) hemicellulose fraction and (3) alkali lignin; B) Mass balance as a function of the concentration of aqueous sodium hydroxide solution used in the pretreatment; C) 2D HSQC NMR analysis of the alkali lignin isolated when 1.5M aqueous sodium hydroxide solution was used. Whilst signals corresponding to the β-O-4 units were present, their relative abundance and the significant background noise suggested that this lignin was of lower quality.

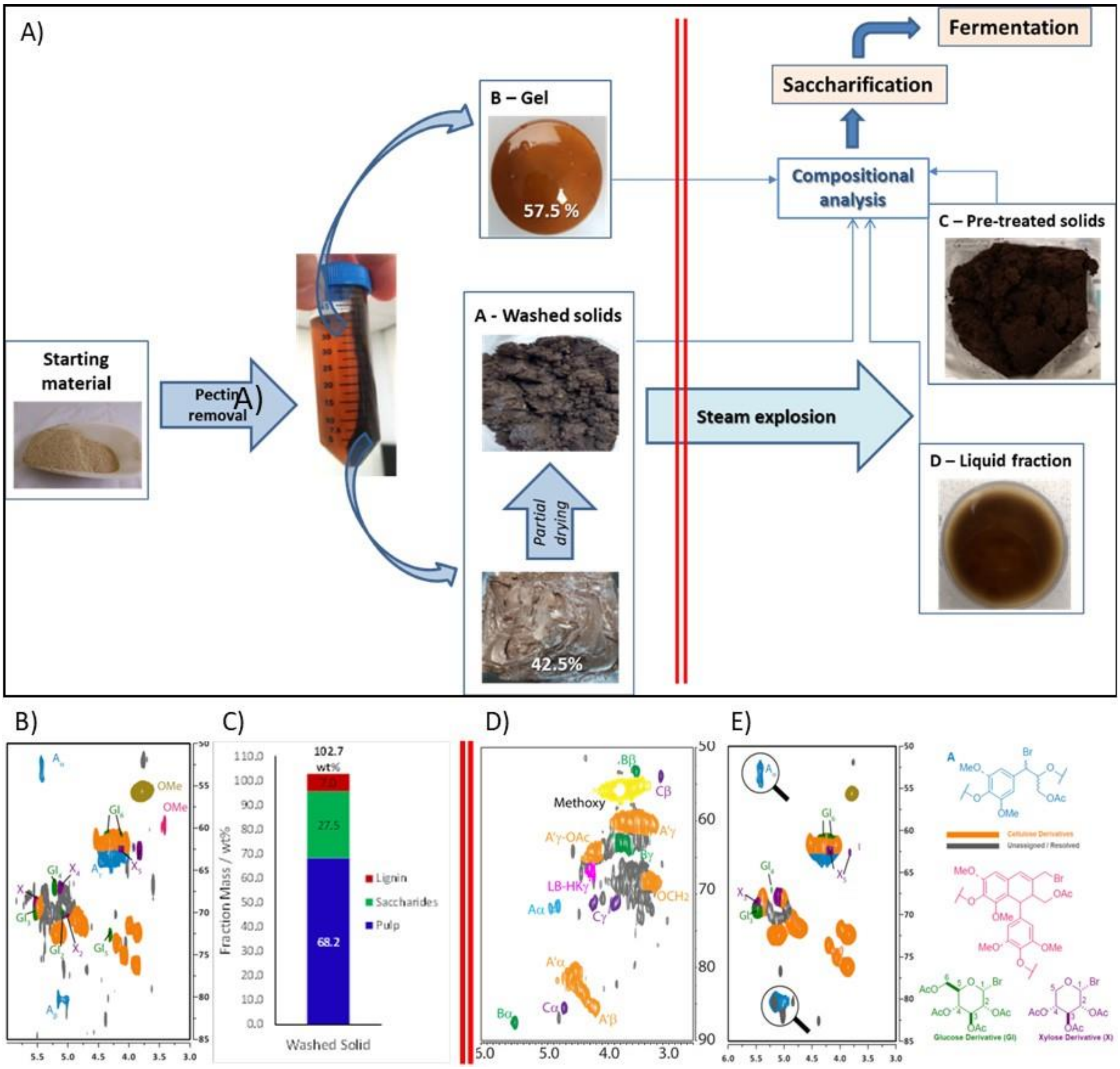

Figure S2: Summary of the outcomes from the steam explosion pretreatment applied to cocoa pod husks: See General Considerations section above for detailed description of the Experimental Protocol. A) overview of pretreatment protocol highlighting 2 fractions (gel and washed solids) produced prior to the steam explosion and 2 fractions (pre-treated solids and liquid fraction) produced after the steam explosion of the washed solids; B) AcBr reaction and 2D HSQC NMR analysis of the washed solids indicating the presence of both cellulose and lignin in this fraction. Colour-coded structures are provided to aid interpretation; C) Composition of washed solids as judged by carrying out a butanosolv pretreatment on the washed solids; D) 2D HSQC NMR of the pre-treated solids after steam explosion followed by butanosolv pretreatment according to a literature protocol[S3]; E) AcBr reaction and 2D HSQC NMR analysis of the concentrated liquid fraction emphasising the enrichment of this fraction by cellulose compared to the washed solids (*c.f.* insert B). Only trace quantities of lignin were present in this fraction (magnifying glass signifies that a much lower threshold level was used when analysing the NMR spectrum to generate these inserts).

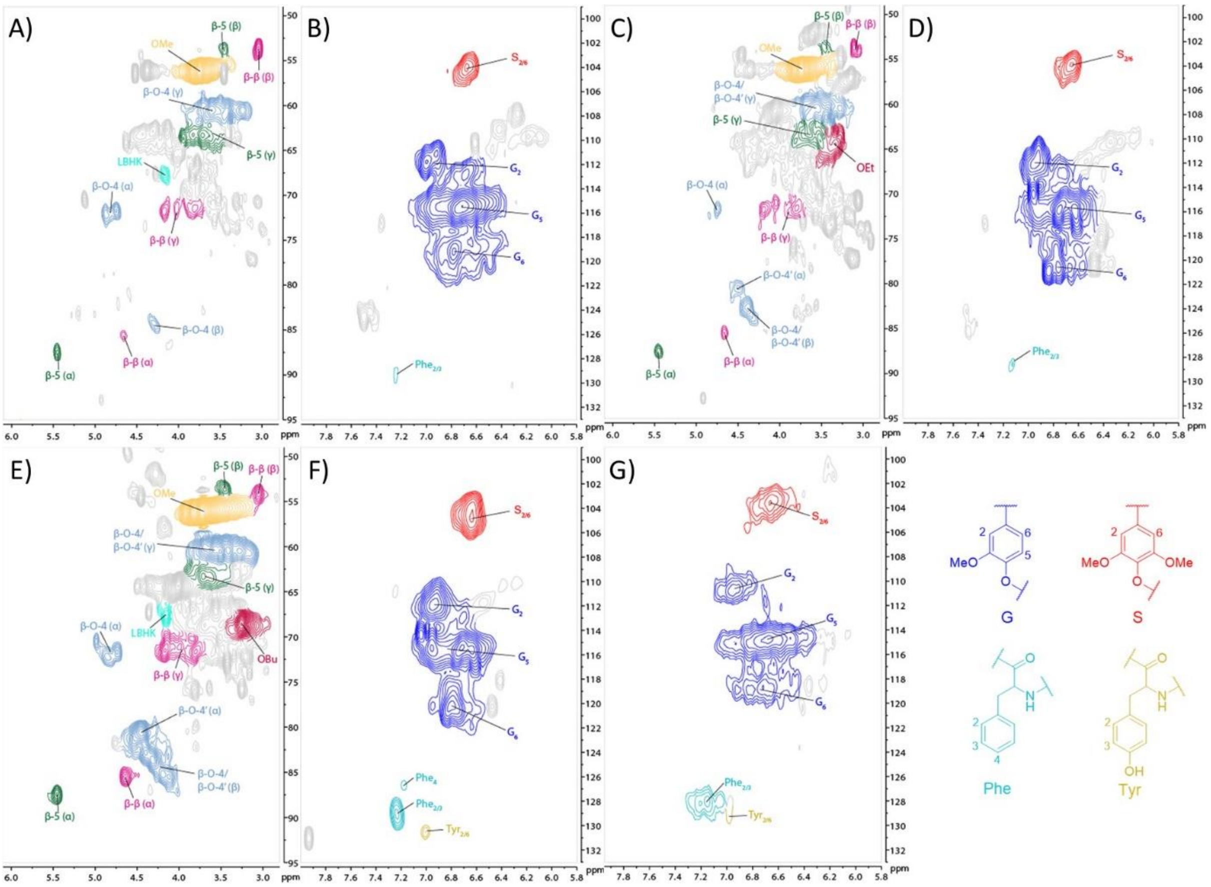

Figure S3: HSQC NMR (700 MHz, DMSO-d_6) analysis of some of the lignins isolated in the preliminary stages of this project. A) The linkage region and B) aromatic region of CPH dioxasolv lignin; C) linkage region and D) aromatic region of CPH ethanosolv lignin; E) linkage region and F) aromatic region of CPH butanosolv lignin (isolated using literature protocol[S3]); G) aromatic region of alkali lignin (*c.f.* Figure S1 for image of linkage region). Relevant aromatic structures are shown. The signals labelled Phe$_{2/3}$, Phe$_4$ and Tyr$_{2/3}$ were assigned based on a previous literature report.[S6]

CPH lignin Analysis

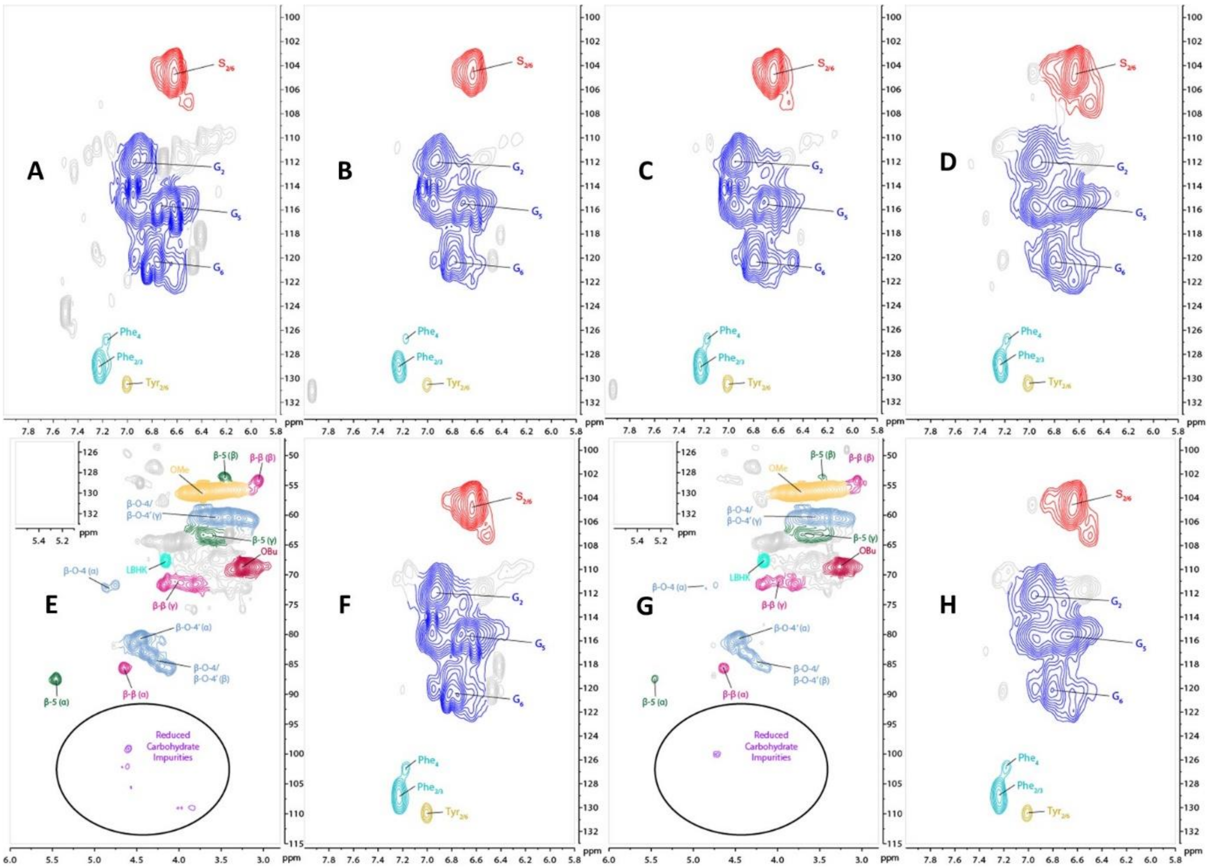

Figure S4: HSQC NMR (700 MHz, DMSO-d_6) analysis of the aromatic region of the butanosolv lignin obtained using a literature protocol[S3]: A) before the final purification by hexane/diethyl ether precipitation; B) after hexane/diethyl ether precipitation; C) after an alternative purification method using a caustic soda treatment[S4]; D) the butanosolv lignin obtained using the optimised pretreatment developed in this study. Entries A-D are the aromatic regions corresponding to the linkage region spectra presented in manuscript Figure 2. Linkage region of butanosolv lignin isolated following: E) an ethanol pre-wash and butanosolv pretreatment (see General Considerations Section of SI for all protocol details); G) a hot aq. Extraction and butanosolv pretreatment. Aromatic region of butanosolv lignin isolated following: F) an ethanol pre-wash and butanosolv pretreatment; H) a hot aq. Extraction and butanosolv pretreatment. Relevant linkage structures are shown in manuscript Figure 2 and relevant aromatic structures are shown in Figure S3.

Pretreatment Protocol	Sugar contamination
Literature protocol[S3] (before final purification)	60
Literature protocol[S3] (after final purification)	46
Literature protocol[S3] (after CST purification)	34
Ethanol pre-wash and literature protocol[S3]	35
Hot aqueous extraction and literature butanosolv[S3]	33
Optimised pretreatment	28
Filtrate from final purification in the optimised pretreatment	92

Table S1: Relative carbohydrate contamination levels calculated using Equation S1 applied to the corresponding 2D HSQC NMR analysis. It is assumed that these signals correspond to the anomeric protons of monosaccharides formed through depolymerisation of carbohydrate-based polymers such as hemicelluloses and that therefore use of signal integration in a 2D HSQC spectrum is reasonable. These studies were inspired by a report by Deuss and Jurak *et. al*.[S4]

Equation S1: $$Carbohydrate\ Impuritiy\ Level = \frac{1}{G\ aromatics + S\ aromatics}\ x\ 100$$

Where the integration corresponding to the relevant carbohydrate signals in the HSQC analysis has been set to 1.

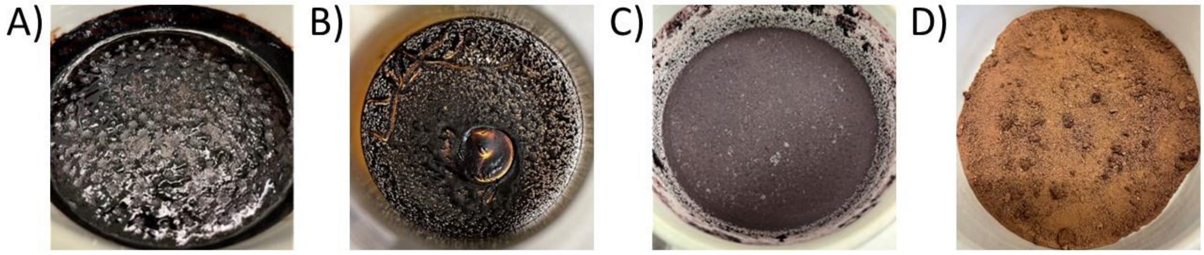

Figure S5: A) CPH butanosolv lignin residue from initial literature protocol[S3] before the final purification step. It proved impossible to obtain a granular precipitate; B) CPH butanosolv lignin residue from initial literature protocol[S3] after purification; C) CPH butanosolv lignin residue from the optimised pretreatment before the final purification step; D) CPH butanosolv lignin residue from the optimised pretreatment after purification. A free flowing granular precipitate was formed.

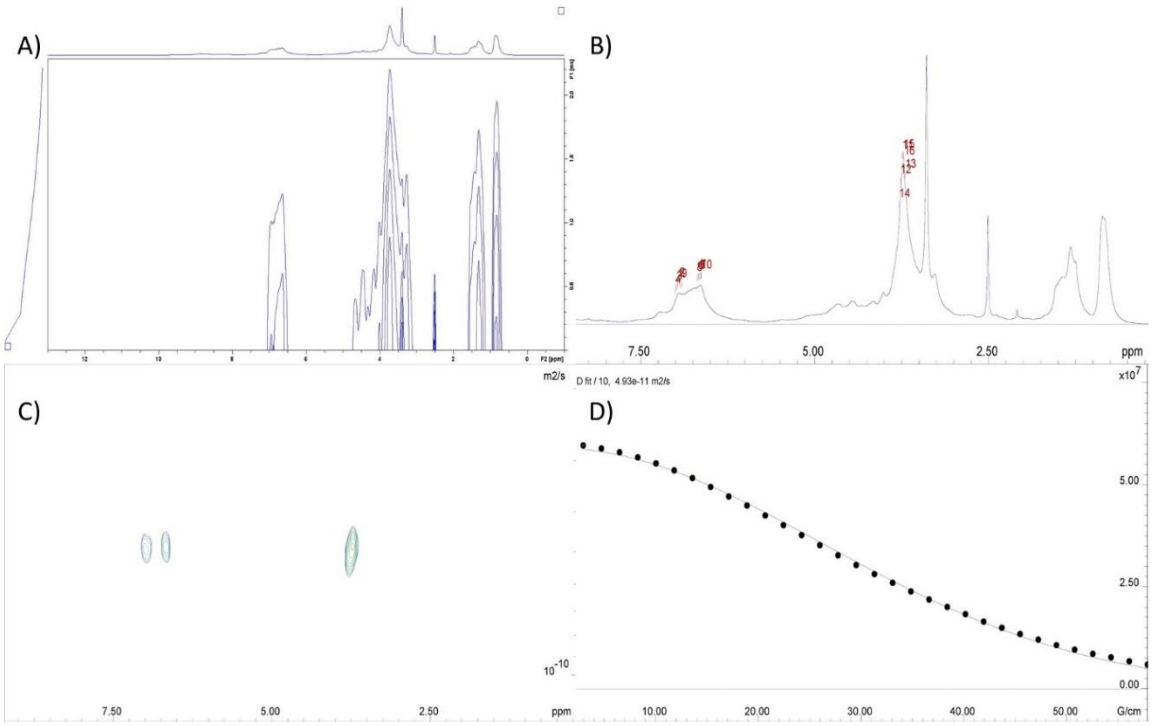

Figure S6: Workflow for DOSY NMR data processing. A) data were processed in TopSpin 4.1.1 software (line broadening (LB) = 10.00 Hz, ABSF1 to 13.00 ppm, and ABSF2 to -1.00 ppm). The baseline was manually corrected. B) The processed spectrum was opened in Dynamics Centre version 2.7.2 under the Diffusion tab. 15-20 points were selected on the spectrum in the G and S aromatic regions, and the methoxy region to ensure that signals that corresponded to lignin were chosen. C) The selected points were then further processed within Dynamics Centre version 2.7.2 to calculate a diffusivity that corresponded to each selected point. These diffusivities were then plotted and lay along the same point on the Y-axis. D) The fitting of each point to the curve was then checked to ensure good fit. If each point was determined to fit then the data set was exported and used to calculate an average diffusivity for the sample (Table S2). The average diffusivity of the sample was converted to an estimated MW using the Mark-Houwink-Sakurada equation[S7] (Equation S2, values of K and α previously determined experimentally to be 8.0478 and -0.6339 respectively by fitting to GPC data[S8]).

Point number	D / m^2s^{-1}
1	4.93×10^{-11}
2	4.94×10^{-11}
3	4.88×10^{-11}
4	4.86×10^{-11}
5	4.90×10^{-11}
6	4.87×10^{-11}
7	4.92×10^{-11}
8	4.85×10^{-11}
9	4.88×10^{-11}
10	4.93×10^{-11}
11	5.00×10^{-11}
12	5.11×10^{-11}
13	4.82×10^{-11}
14	5.11×10^{-11}
15	4.91×10^{-11}
16	4.86×10^{-11}
Average	4.92×10^{-11}

Table S2: Diffusivity values from DOSY NMR analysis of CPH butanosolv lignin from the optimised pretreatment.

Equation S2 $D = KMW^{-\alpha}$

Protocol	Diffusivity / m^2s^{-1}	Calc MW / Da
A (before precipitation)	6.09×10^{-11}	2630
A (after precipitation)	5.30×10^{-11}	3280
A (after CST)	4.15×10^{-11}	4820
B	5.66×10^{-11}	2950
C	5.12×10^{-11}	3460
D	4.92×10^{-11}	3680
D BoSL-OSE	1.17×10^{-10}	940

Table S3: DOSY NMR results obtained for various butanosolv lignins in this study. All values of the calculated molecular weight were within error (+/- 900 Da) except for that for the lignin present in the filtrate from the final purification in the optimised pretreatment which was significantly smaller. See Figure S12 legend for explanation of protocol descriptors used.

NMR Comparison Analysis of CPH Lignin and Model compounds

In this Section of the SI overlays of 3 different CPH lignin samples with models **3a**, **3b** and **3c** are provided to provide evidence in support of lignin acetylation (in some cases and not others, Figures S7 and S8). A further study using model compounds **S1a** and **S1b** is also provided and suggests that g-acylation (by an unidentified acid) is present in the CPH lignin isolated from the literature butanosolv pretreatment[S3] but not from the optimised pretreatment (Figure S9).

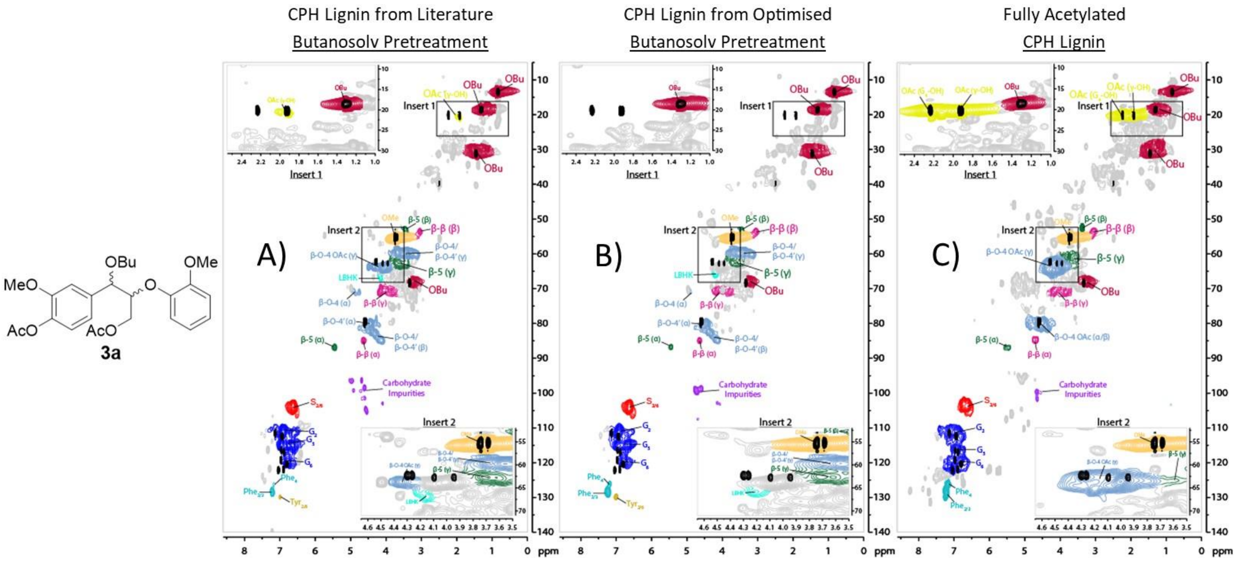

Figure S7: HSQC NMR (700 MHz, DMSO-d_6) analysis of overlays of fully acetylated phenolic G,G model **3a** (black) with A) CPH lignin from literature butanosolv pretreatment; B) CPH lignin from optimised butanosolv pretreatment; C) fully acetylated CPH lignin from the optimised pretreatment.

Analogous overlays to those using model compound **3a** (Figure S7) were also carried out using model compounds **3b** (Figure S8, spectra A-C) and **3c** (Figure S8, spectra D-F).

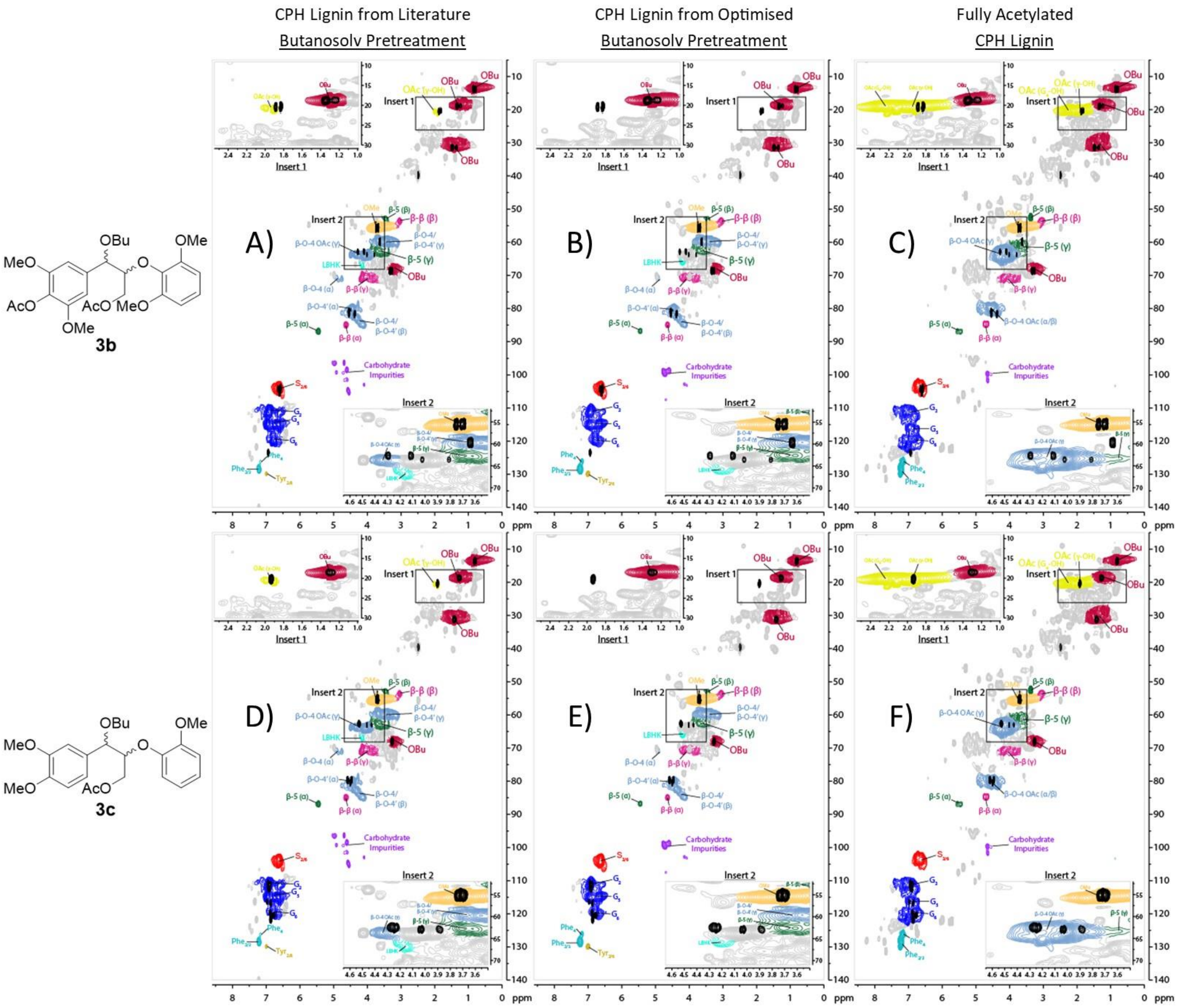

Figure S8: Overlays of HSQC NMR (700 MHz, DMSO-d$_6$) analysis of A) CPH lignin from the literature pretreatment[S3] with acetylated non-phenolic S,S model **3b** (black); B) CPH lignin from the optimised pretreatment with **3b** (black); C) fully acetylated CPH lignin from the optimised pretreatment with **3b** (black); D) CPH lignin from the literature pretreatment[S3] with acetylated non-phenolic G,G model **3c** (black); E) CPH lignin from the optimised pretreatment with **3c** (black); F) fully acetylated CPH lignin from the optimised pretreatment with **3c** (black). Inserts 1 and 2 in the spectra are highlighted as regions of highest significance as they contain signals that correspond to the acetylated primary γ-alcohol motif in the β-O-4 units.

Whilst assessing the Insert 2 region of the HSQC NMR spectra of the CPH lignin prepared using the original literature butanosolv pretreatment[S3] (*e.g.* Figures S9A and S9D), additional unassigned signals were also observed. One possible explanation is that these are due to the presence of acyl (as opposed to acetyl) groups on the γ-OCH2 in the β-O-4 units. Preliminary exploration of this is presented below through the synthesis of model compounds and HSQC NMR comparison studies.

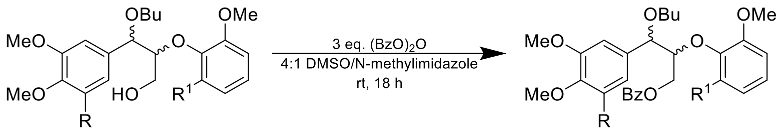

5b-c
b: R = R^1 = OMe
c: R = R^1 = H

S1a-b
a: R = R^1 = OMe, 76%
b: R = R^1 = H, 91%

Scheme S1: Synthesis of benzoylated models **S1a** and **S1b**

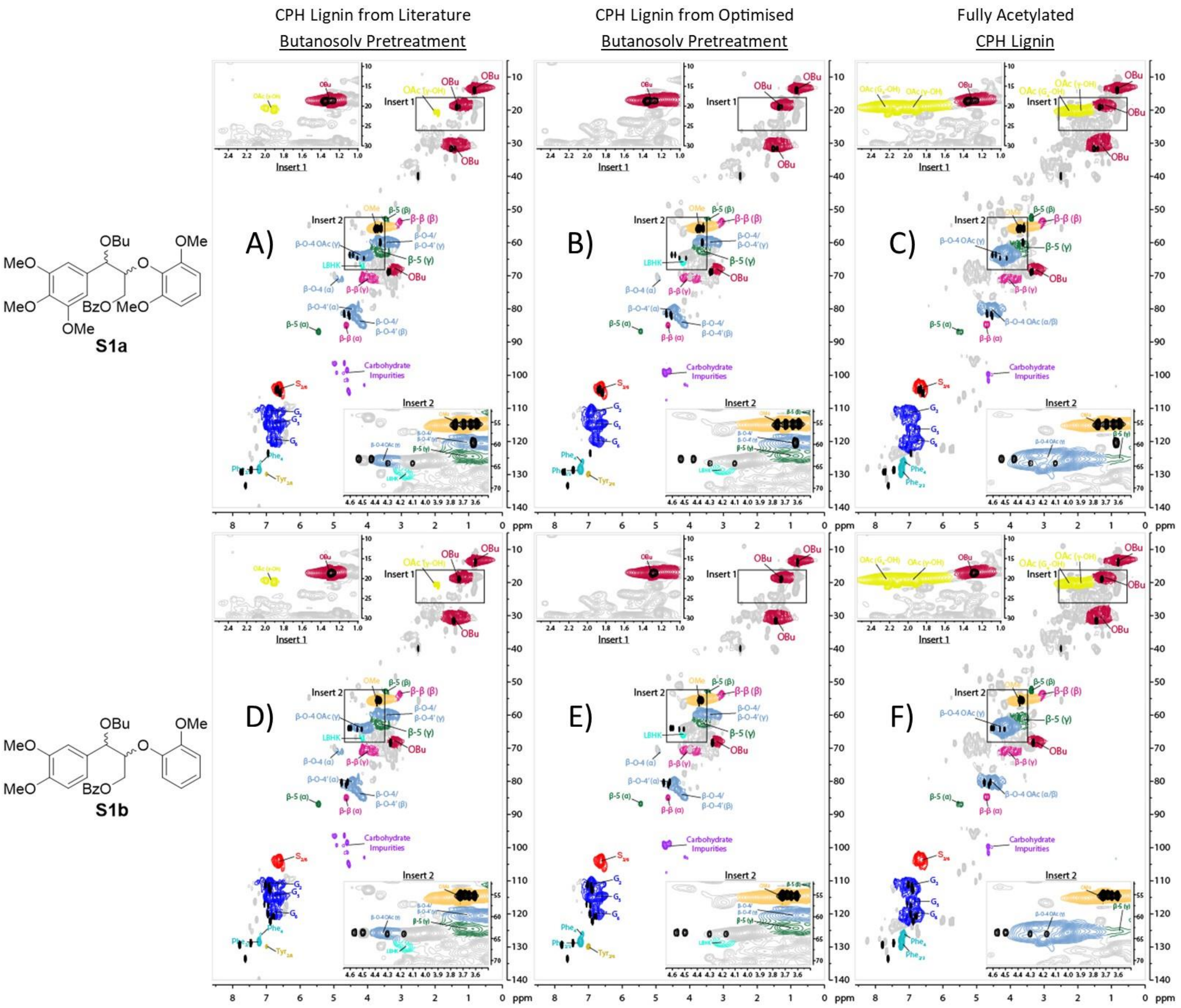

Figure S9: HSQC NMR (700 MHz, DMSO-d$_6$) spectra of A) CPH lignin from the literature pretreatment[S3] overlaid with benzoylated non-phenolic S,S model **S1a** (black); B) Lignin obtained using the optimised pretreatment overlaid with **S1a** (black); Lignin obtained using the optimised pretreatment that was fully acetylated and overlaid with **S1a** (black); D) CPH lignin from the literature pretreatment[S3] overlaid with benzoylated non-phenolic G,G model **S1b** (black); E) Lignin obtained using the optimised pretreatment overlaid with **S1b** (black); F) Lignin obtained using the optimised pretreatment that was fully acetylated and overlaid with **S1b** (black). Insert 1 in the spectra is provided for comparison of the acetylated model compounds and lignins shown in Figures S8 and S9. Insert 2 highlights the region of interest where signals that correspond to the acoylated (including benzoylated) primary γ-alcohol motif are located. The lignin itself is not benzoylated as many signals are not observed in the spectra however despite this the good overlap of the signal at ^{1}H 4.50/^{13}C 63.7 ppm indicates that it is likely that there is acylation/esterification at the γ-alcohol position of some of the β-O-4 units in the CPH butanosolv lignin obtained from the literature protocol.[S3] These signals may be due to the presence of cinnamate, *p*-coumarate[S9,S10] and/or *p*-hydroxybenzoate esters[S6,S11] known in the literature for other organosolv lignins. See Scheme S1 for synthesis of model compounds **S1a** and **S1b**.

CPH Lignin Modification and TGA

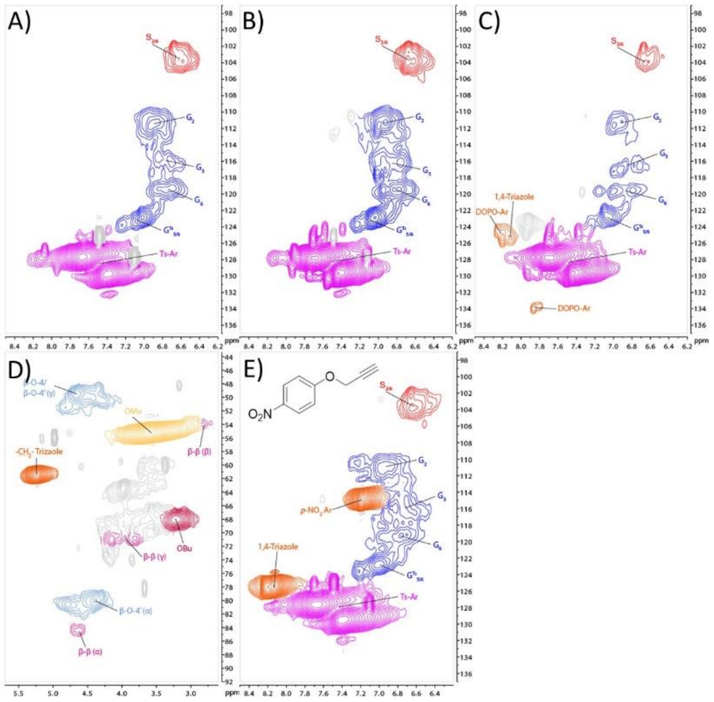

Figure S10: HSQC NMR (700 MHz, DMSO-d$_6$) analysis of A) aromatic region of Lignin-Ts; B) aromatic region of Lignin-N$_3$. The presence of large signals corresponding to OTs groups in Lignin-N$_3$ and Lignin-DOPO reflect the numerous tosylation sites in lignin that do not undergo the tosyl to azide conversion; C) aromatic region of Lignin-DOPO; D) linkage region of Lignin-N$_3$ after reaction with 1-nitro-4-(prop-2-yn-1-yloxy)benzene; E) aromatic region of lignin-N$_3$ reaction with 1-nitro-4-(prop-2-yn-1-yloxy)benzene (structure in insert).

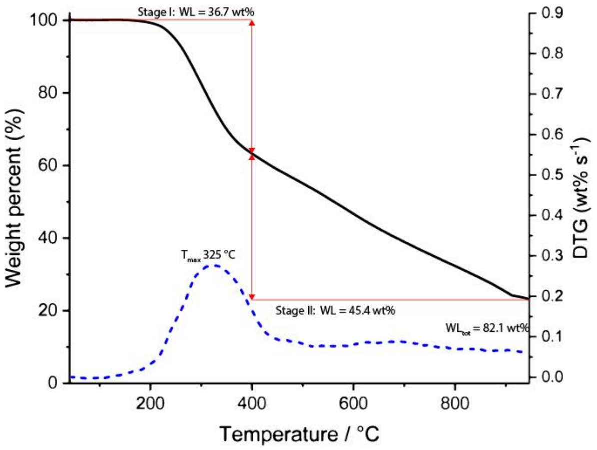

Figure S11: TGA (black) and DTG (blue, dashed) curves (N$_2$, 10 °C/min) of the control lignin prepared on reaction of Lignin-N$_3$ with 1-nitro-4-(prop-2-yn-1-yloxy)benzene (Figure S10E). The percentage weight loss during stage I and stage II pyrolysis is given by the red lines. The results for this control lignin confirmed a key role for the phosphorus-containing DOPO unit in reducing stage II weight loss through char formation.

Analysis of other CPH fractions

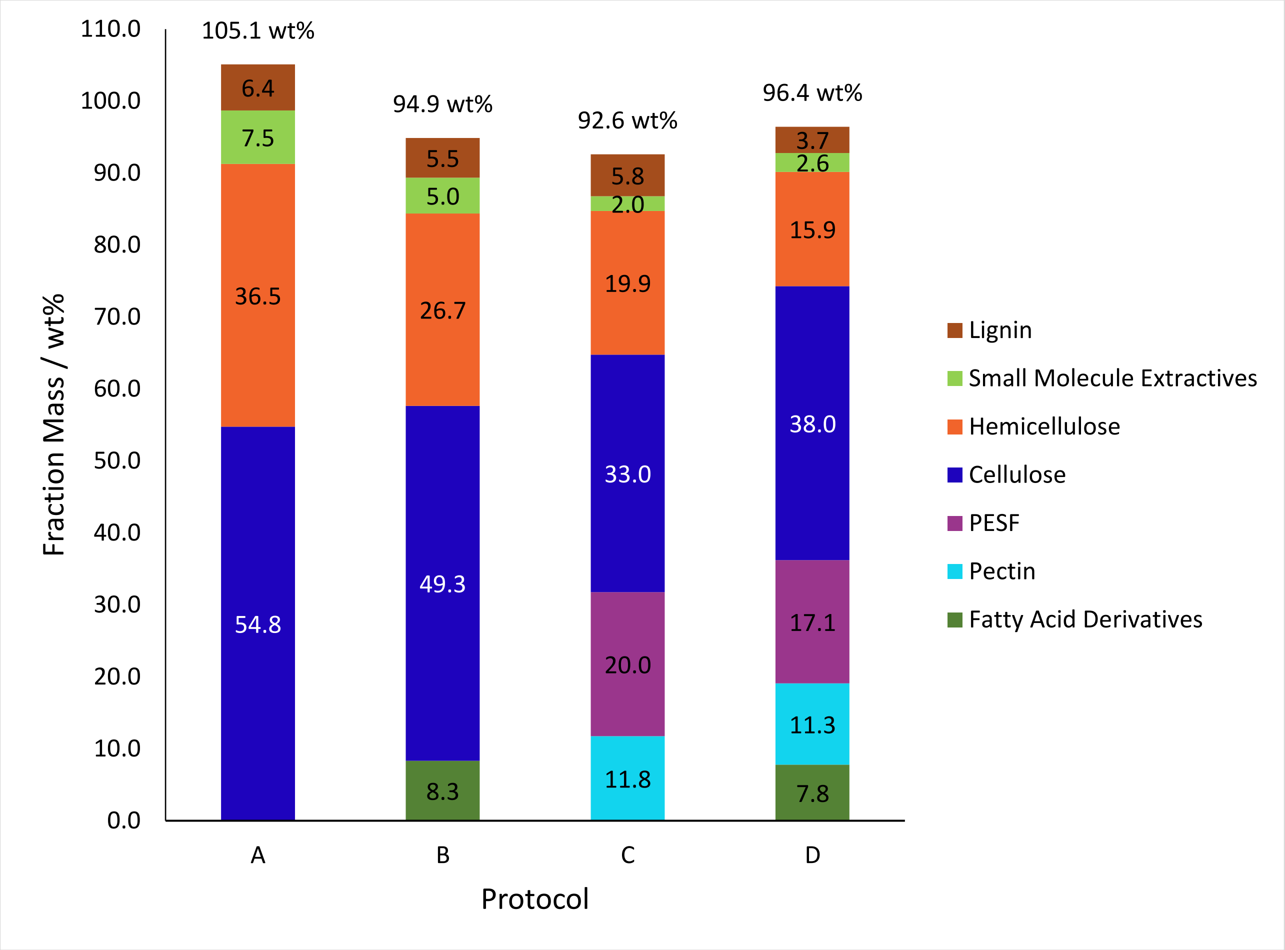

Figure S12: Mass balance obtained for CPH pretreatments across the varying butanosolv protocols. A = original literature butanosolv protocol[S3]; B = pre-wash with ethanol followed by butanosolv; C = hot water aqueous extraction followed by butanosolv; D = optimised butanosolv pretreatment for CPH involving ethanol and hot water extraction and then butanosolv. OSE = filtrate from final lignin purification step; PESF = pectin ethanol soluble fraction.

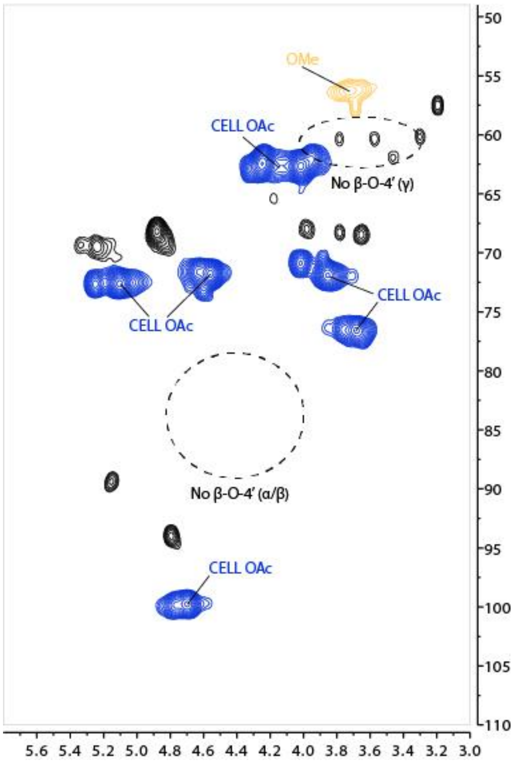

Figure S13: HSQC NMR (500 MHz, DMSO-d_6) analysis of the CPH cellulose pulp after acetyl bromide derivatisation. No signals associated with lignin structures could be identified (dashed black circle) and so it was concluded that the cellulose pulp was relatively pure. A signal corresponding to the methyl in methoxy groups was present and remains unexplained.

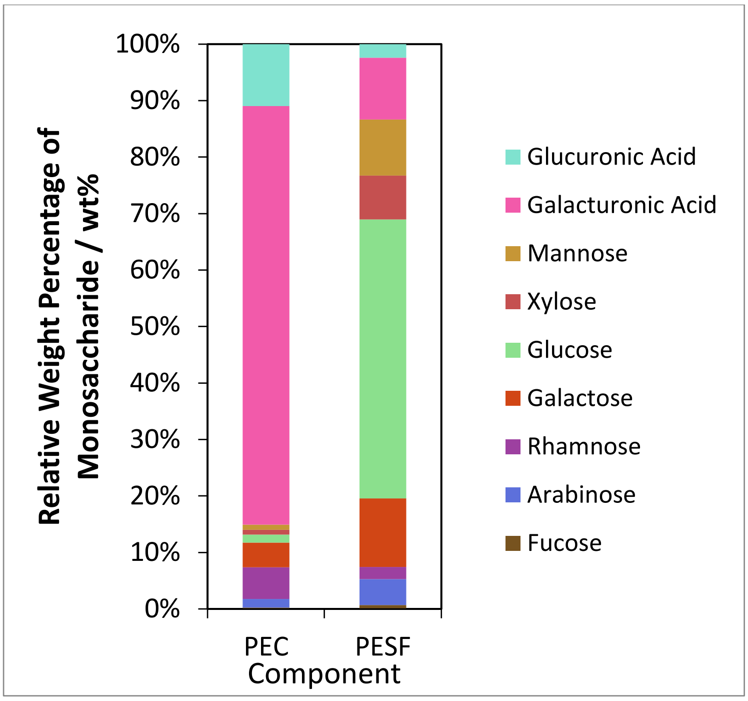

Figure S14: Monosaccharide content from HPAEC analysis of pectin and pectin ethanol soluble fraction (**PESF**) components.

Component	Monosaccharide Content (μg/mg)									
	GalA	Gal	Glu	Xyl	Ara	GluA	Rham	Mann	Fuc	Total
**PEC**	698.42	41.52	12.95	7.89	14.52	103.07	52.76	8.60	2.08	**941.83**
**PESF**	10.06	11.19	45.46	7.18	4.24	2.21	1.98	9.12	0.61	**92.05**

Table S4: Monosaccharide content from HPAEC analysis of pectin and **PESF** fractions.

Component	Metal Content / mg/g biomass		
	K	Ca	Mg
Whole CPH	33.2	6.71	2.76
Pectin	2.37	1.19	0.37

Table S5: Concentrations of K, Ca and Mg in whole CPH and associated with CPH pectin, as proportions of CPH mass, measured by digestion in nitric acid and Inductively Coupled Plasma-Optical Emission Spectrometry. The majority of the K, Ca and Mg were found in the soluble components obtained by the extraction protocols (data not shown), but pectin retained K^+, Ca^{2+} and Mg^{2+} as counterions to its content of carboxylate (COO^-) groups.

Solid State ^{13}C NMR analysis of Pectin

The degree of methylation (DM) of pectin can be calculated (Equation S3) from solid-state ^{13}C CP MAS NMR spectra following the procedure of Zhu *et al.*,[S12] based on the integrated intensity of the methoxy carbon relative to the C6 carbons,

Equation S3
$$DM = \frac{A_{OCH_3}}{A_{C6}} \times 100\%$$

which gives a measure of how many OCH_3 are present per C6 (the relevant OCH_3 and C6 signals are highlighted in Figure S15a). However, as noted by Zhu *et al.*, the cross-polarisation experiment transfers magnetisation from ^{1}H to ^{13}C, so the transfer dynamics are likely to be very different for a primary O*C*H_3 carbon and a quaternary R*C*O_2R carbon and this may lead to an unreliable measure of DM. Therefore, perhaps a more reliable approach (Equation S4) would be to compare the relative intensities of the *C*O_2H and *C*O_2CH_3 signals from C6 to determine directly how many of the C6 have been methylated,

Equation S4
$$DM' = \frac{A_{C6\text{-}OOCH_3}}{A_{C6\text{-}OOCH_3} + A_{C6\text{-}OOH}} \times 100\%$$

As shown in the inset in Figure S15a, the challenge with this latter approach is that the *C*O_2H and *C*O_2CH_3 signals have very similar chemical shifts and the pectin matrix is disordered on the atomic scale, such that the two signals cannot be directly resolved. Lineshape fitting (here using DMFit[S13]) is unreliable when attempting to fit the C6 signal to two Gaussian-Lorentzian lines with freely varied shift, width and G/L ratio, which leads to a much larger uncertainty in DM′ than in DM. Indeed, Zhu *et al.* observed relatively large discrepancies between DM, DM′ and DM determined independently using IR spectroscopy. Table S6 reports the integrated intensities for the two C6 signals and the OCH_3 signal obtained for the present sample of CPH pectin, yielding DM and DM′, respectively, of 34% and 7%.

The degree of acetylation (DA) can be calculated in a similar way to DM, using the intensity of the acetyl CH_3 signal (indicated in Figure S11a) and the intensity of the C6 signal,

Equation S5
$$DA = \frac{A_{CH_3\text{-acetyl}}}{A_{C6}} \times 100\%$$

However, as noted above, this measure of DA will be susceptible to the same systematic errors arising from the non-quantitative nature of the CP experiment. From the spectral integrals (Table S6), DA for the present CPH pectin can be calculated as 57%.

Figure S15b plots the variation in the apparent DM, DM′ and DA as a function of the CP contact time for one sample of CPH pectin, showing that, as one might expect, DM′ is a far more consistent measure than DM or DA. In the sample studied here, DM ranged from 28% to 94% whereas DM′ ranged from 6 to 9%. For the same set of experiments, DA ranged from 48% to 130%, implying both the C2 and C3 positions of GalA monomers in pectin could be acetylated. However, as the CP dynamics of CH_3 in acetyl and methoxy groups are similar, the DA/DM ratio (not shown) remained roughly constant at around 1.5(1) acetyl groups per methoxy group.

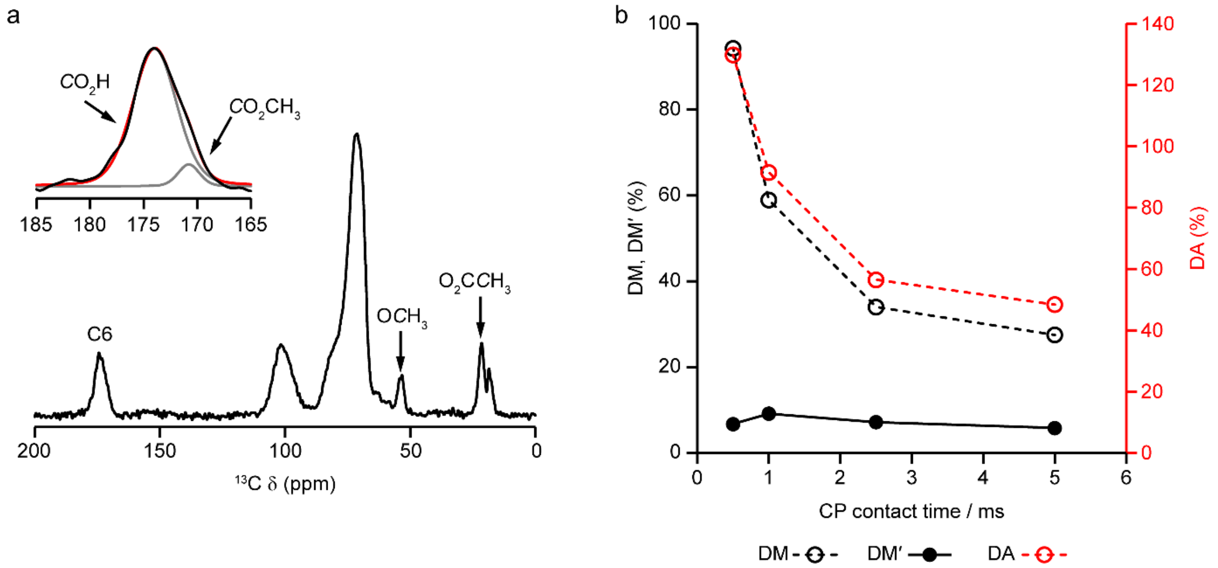

Figure S15: (a) ^{13}C CP MAS NMR spectrum of CPH **PEC** showing the C6, OCH_3 and acetyl CH_3 signals used for calculating DM and DA. The inset shows the best fit decomposition (with a G/L ratio of 0.8) of the C6 signal into the CO_2H and CO_2CH_3 components used for calculating DM′. (b) Plot of variation in apparent DM, DM′ and DA for the same sample as a function of CP contact time.

Signal	integral / arb. units
*C*O2H	315
*C*O2CH3	24
C6 total	339
O*C*H3	116
O2C*C*H3	192

Table S6: Integrals of relevant ^{13}C signals used to calculate DM, DM′ and DA for CPH pectin.

A)

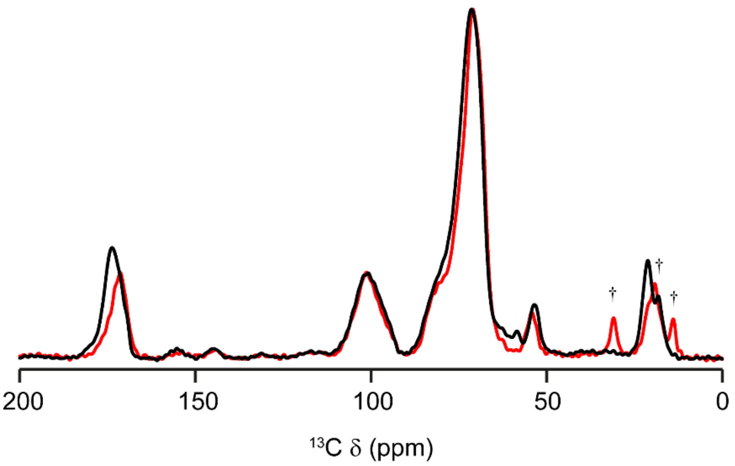

B)

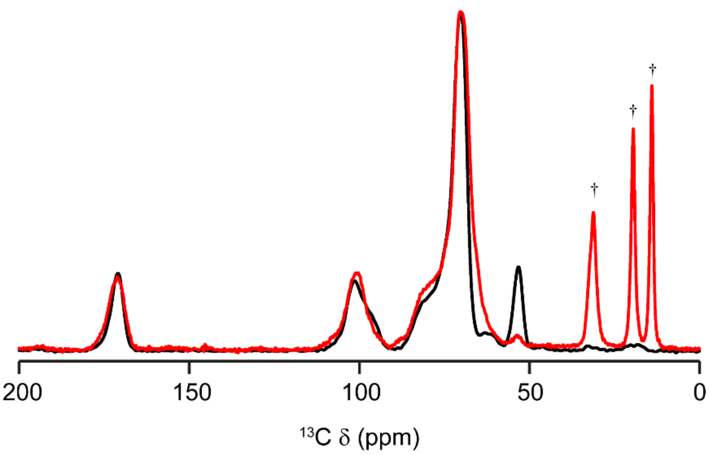

Figure S16: ^{13}C CP MAS NMR spectra of: A) CPH pectin before (black) and after (red) butanosolv treatment. Signals arising from butoxylation are indicated with daggers (†); B) apple pectin before (black) and after (red) butanosolv treatment. Signals arising from butoxylation are indicated with daggers (†). These studies provide preliminary information on the impact of a butanosolv pretreatment on the structure of pectins. In brief, methyl ester groups present in the pectin are trans-esterified to the corresponding butyl esters as well as pectin depolymerisation occurring (data not shown).

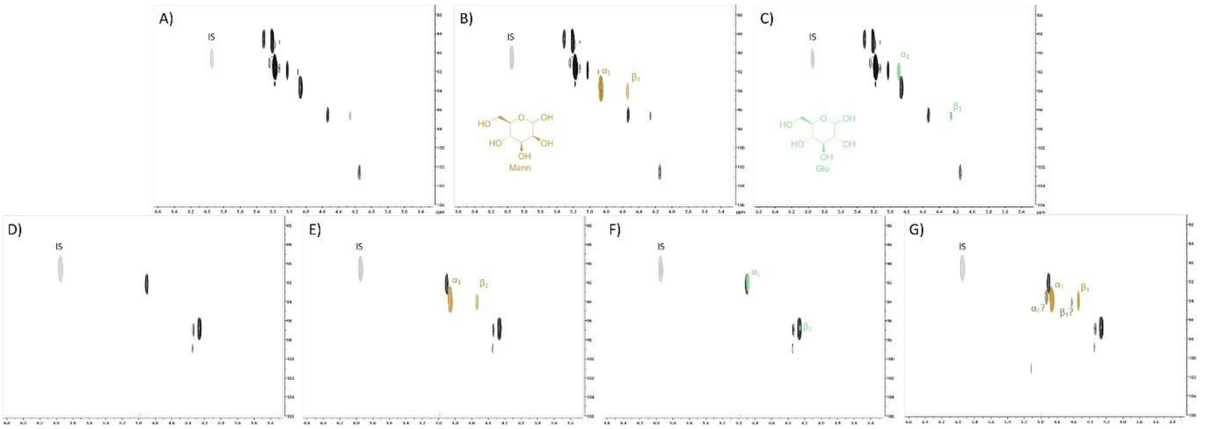

Figure S17: A region of the HSQC NMR (700 MHz, DMSO-d$_6$) spectra of A) **PESF** (black) obtained using the optimal pretreatment overlaid with the HSQC NMR spectra of: B) mannose (Mann, yellow); C) glucose (Glu, light green). D) HSQC NMR (700 MHz, DMSO-d$_6$) spectra of a TFA-hydrolysed sample of the **PESF** obtained using the optimal pretreatment (black) overlaid with the HSQC NMR analysis of: E) Mann (yellow); F) Glu (green). G) HSQC NMR (700 MHz, DMSO-d$_6$) spectrum of a TFA-hydrolysed Mann sample showing starting Mann (yellow) and a currently unassigned Mann hydrolysis product (olive green). All spectra contain α-D-glucose pentaacetate as an internal standard (IS, grey). Good overlay of signals in (i) the **PESF** with the α-1 signal of Mann and (ii) the α-1 signal of Glu in the TFA-hydrolysed **PESF** supported the tentative assignment of mannose and glucose as being present in the oligo- and polysaccharides in the **PESF**. This was in agreement with the results of the HPAEC analysis of TFA-hydrolysed **PESF** (Figure S15 and Table S4). The currently assigned signals of TFA-treated Mann did not show good overlap with any signals in the TFA-hydrolysed **PESF** and so it is likely that the change in the relative ratio of Glu:Mann signals before and after TFA-hydrolysis is due to an increase in Glu monomers in the mixture and not a transformation of Mann.

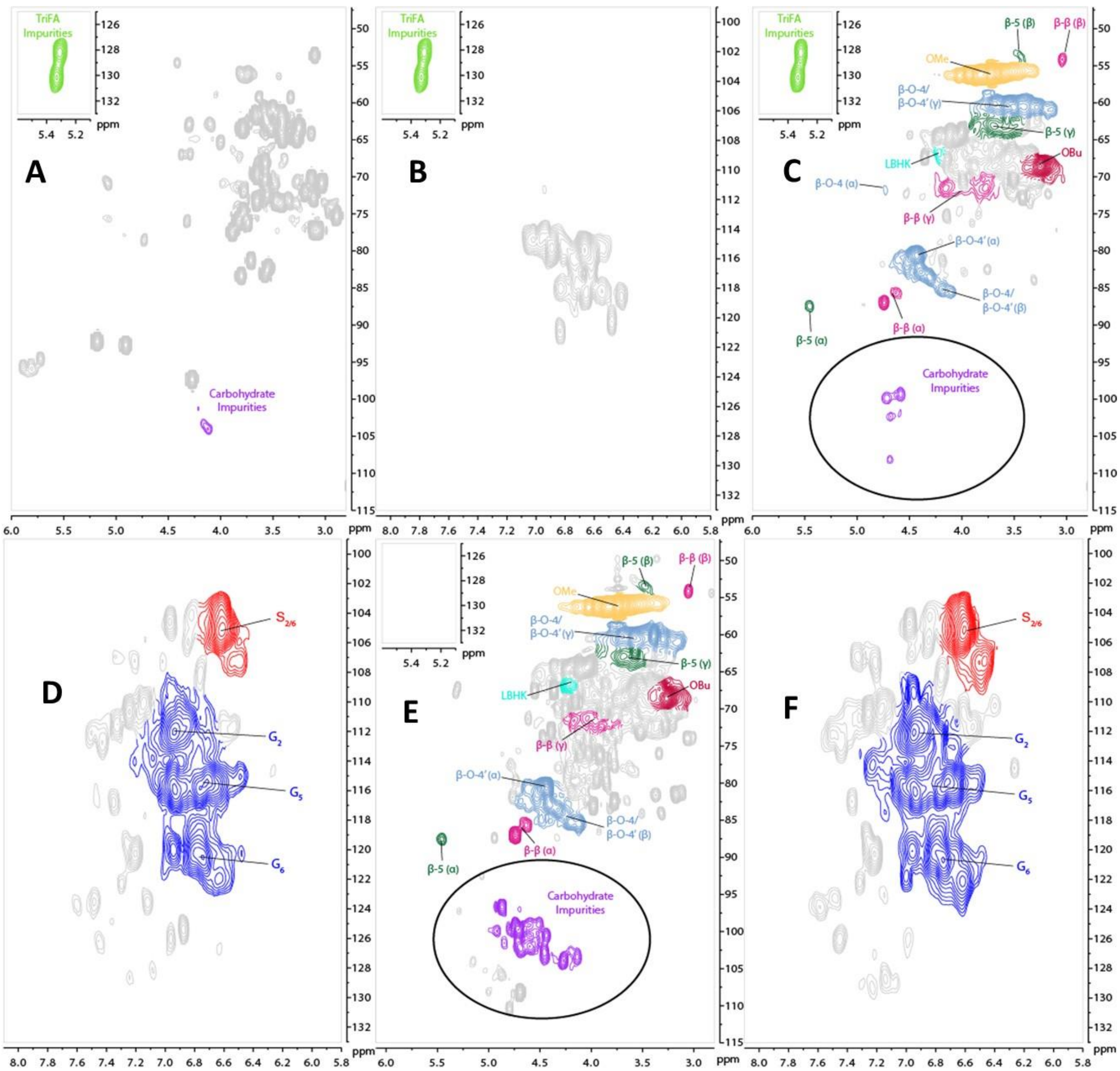

Figure S18: HSQC NMR (700 MHz, DMSO-d_6) analysis of the material isolated following concentration of the EtOH pre-wash: A) linkage region; B) aromatic region with the insert highlighting key signals from the fatty acids present in the sample; analysis of the filtrate isolated during the final purification of the lignin: C) linkage region highlighting the presence of carbohydrates and fatty acids (despite the EtOH pre-wash); D) aromatic region; analysis of the filtrate after partial purification by column chromatography: E) linkage region; F) aromatic region.

Model Compound Synthesis

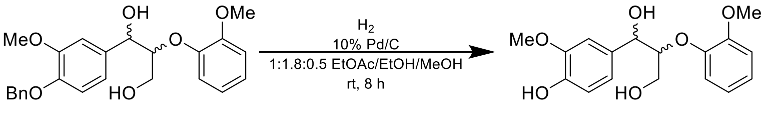

S2 **4a** 92%

Scheme S2: Synthesis of model compound **4a**

<u>1-(4-hydroxy-3-methoxyphenyl)-2-(2-methoxyphenoxy)propane-1,3-diol **4a**</u>[S14]

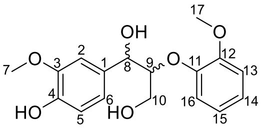

S2 (prepared by a literature procedure,[S14] 20.6 g, 50.1 mmol, *major/minor 1:0.6*) and 10% Pd/C (1.08 g, 0.10 eq.) were stirred in 1:1.8:0.5 EtOAc/EtOH/MeOH (330 mL) under H_2 atmosphere at room temperature for 8 hours. The solution was filtered through a pad of Celite and concentrated under reduced pressure to afford diastereomeric mixture **4a** (14.7 g, 45.9 mmol, 92%, *major/minor 1:0.6*) as a colourless viscous oil. Analytical data were in accordance with that previously reported.[S14]

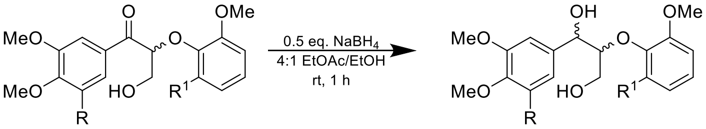

S3a-b
a: R = R^1 = OMe
b: R = R^1 = H

4b-c
b: R = R^1 = OMe, 88%
c: R = R^1 = H, 91%

Scheme S3: Synthesis of model compounds **4b-c**

<u>2-(2,6-dimethoxyphenoxy)-1-(3,4,5-trimethoxyphenyl)propane-1,3-diol **4b**</u>[S5]

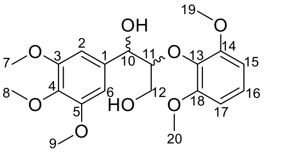

S3a (prepared by a literature protocol,[S5] 9.44 g, 24.1 mmol, 1.0 eq.) was dissolved in 4:1 EtOAc/EtOH (100 mL) and $NaBH_4$ (0.46 g, 12.1 mmol, 0.50 eq.) was added portionwise then allowed to stir at room temperature for 1 hour. The reaction mixture was diluted with sat. aq. NH_4Cl solution (150 mL) and extracted with EtOAc (3 x 60 mL). The combined organic extracts were washed with brine (1 x 60 mL), dried over $MgSO_4$, concentrated under reduced pressure, and purified by column chromatography eluting with EtOAc/hexane (10-70%) to afford diastereomeric mixture **4b** (8.35 g, 21.2 mmol, 88%, *major/minor 1:0.3*) as a pale yellow viscous oil. Analytical data were in accordance with that previously reported.[S5]

<u>1-(3,4-dimethoxyphenyl)-2-(2-methoxyphenoxy)propane-1,3-diol **4c**</u>[S5]

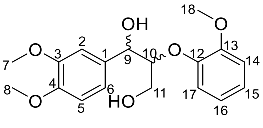

S3b (prepared by a literature protocol,[S5] 6.55 g, 19.7 mmol, 1 eq.) was dissolved in 4:1 EtOAc/EtOH (70 mL) and $NaBH_4$ (0.39 g, 10.3 mmol, 0.50 eq.) was added portionwise then allowed to stir at room temperature for 1 hour. The reaction mixture was diluted with sat. aq. NH_4Cl (100 mL) and extracted with EtOAc (3 x 50 mL). The combined organic extracts were washed with brine (1 x 50 mL), dried over $MgSO_4$, concentrated under reduced pressure, and purified by column chromatography eluting with EtOAc/hexane (10-70%) to afford diastereomeric mixture **4c** (6.01 g, 18.0 mmol, 91%, *major/minor 1:0.7*) as a colourless viscous oil. Analytical data were in accordance with that previously reported.[S5]

4-(1-butoxy-3-hydroxy-2-(2-methoxyphenoxy)propyl)-2-methoxyphenol **5a**[S5]

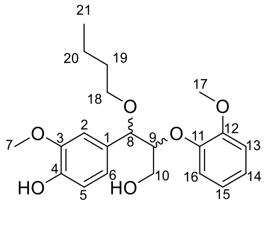

4a (1.58 g, 4.93 mmol, *major/minor 1:0.6*) was dissolved in *n*-butanol (16 mL) and heated to reflux. 4M HCl solution (1.6 mL) was then added and the mixture stirred for 20 minutes then removed from heat and quenched with saturated sat. aq. bicarbonate solution (20 mL). The mixture was extracted with ethyl acetate (3 x 20 mL) and washed with brine (1 x 20 mL). The combined organic extracts were dried over $MgSO_4$ and concentrated under reduced pressure. The crude product was purified by column chromatography eluting with EtOAc/hexane (10% to 70%) to afford diastereomeric mixture **5a** (1.21 g, 3.18 mmol, 65%, *major/minor 1:0.8*) as a pale yellow viscous oil. Analytical data were in accordance with that previously reported.[S5]

3-butoxy-2-(2,6-dimethoxyphenoxy)-3-(3,4,5-trimethoxyphenyl)propan-1-ol **5b**[S5]

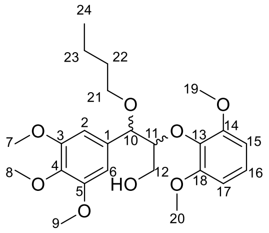

4b (1.03 g, 2.61 mmol, *major/minor 1:0.3*) was dissolved in n-butanol (10 mL) and heated to reflux. 4M HCl solution (1.0 mL) was then added and the mixture stirred for 20 minutes then removed from heat and quenched with saturated sat. aq. bicarbonate solution (15 mL). The mixture was extracted with ethyl acetate (3 x 15 mL) and washed with brine (1 x 15 mL). The combined organic extracts were dried over $MgSO_4$ and concentrated under reduced pressure. The crude product was purified by column chromatography eluting with EtOAc/hexane (10% to 70%) to afford diastereomeric mixture **5b** (0.49 g, 1.09 mmol, 42 %, *major/minor 1:0.5*) as a pale yellow viscous oil. Analytical data were in accordance with that previously reported.[S5]

3-butoxy-3-(3,4-dimethoxyphenyl)-2-(2-methoxyphenoxy)propan-1-ol **5c**[S5]

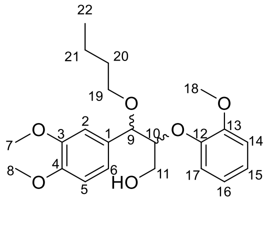

4c (1.07 g, 3.21 mmol, *major/minor 1:0.7*) was dissolved in n-butanol (10 mL) and heated to reflux. 4M HCl (1.0 mL) was then added and the mixture stirred for 20 min then removed from heat and quenched with saturated sat. aq. bicarbonate solution (15 mL). The mixture was extracted with ethyl acetate (3 x 15 mL) and washed with brine (1 x 15 mL). The combined organic extracts were dried over $MgSO_4$ and concentrated under reduced pressure. The crude product was purified by column chromatography eluting with EtOAc/hexane (10% to 70%)

to afford diastereomeric mixture **5c** (1.11 g, 2.83 mmol, 89%, *major/minor 1:0.8*) as a colourless viscous oil. Analytical data were in accordance with that previously reported.[S5]

4-(3-acetoxy-1-butoxy-2-(2-methoxyphenoxy)propyl)-2-methoxyphenyl acetate **3a**

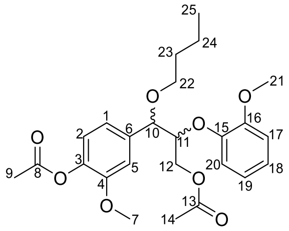

5a (1.07 g, 2.81 mmol, 1 eq., *major/minor 1:0.8*) was dissolved in pyridine (7.50 mL) and acetic anhydride (2.6 mL, 27.5 mmol, 10.0 eq.) added dropwise. The solution was stirred at room temperature for 18 hours then diluted with 0.1M HCl solution (10 mL) and extracted with EtOAc (3 x 30 mL). The combined organic extracts were dried over $MgSO_4$ and concentrated under reduced pressure. The crude product was purified by column chromatography eluting with EtOAc/hexane (10% to 70%) to afford diastereomeric mixture **3a** (1.21 g, 2.63 mmol, 93%, *major/minor 1:0.8*) as a pale yellow oil. **IR** (ATR) ν_{max}/cm^{-1} 2955 (C-H), 1740 (C=O), 1597 (C-H Ar), 1501 (C-H), 1254 (C-O), 1196 (C-O), 1034 (C-O). **HRMS** (ESI) calculated for $C_{25}H_{32}O_8Na$ $[M+Na]^+$ 483.1995; found 483.1987.

3-butoxy-2-(2,6-dimethoxyphenoxy)-3-(3,4,5-trimethoxyphenyl)propyl acetate **3b**

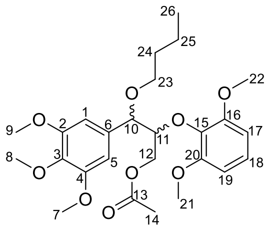

5b (203 mg, 0.45 mmol, 1.00 eq., *major/minor 1:0.5*) was dissolved in pyridine (2 mL) and acetic anhydride (0.21 mL, 2.21 mmol, 5.00 eq.) was added dropwise. The solution was stirred at room temperature for 18 hours then diluted with 0.1M HCl solution (5 mL) and extracted with EtOAc (3 x 15 mL). The combined organic extracts were dried over $MgSO_4$ and concentrated under reduced pressure. The crude product was purified by column chromatography eluting with EtOAc/hexane (10% to 70%) to afford diastereomeric mixture **3c** (220.4 mg, 0.45 mmol, 83%, *major/minor 1:0.5*) as a pale yellow viscous oil. **IR** (ATR) ν_{max}/cm^{-1} 2940 (C-H), 1736 (C=O), 1591 (C-H Ar), 1477 (C-H), 1233 (C-O), 1109 (C-O), 1045 (C-O). **HRMS** (ESI) calculated for $C_{26}H_{36}O_9Na$ $[M+Na]^+$ 515.2257; found 515.2238.

3-butoxy-3-(3,4-dimethoxyphenyl)-2-(2-methoxyphenoxy)propyl acetate **3c**

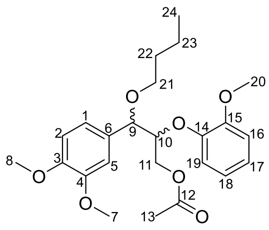

5c (158 mg, 0.40 mmol, 1.00 eq., *major/minor 1:0.8*) was dissolved in pyridine (2 mL) and acetic anhydride (0.20 mL, 2.12 mmol, 5.00 eq.) was added dropwise. The solution was stirred at room temperature for 18 hours then diluted with 0.1M HCl solution (5 mL) and extracted with EtOAc (3 x 15 mL). The combined organic extracts were dried over $MgSO_4$ and concentrated under reduced pressure. The crude product was purified by column chromatography eluting with EtOAc/hexane (10% to 70%) to afford diastereomeric mixture **3b** (125 mg, 0.29 mmol, 71%, *major/minor 1:0.6*) as a clear viscous oil. **IR** (ATR) ν_{max}/cm^{-1} 2951 (C-H), 1740 (C=O), 1593 (C-H Ar),

1500 (C-H), 1231 (C-O), 1126 (C-O), 1026 (C-O). **HRMS** (ESI) calculated for $C_{24}H_{32}O_7Na$ $[M+Na]^+$ 455.2046; found 455.2040.

<u>4-(1-butoxy-2-(2-methoxyphenoxy)-3-(tosyloxy)propyl)-2-methoxyphenyl 4-methylbenzenesulfonate **8a**</u>[S5]

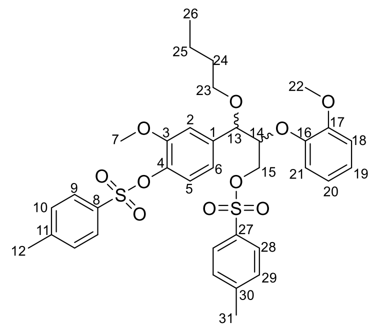

5a (0.44 g, 1.17 mmol, 1 eq. *major/minor 1:0.8*), tosyl chloride (0.67 g, 3.52 mmol, 3 eq.), DMAP (0.07 g, 0.59 mmol, 0.50 eq.) and NEt_3 (0.50 mL, 3.59 mmol, 3 eq.) were stirred in DCM (5 mL) at room temperature for 18 hours. The mixture was diluted with sat. aq. ammonium chloride solution (10 mL) and extracted with DCM (3 x 10 mL). The combined organic extracts were dried over $MgSO_4$ and concentrated under reduced pressure. The crude product was purified by column chromatography eluting with EtOAc/hexane (10% to 40%) to afford diastereomeric mixture **8a** (0.76 g, 1.11 mmol, 95%, *major/minor 1:0.8*) as a pale yellow viscous oil. Analytical data were in accordance with that previously reported.[S5]

<u>3-butoxy-2-(2,6-dimethoxyphenoxy)-3-(3,4,5-trimethoxyphenyl)propyl 4-methylbenzenesulfonate **8b**</u>[S5]

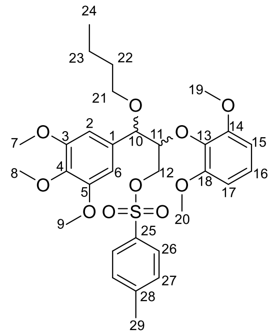

5b (0.42 g, 0.93 mmol, 1 eq. *major/minor 1:0.5*), tosyl chloride (0.53 g, 2.80 mmol, 3.00 eq.), DMAP (0.06 g, 0.48 mmol, 0.50 eq.) and NEt_3 (0.39 mL, 2.80 mmol, 3.00 eq.) were stirred in DCM (5 mL) at room temperature for 18 hours. The mixture was diluted with sat. aq. ammonium chloride solution (10 mL) and extracted with DCM (3 x 10 mL). The combined organic extracts were dried over $MgSO_4$ and concentrated under reduced pressure. The crude product was purified by column chromatography eluting with EtOAc/hexane (10% to 70%) to afford diastereomeric mixture **8b** (0.55 g, 0.91 mmol, 98%, *major/minor 1:0.4*) as a yellow viscous oil. Analytical data were in accordance with that previously reported.[S5]

<u>3-butoxy-3-(3,4-dimethoxyphenyl)-2-(2-methoxyphenoxy)propyl 4-methylbenzenesulfonate **8c**</u>[S5]

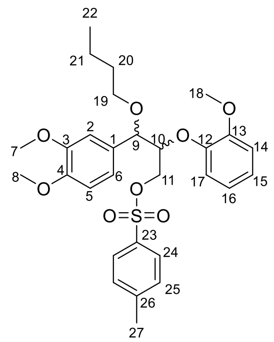

5c (0.24 g, 0.62 mmol, 1.00 eq. *major/minor 1:0.8*), tosyl chloride (0.36 g, 1.89 mmol, 3.00 eq.), DMAP (0.04 g, 0.35 mmol, 0.50 eq.) and NEt_3 (0.26 mL, 1.87 mmol, 3.00 eq.) were stirred in DCM (5 mL) at room temperature for 18 hours. The mixture was diluted with sat. aq. ammonium chloride solution (10 mL) and extracted with DCM (3 x 10 mL). The combined organic extracts were dried over $MgSO_4$ and concentrated under reduced pressure. The crude product was purified by column chromatography eluting with EtOAc/hexane (10% to 60%) to afford diastereomeric mixture **8c** (0.32 g, 0.59 mmol, 95%, *major/minor 1:0.8*) as a pale yellow viscous oil. Analytical data were in accordance with that previously reported.[S5]

4-(3-azido-1-butoxy-2-(2-methoxyphenoxy)propyl)-2-methoxyphenyl 4-methylbenzenesulfonate **9a**[S5]

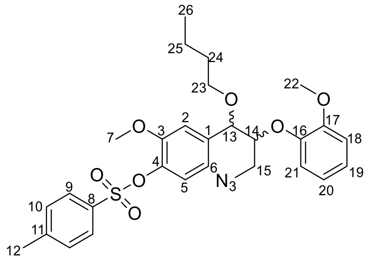

8a (0.73 g, 1.07 mmol, 1.00 eq., *major/minor 1:0.8*) and sodium azide (0.35 g, 5.37 mmol, 5.00 eq.) were stirred in DMF (7.5 mL) at 50 °C for 18 hours. The suspension was diluted with water (15 mL) and extracted with EtOAc (3 x 15 mL). The combined organic extracts were washed with brine (1 x 20 mL), dried over $MgSO_4$ and concentrated under reduced pressure. The crude product was purified by column chromatography eluting with EtOAc/hexane (10% to 70%) to afford diastereomeric mixture **9a** (0.50 g, 0.91 mmol, 85%, *major/minor 1:0.7*) as a colourless viscous oil. Analytical data were in accordance with that previously reported.[S5]

5-(3-azido-1-butoxy-2-(2,6-dimethoxyphenoxy)propyl)-1,2,3-trimethoxybenzene **9b**[S5]

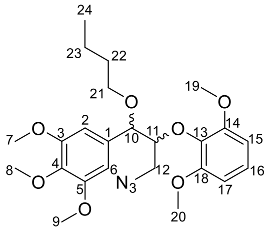

8b (0.51 g, 0.84 mmol, 1 eq. *major/minor 1:0.4*) and sodium azide (0.28 g, 4.24 mmol, 5.00 eq.) were stirred in DMF (5.0 mL) at 50 °C for 18 hours. The suspension was diluted with water (15 mL) and extracted with EtOAc (3 x 15 mL). The combined organic extracts were washed with brine (1 x 20 mL), dried over $MgSO_4$ and concentrated under reduced pressure. The crude product was purified by column chromatography eluting with EtOAc/hexane (10% to 70%) to afford diastereomeric mixture **9b** (0.23 g, 0.48 mmol, 58%, *major/minor 1:0.6*) as a pale yellow viscous oil. Analytical data were in accordance with that previously reported.[S5]

4-(3-azido-1-butoxy-2-(2-methoxyphenoxy)propyl)-1,2-dimethoxybenzene **9c**[S5]

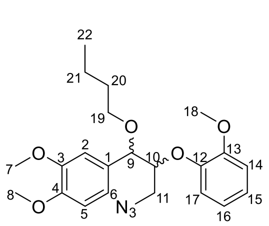

8c (0.31 g, 0.57 mmol, 1.00 eq., *major/minor 1:0.8*) and sodium azide (0.18 g, 2.83 mmol, 5.00 eq.) were stirred in DMF (3.0 mL) at 50 °C for 18 hours. The suspension was diluted with water (10 mL) and extracted with EtOAc (3 x 15 mL). The combined organic extracts were washed with brine (1 x 20 mL), dried over $MgSO_4$ and concentrated under reduced pressure. The crude product was purified by column chromatography eluting with

EtOAc/hexane (10% to 70%) to afford diastereomeric mixture **9c** (0.22 g, 0.52 mmol, 91%, *major/minor 1:0.2*) as a pale yellow viscous oil. Analytical data were in accordance with that previously reported.[S5]

6-(prop-2-yn-1-yloxy)dibenzo[c,e][1,2]oxaphosphinine 6-oxide **10**

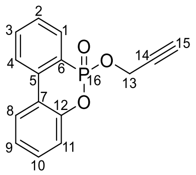

DOPO **1** (3.01 g, 13.9 mmol, 1.00 eq.) and N-chlorosuccinimide (2.04 g, 15.3 mmol, 1.1 eq.) were suspended in toluene (30 mL) and stirred at room temperature for 12 hours. The suspension was then filtered under vacuum, the filtrate concentrated and suspended in DCM (40 mL). To the suspension was added NEt_3 (2.14 mL, 15.4 mmol, 1.10 eq.) and propargyl alcohol (0.89 mL, 15.3 mmol, 1.10 eq.) dropwise and stirred at room temperature for 12 hours. The suspension was diluted with 0.1 M HCl solution (50 mL) and extracted with DCM (3 x 40 mL). The combined organic extracts were washed with water (3 x 20 mL), dried over $MgSO_4$ and concentrated under reduced pressure. The crude product was purified by column chromatography eluting with EtOAc/hexane (10 - 90%) to afford racemic mixture **10** (3.28 g, 12.1 mmol, 87%) as a white powder. A sample of **10** was slowly recrystalised from DCM which afforded crystals of sufficient quality for X-ray crystallographic analysis: CCDC 2179378. **mp** 119-120 °C. **IR** (ATR) ν_{max}/cm^{-1} 3225 (C-H alkyne), 2120 (CΞC), 1263 (P=O), 1203 (P-O-Ar), 1020 (P-C aromatic), 934 (P-O-Ar). **HRMS** (ESI) calculated for $C_{15}H_{12}O_3P$ $[MH]^+$ 271.0524; found 271.0517.

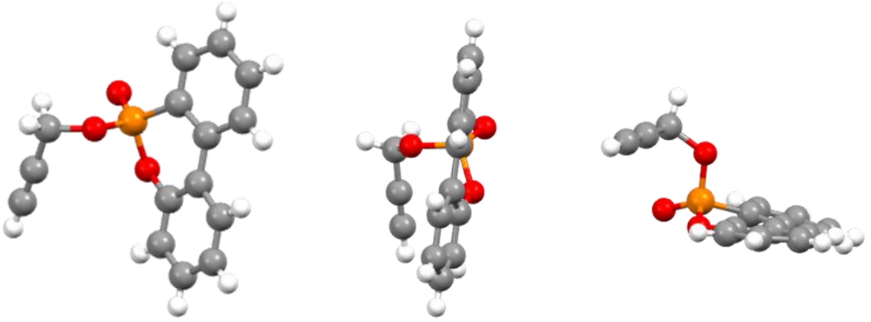

Figure S19: Crystal structure of 6-(prop-2-yn-1-yloxy)dibenzo[c,e][1,2]oxaphosphinine 6-oxide **10**

4-(1-butoxy-2-(2-methoxyphenoxy)-3-(4-(((6-oxidodibenzo[c,e][1,2]oxaphosphinin-6-yl)oxy)methyl)-1H-1,2,3-triazol-1-yl)propyl)-2-methoxyphenyl 4-methylbenzenesulfonate **11a**

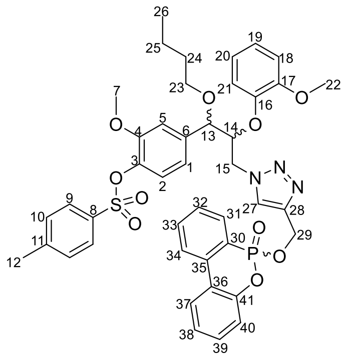

9a (173 mg, 0.31 mmol, 1.00 eq., *major/minor 1:0.7*), **10** (93.6 mg, 0.35 mmol, 1.10 eq.), sodium ascorbate (70.1 mg, 0.35 mmol, 1.10 eq.) and $CuSO_4.5H_2O$ (23.7 mg, 0.09 mmol, 0.30 eq.) were dissolved in MeOH (2.8 mL) and stirred at room temperature for 24 hours. The resulting suspension was then filtered. The filtrate was concentrated under reduced pressure, dissolved in EtOAc (15 mL), washed with sat. aq. bicarbonate solution (5

x 15 mL), dried over $MgSO_4$ and concentrated under reduced pressure. The crude product was purified by column chromatography, eluting with EtOAc/hexane (0-90%) to afford diastereomeric mixture **11a** (196.4 mg, 0.24 mmol, 77%, *major/minor 1:0.7*) as a pale yellow viscous oil. **IR** (ATR) ν_{max}/cm^{-1} 2932 (C-H), 2866 (C-H), 1593 (C-H Ar), 1501 (C-H), 1458 (C-H), 1370 (S=O asym), 1258 (P=O), 1177 (P-O-Ar), 1088 (S=O sym), 1030 (P-C Ar), 752 (P-O-C alkyl), 664 (C-S). **HRMS** (ESI) calculated for $C_{43}H_{45}N_3O_{10}PS$ $[M+H]^+$ 826.2563; found 826.2555.

6-((1-(3-butoxy-2-(2,6-dimethoxyphenoxy)-3-(3,4,5-trimethoxyphenyl)propyl)-1H-1,2,3-triazol-4-yl)methoxy)dibenzo[c,e][1,2]oxaphosphinine 6-oxide **11b**

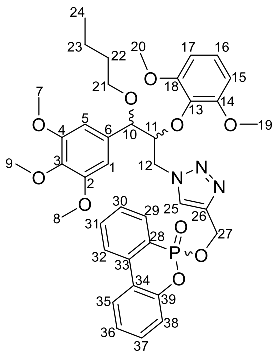

9b (105 mg, 0.22 mmol, 1.00 eq., *major/minor 1:0.6*), **10** (66.8 mg, 0.25 mmol, 1.10 eq.), sodium ascorbate (46.9 mg, 0.24 mmol, 1.10 eq.) and $CuSO_4.5H_2O$ (17.6 mg, 0.07 mmol, 0.30 eq.) were dissolved in MeOH (2.0 mL) and stirred at room temperature for 24 hours. The resulting suspension was then filtered. The filtrate was concentrated under reduced pressure, dissolved in EtOAc (15 mL), washed with sat. aq. bicarbonate solution (5 x 15 mL), dried over $MgSO_4$ and concentrated under reduced pressure. The crude product was purified by column chromatography, eluting with EtOAc/hexane (0-90%) to afford diastereomeric mixture **11b** (133.3 mg, 0.18 mmol, 81%, 4 diastereomers 1.00:0.97:0.72:0.53) as a yellow viscous oil. **IR** (ATR) ν_{max}/cm^{-1} 2940 (C-H), 28327 (C-H), 1595 (C-H Ar), 1477 (C-H), 1254 (P=O), 1105 (P-O-Ar), 1038 (P-C Ar), 758 (P-O-C alkyl). **HRMS** (ESI) calculated for $C_{39}H_{44}N_3O_{10}PNa$ $[M+Na]^+$ 768.2662; found 768.2635.

6-((1-(3-butoxy-3-(3,4-dimethoxyphenyl)-2-(2-methoxyphenoxy)propyl)-1H-1,2,3-triazol-4-yl)methoxy)dibenzo[c,e][1,2]oxaphosphinine 6-oxide **11c**

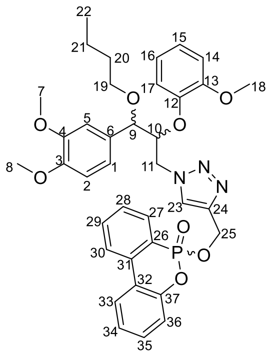

9c (103 mg, 0.25 mmol, 1.00 eq., *major/minor 1:0.2*), **10** (73.3 mg, 0.27 mmol, 1.10 eq.), sodium ascorbate (55.3 mg, 0.28 mmol, 1.10 eq.) and $CuSO_4.5H_2O$ (19.4 mg, 0.08 mmol, 0.30 eq.) were dissolved in MeOH (2.0 mL) and stirred at room temperature for 24 hours. The resulting suspension was then filtered. The filtrate was concentrated under reduced pressure, dissolved in EtOAc (15 mL), washed with sat. aq. bicarbonate solution (5 x 15 mL), dried over $MgSO_4$ and concentrated under reduced pressure. The crude product was purified by column chromatography, eluting with EtOAc/hexane (0-90%) to afford diastereomeric mixture **11c** (144 mg, 0.21 mmol, 85%, *major/minor 1:0.7*) as a yellow viscous oil. **IR** (ATR) ν_{max}/cm^{-1} 2951 (C-H), 2870 (C-H), 1598 (C-H Ar), 1501 (C-H), 1450 (C-H), 1254 (P=O), 1119 (P-O-Ar), 1025 (P-C Ar), 752 (P-O-C alkyl). **HRMS** (ESI) calculated for $C_{37}H_{40}N_3O_8PNa$ $[M+Na]^+$ 708.2451; found 708.2431.

3-butoxy-2-(2,6-dimethoxyphenoxy)-3-(3,4,5-trimethoxyphenyl)propyl benzoate **S1a**

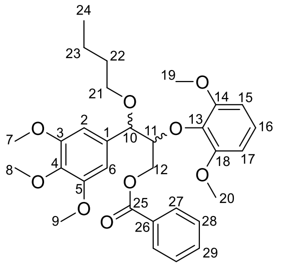

5b (106 mg, 0.24 mmol, 1.00 eq., *major/minor 1:0.5*) and benzoic anhydride (153 mg, 0.68 mmol, 3.00 eq.) were dissolved in 4:1 DMSO/*N*-methylimidazole (3.0 mL) and stirred at room temperature for 18 hours. The solution was diluted with EtOAc (10 mL), washed with water (3 x 10.0 mL) then brine (1 x 10.0 mL), dried over $MgSO_4$ and concentrated under reduced pressure. The crude product was purified by column chromatography, eluting with EtOAc/hexane (0-50%) to afford **S1a** (98.9 mg, 0.18 mmol, 76%, *major/minor 1:0.6*) as a pale yellow viscous oil. **IR** (ATR) ν_{max}/cm^{-1} 2940 (C-H), 1718 (C=O), 1593 (C-H Ar), 1477 (C-H), 1254 (C-O ether), 1109 (C-O ether), 1005 (C-O ether). **HRMS** (ESI) calculated for $C_{31}H_{38}O_9Na$ $[M+Na]^+$ 577.2414; found 577.2401.

3-butoxy-3-(3,4-dimethoxyphenyl)-2-(2-methoxyphenoxy)propyl benzoate **S1b**

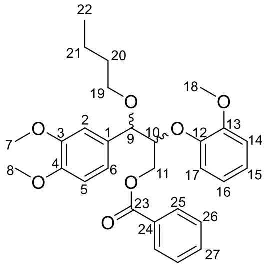

5c (102 mg, 0.26 mmol, 1.00 eq., *major/minor 1:0.8*) and benzoic anhydride (176 mg, 0.0.78 mmol, 3.00 eq.) were dissolved in 4:1 DMSO/*N*-methylimidazole (3.0 mL) and stirred at room temperature for 18 hours. The solution was diluted with EtOAc (10 mL), washed with water (3 x 10.0 mL) then brine (1 x 10.0 mL), dried over $MgSO_4$ and concentrated under reduced pressure. The crude product was purified by column chromatography, eluting with EtOAc/hexane (0-50%) to afford **S1b** (117.3 mg, 0.24 mmol, 91%, *major/minor 1:0.7*) as a pale yellow viscous oil. **IR** (ATR) ν_{max}/cm^{-1} 2960 (C-H), 1718 (C=O), 1591 (C-H Ar), 1499 (C-H), 1450 (C-H), 1254 (C-O ether), 1097 (C-O ether), 1024 (C-O ether). **HRMS** (ESI) calculated for $C_{29}H_{34}O_7Na$ $[M+Na]^+$ 517.2202; found 517.2189.

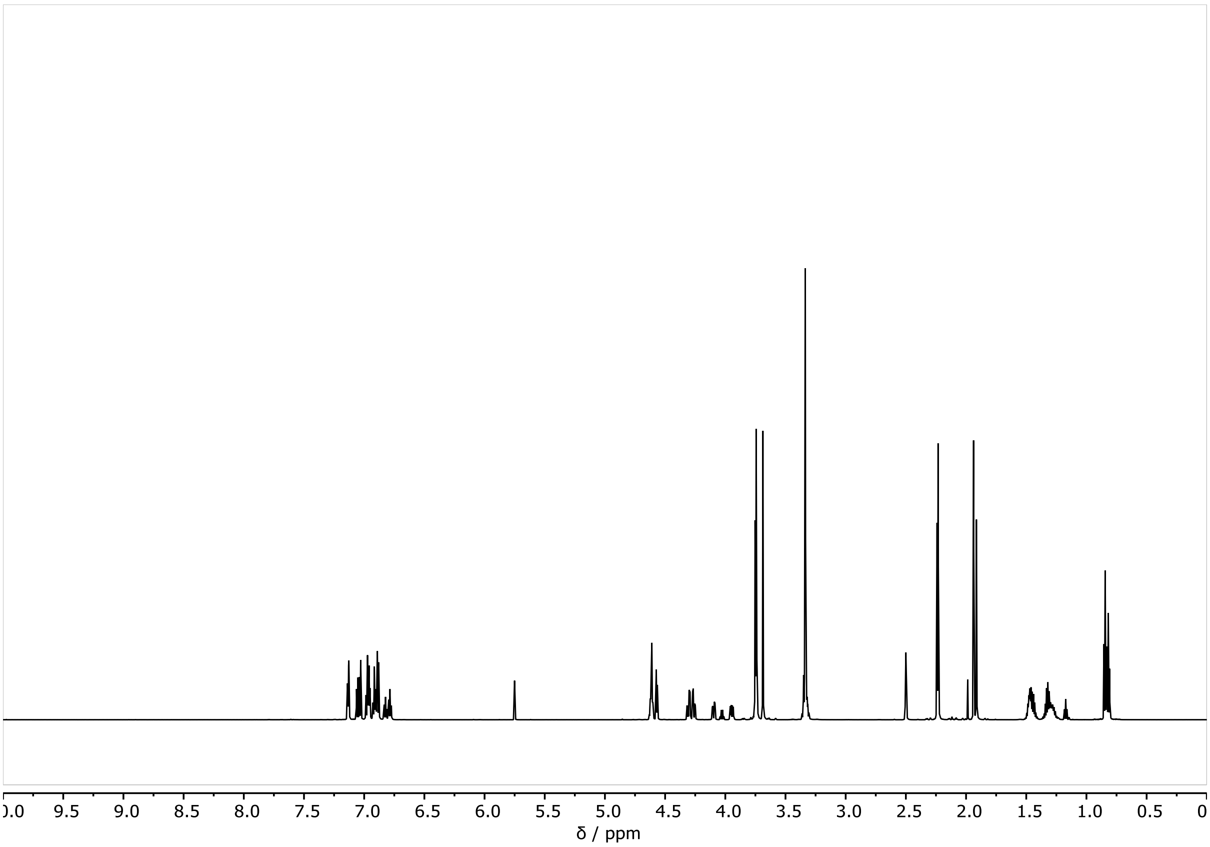

Figure S20: ^{1}H NMR (700 MHz, DMSO-d$_6$) of model compound **3a**

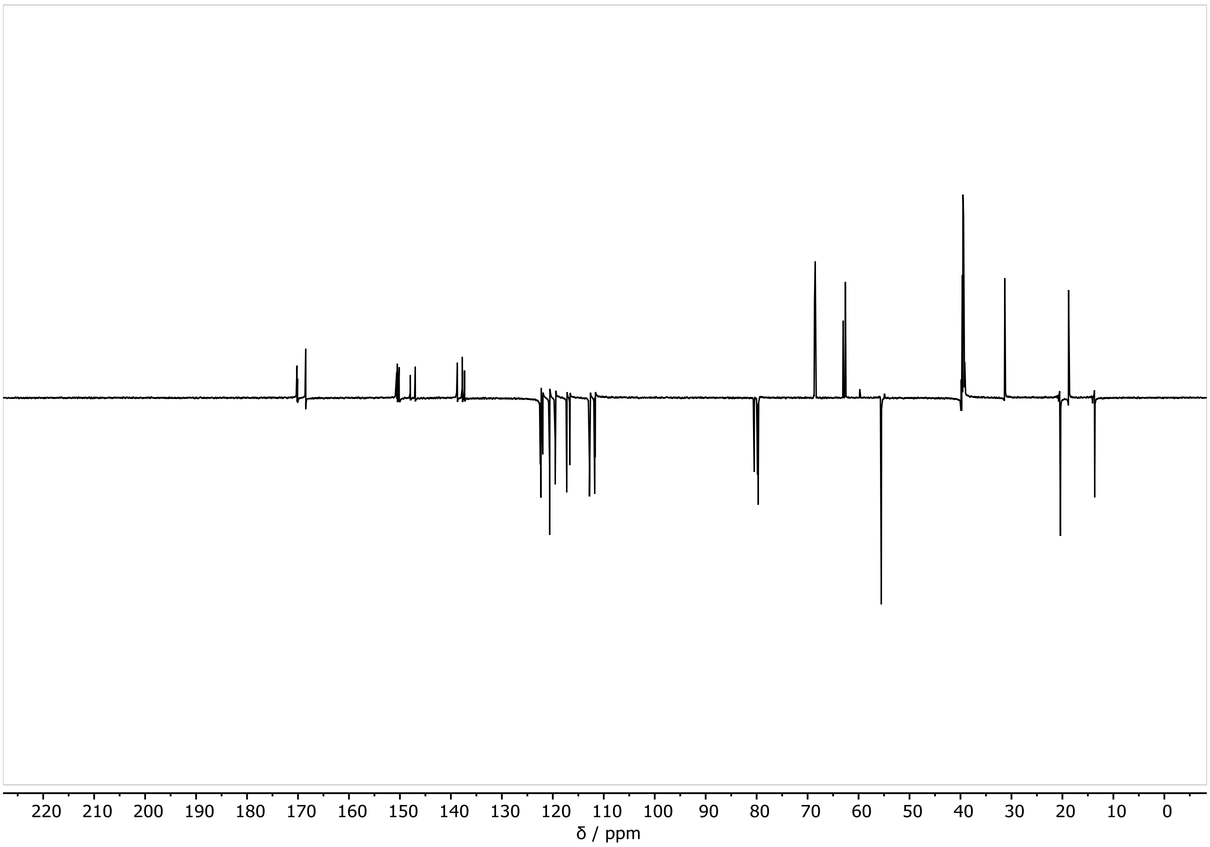

Figure S21: ^{13}C NMR (176 MHz, DMSO-d$_6$) of model compound **3a**

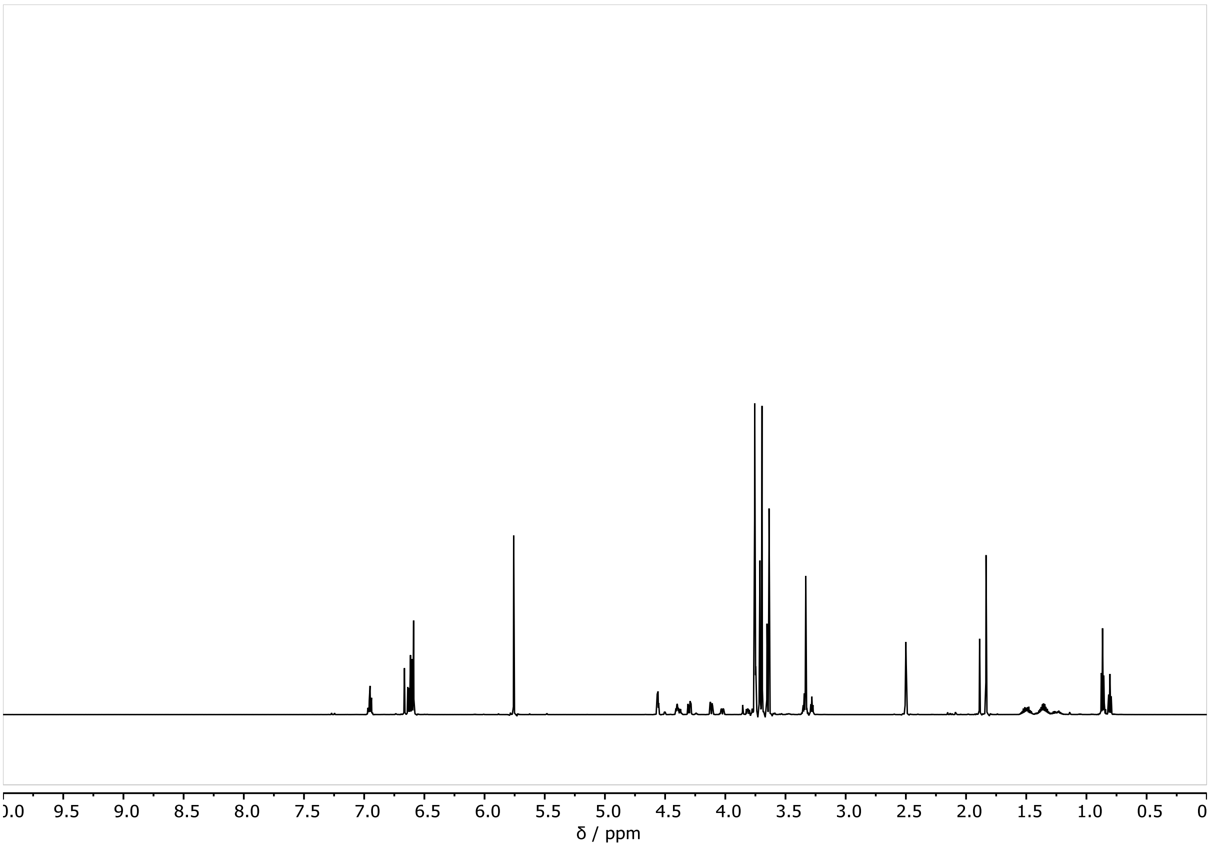

Figure S22: ^{1}H NMR (700 MHz, DMSO-d$_6$) of model compound **3b**

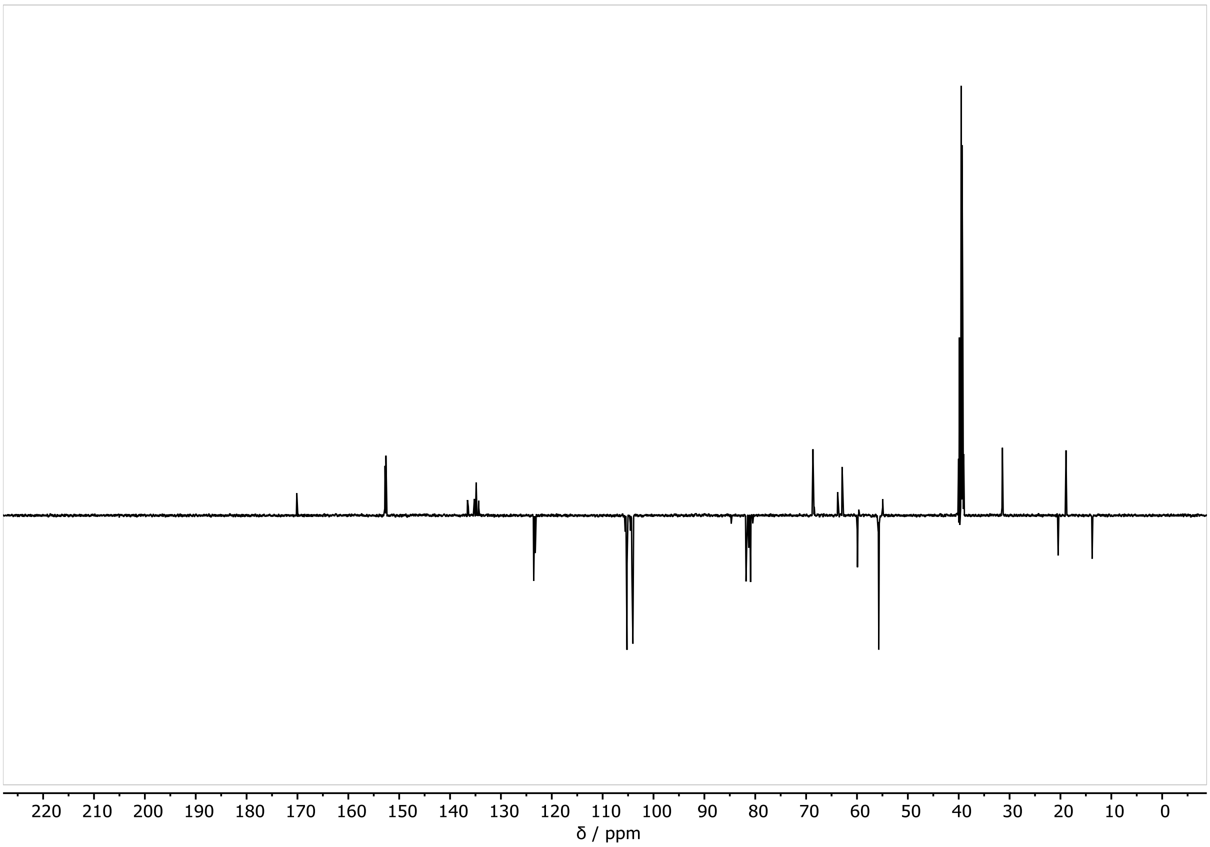

Figure S23: ^{13}C NMR (176 MHz, DMSO-d$_6$) of model compound **3b**

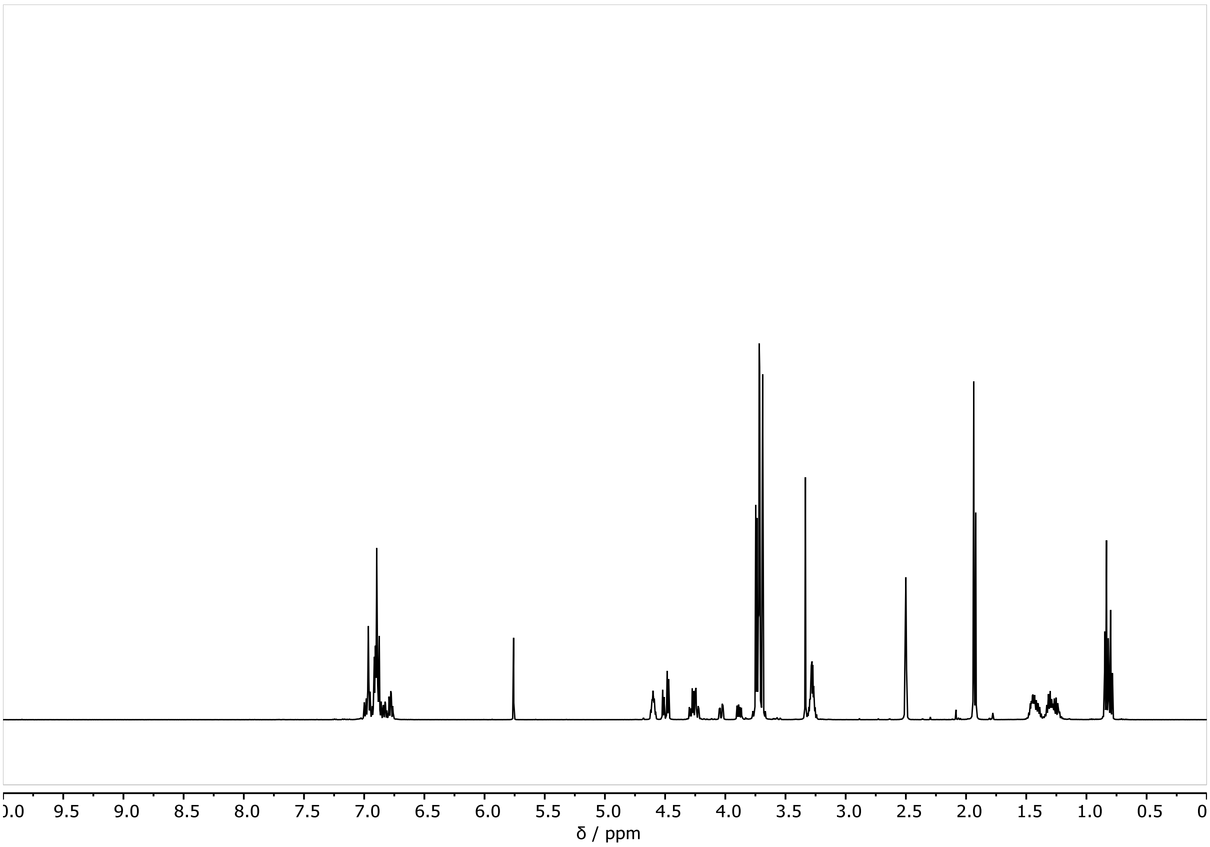

Figure S24: ^{1}H NMR (500 MHz, DMSO-d$_6$) of model compound **3c**

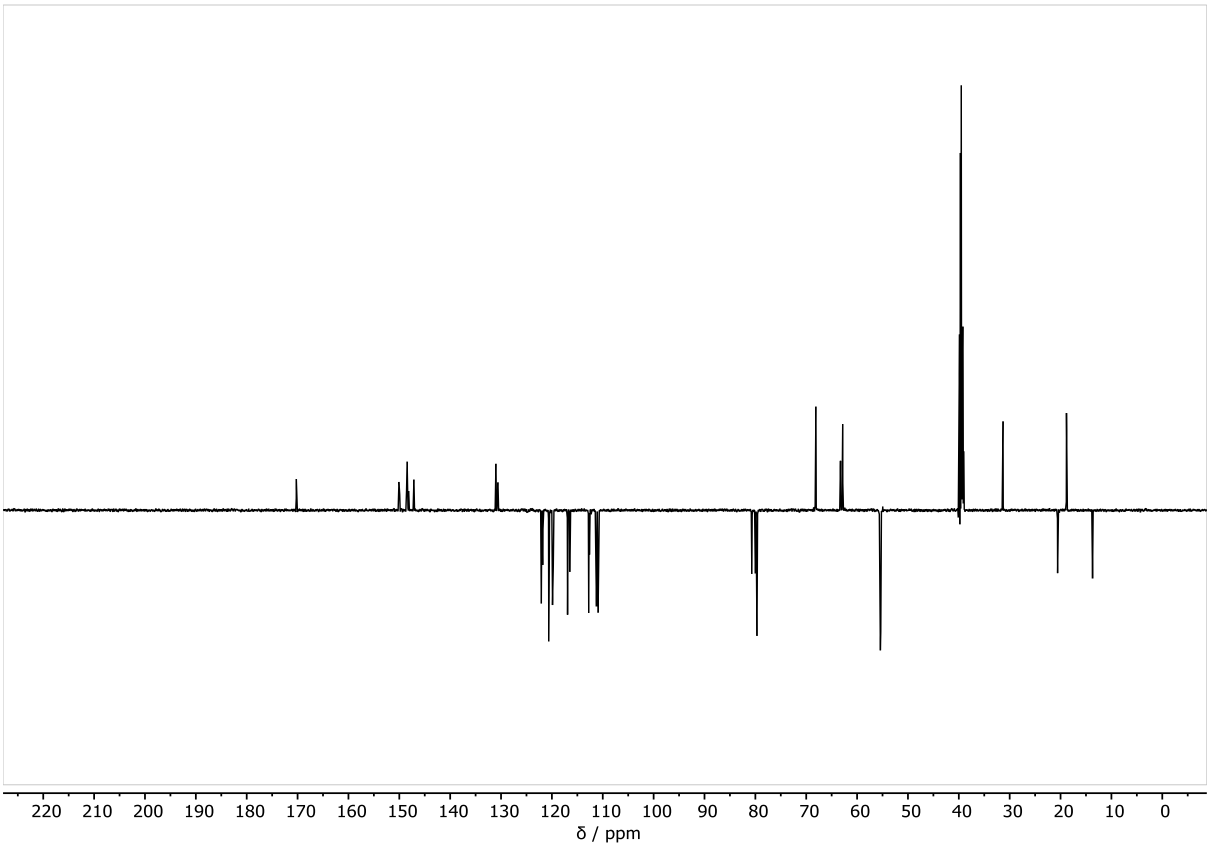

Figure S25: ^{13}C NMR (176 MHz, DMSO-d$_6$) of model compound **3c**

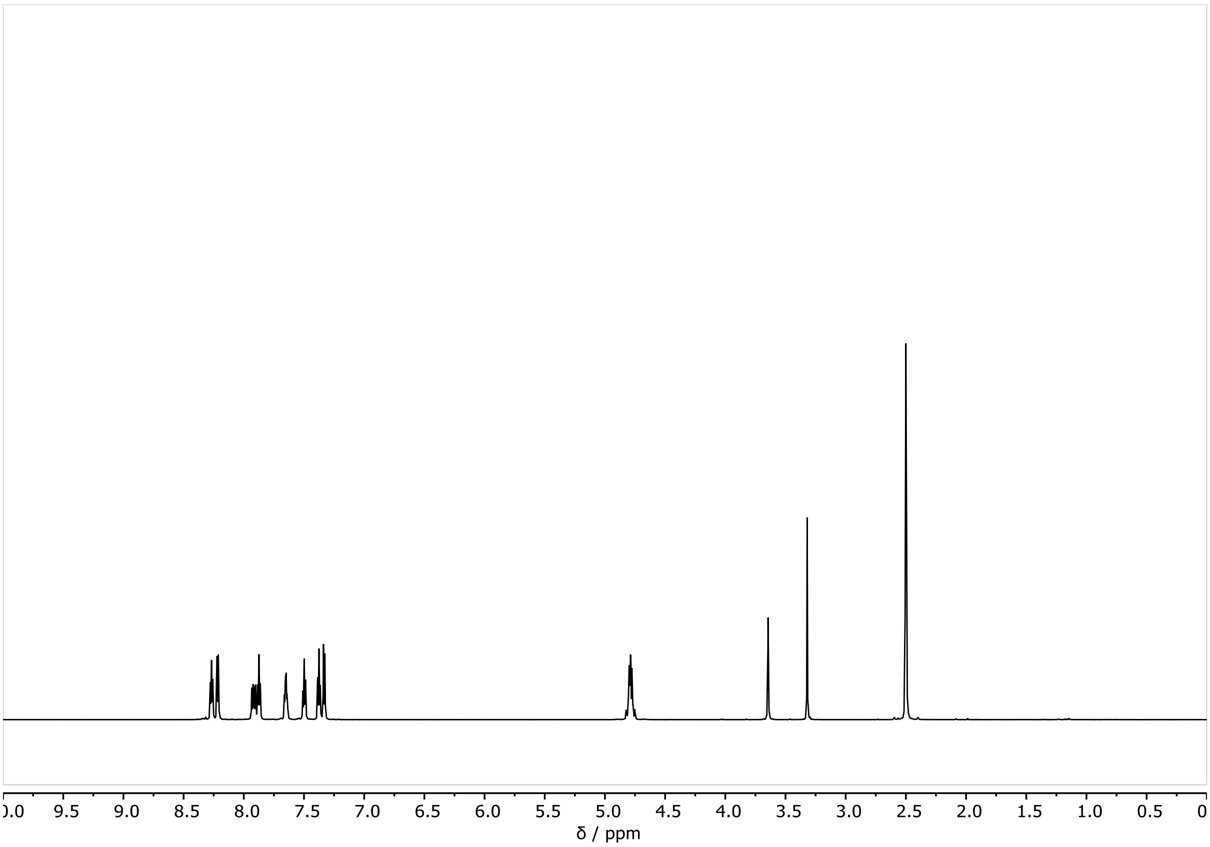

Figure S26: ^{1}H NMR (700 MHz, DMSO-d_6) of model compound **10**

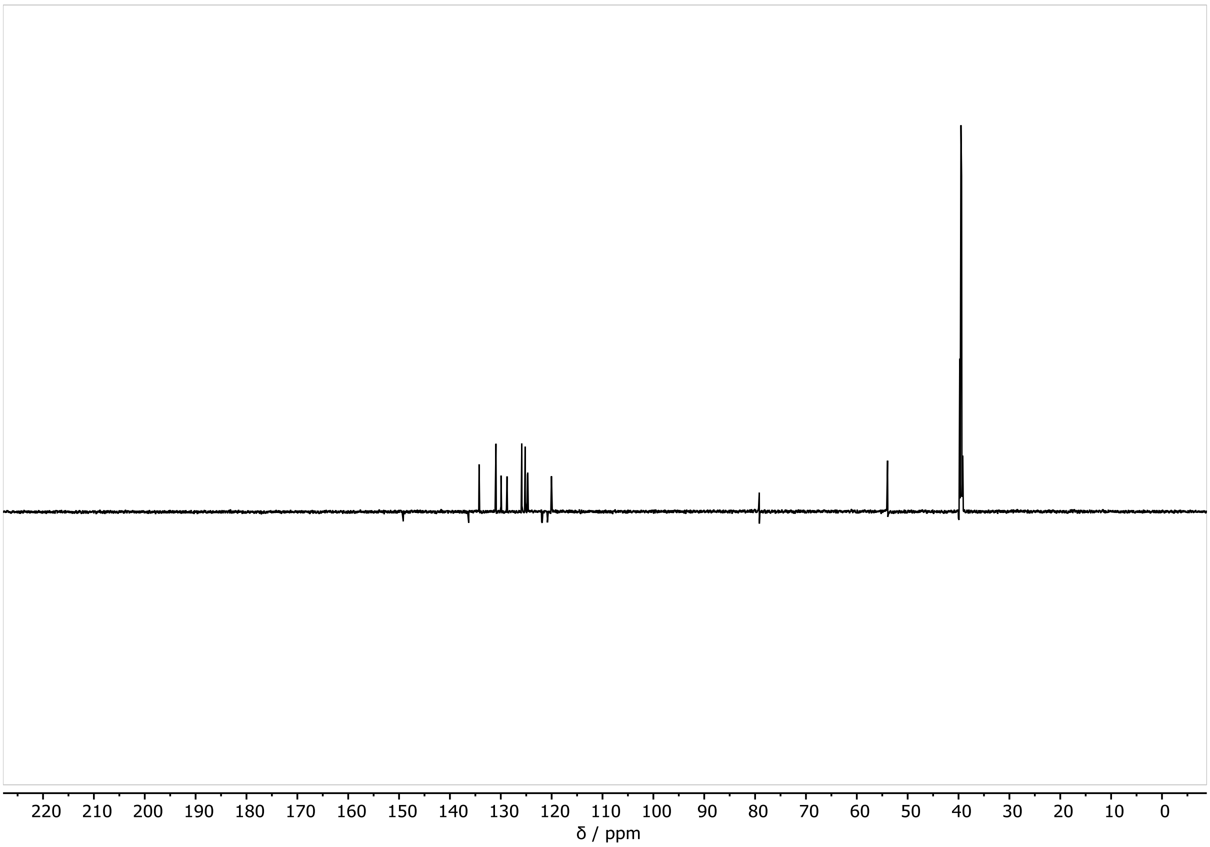

Figure S27: ^{13}C NMR (176 MHz, DMSO-d_6) of model compound **10**

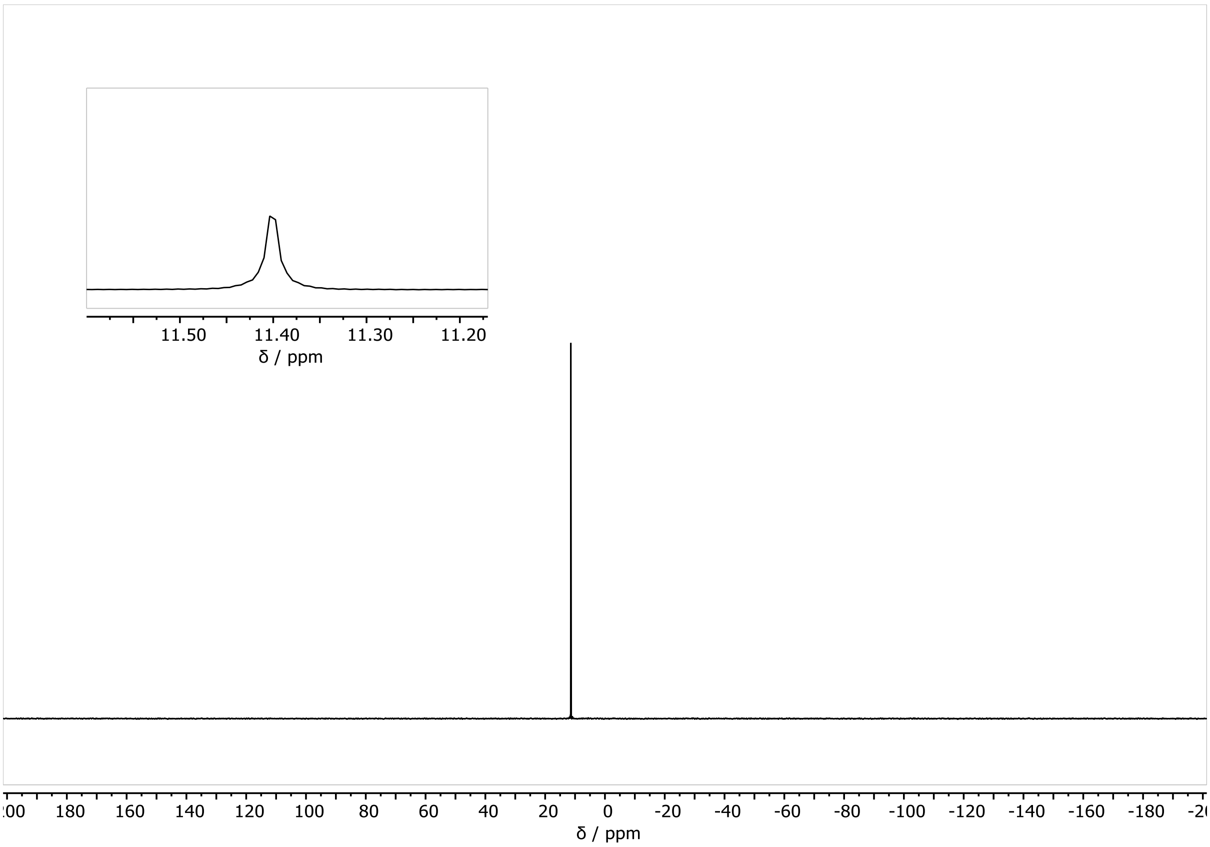

Figure S28: ^{31}P NMR (202 MHz, DMSO-d_6) of model compound **10**

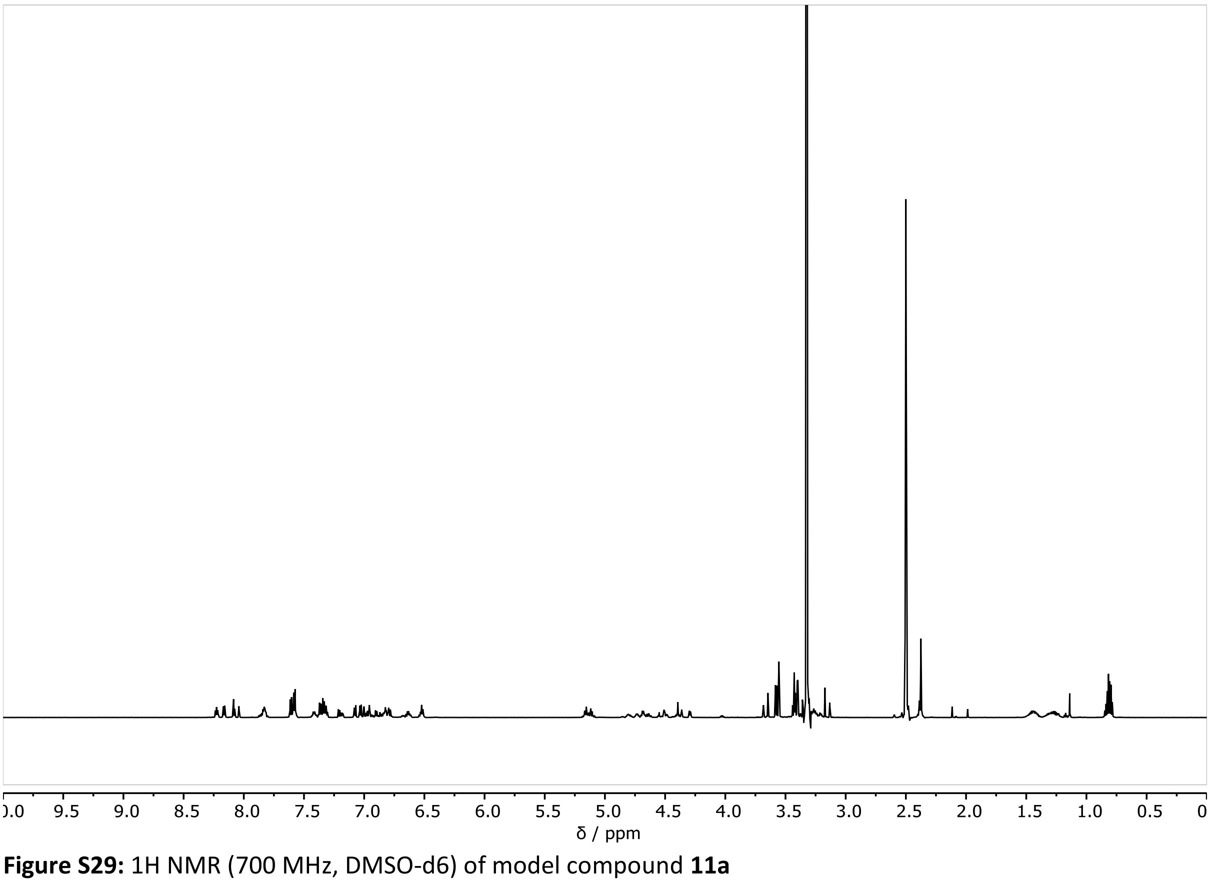

Figure S29: 1H NMR (700 MHz, DMSO-d6) of model compound **11a**

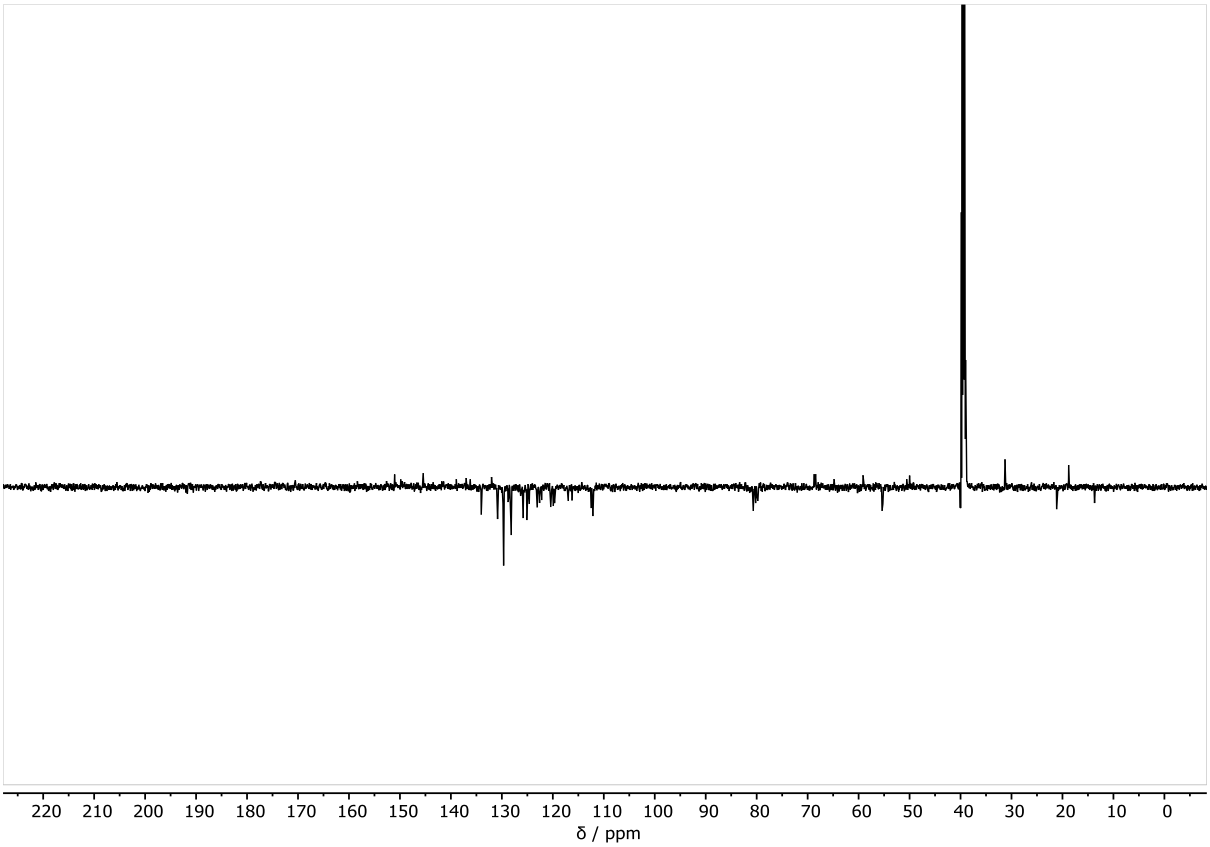

Figure S30: ^{13}C NMR (176 MHz, DMSO-d$_6$) of model compound **11a**

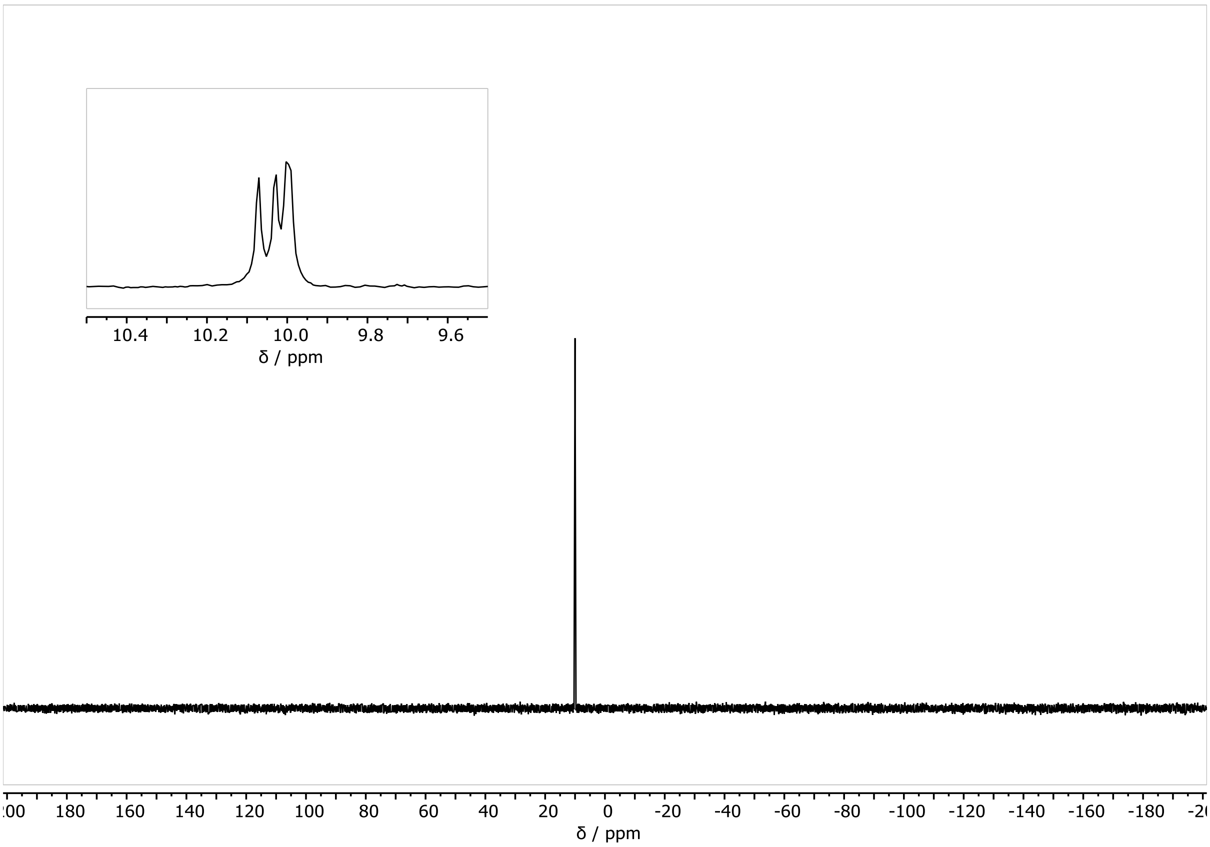

Figure S31: ^{31}P NMR (202 MHz, DMSO-d$_6$) of model compound **11a**

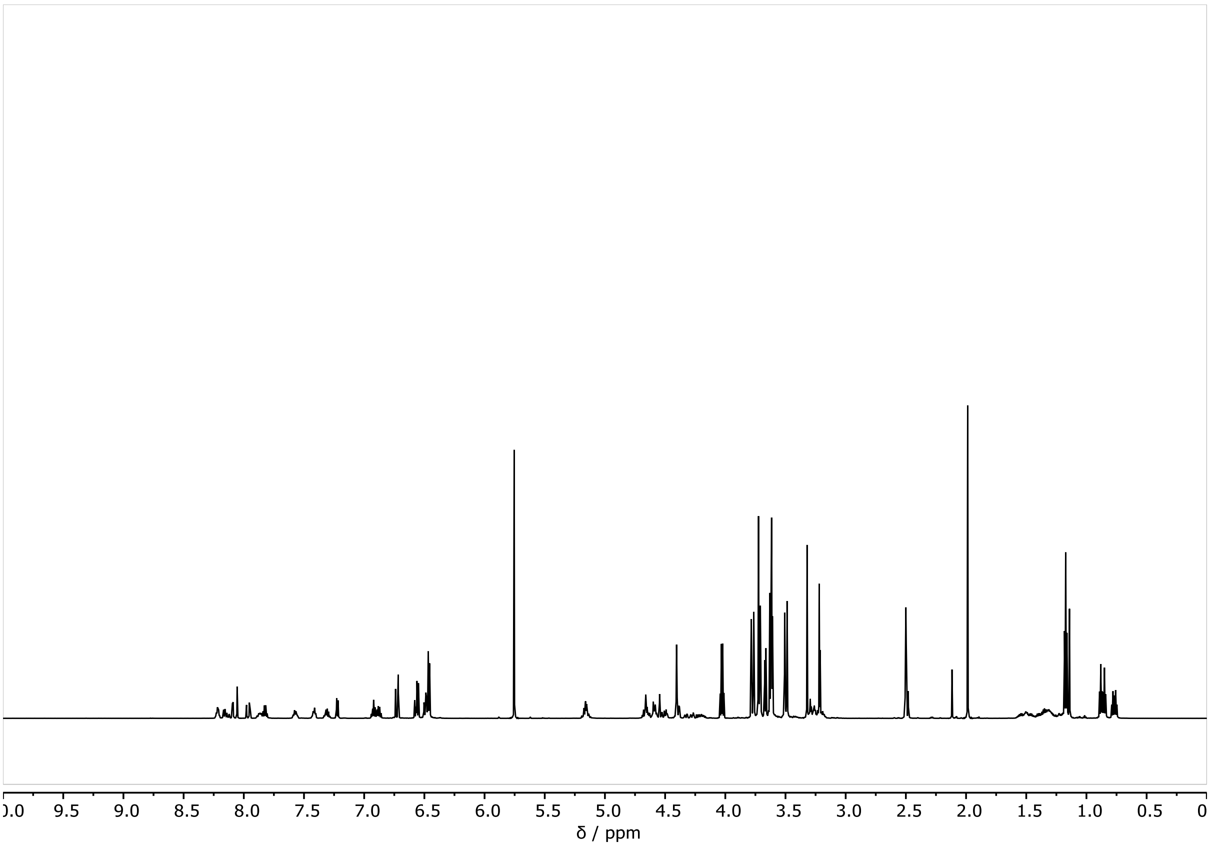

Figure S32: ^{1}H NMR (700 MHz, DMSO-d_6) of model compound **11b**

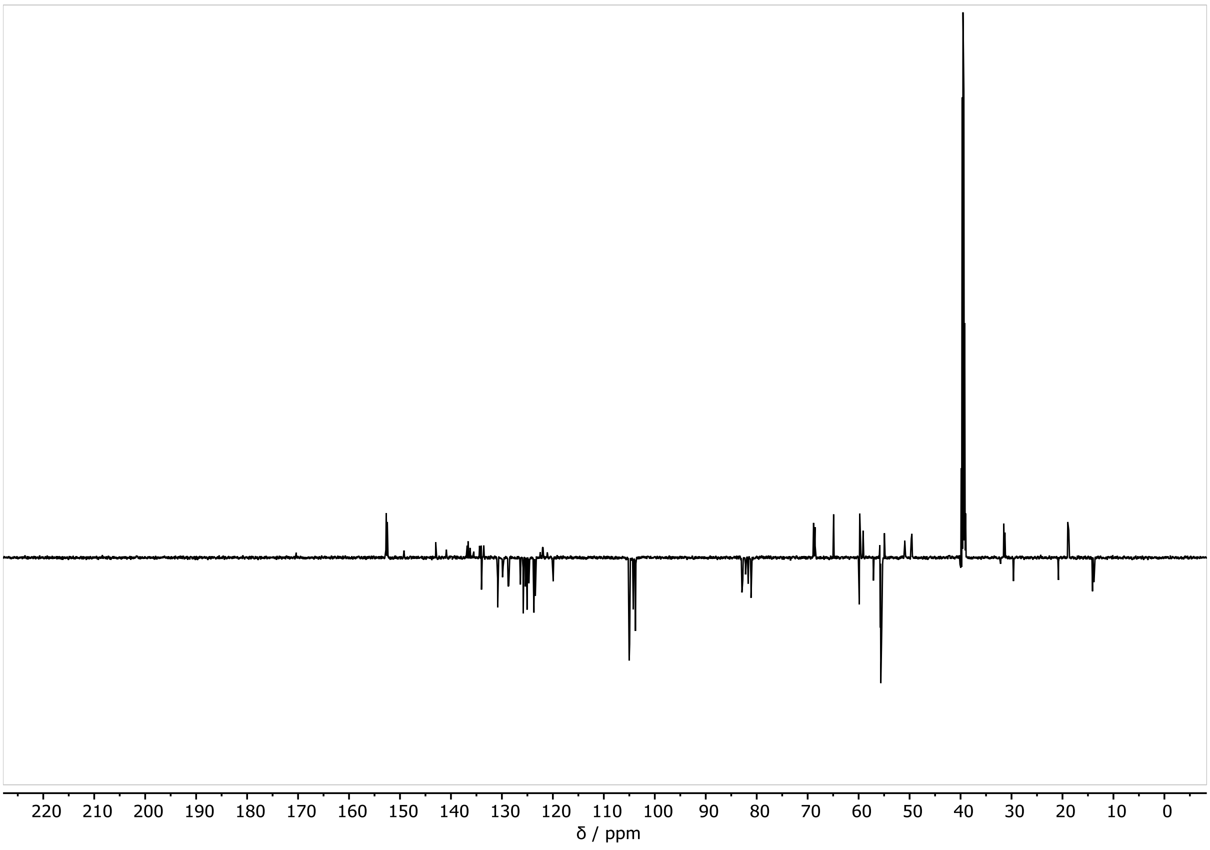

Figure S33: ^{13}C NMR (176 MHz, DMSO-d_6) of model compound **11b**

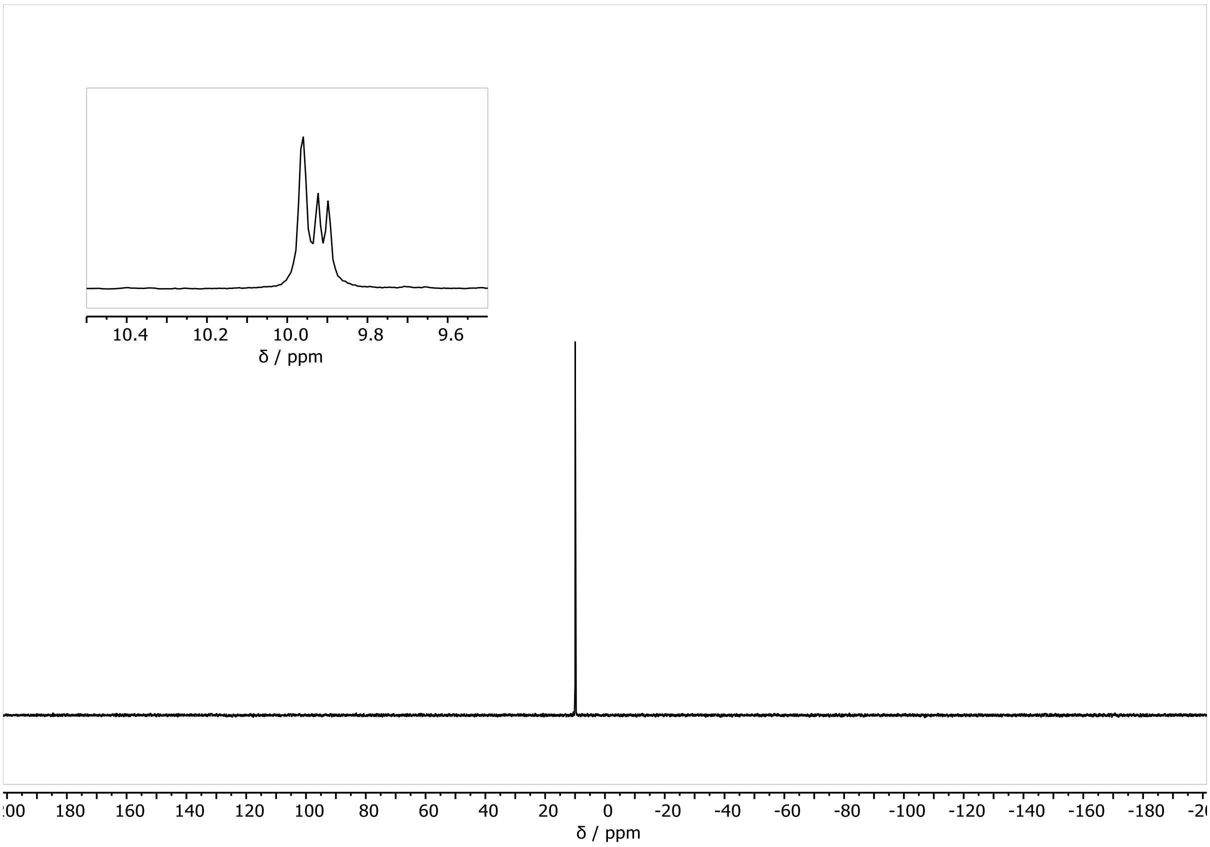

Figure S34: ^{31}P NMR (202 MHz, DMSO-d$_6$) of model compound **11b**

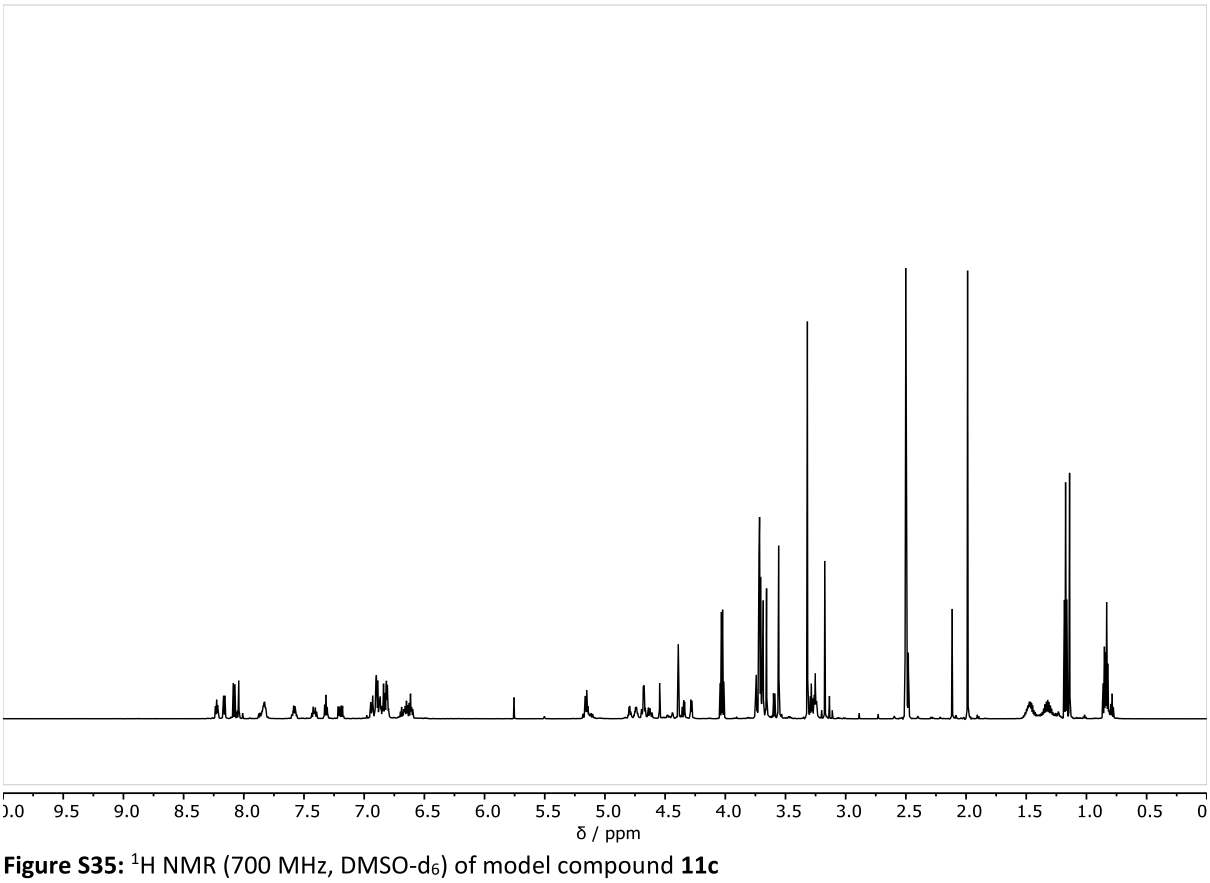

Figure S35: ^{1}H NMR (700 MHz, DMSO-d$_6$) of model compound **11c**

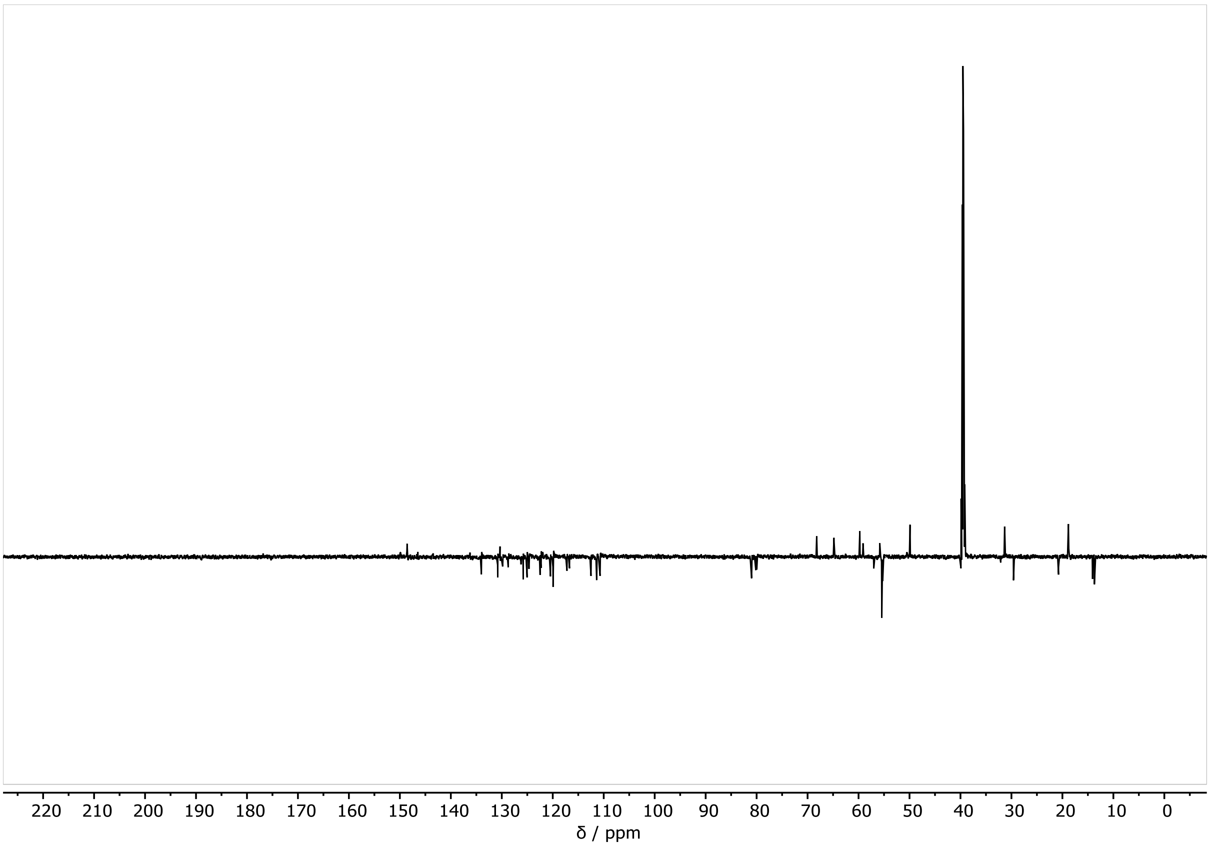

Figure S36: ^{13}C NMR (176 MHz, DMSO-d_6) of model compound **11c**

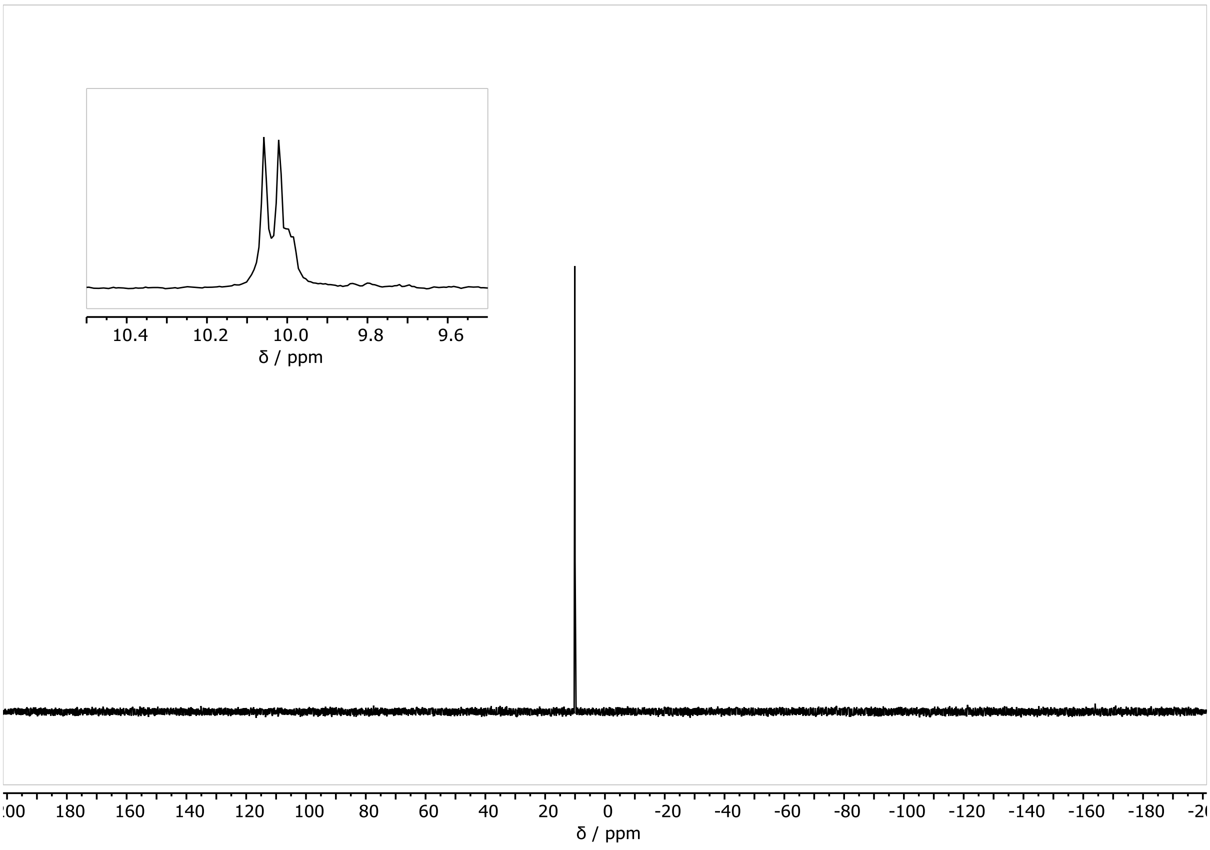

Figure S37: ^{31}P NMR (202 MHz, DMSO-d_6) of model compound **11c**

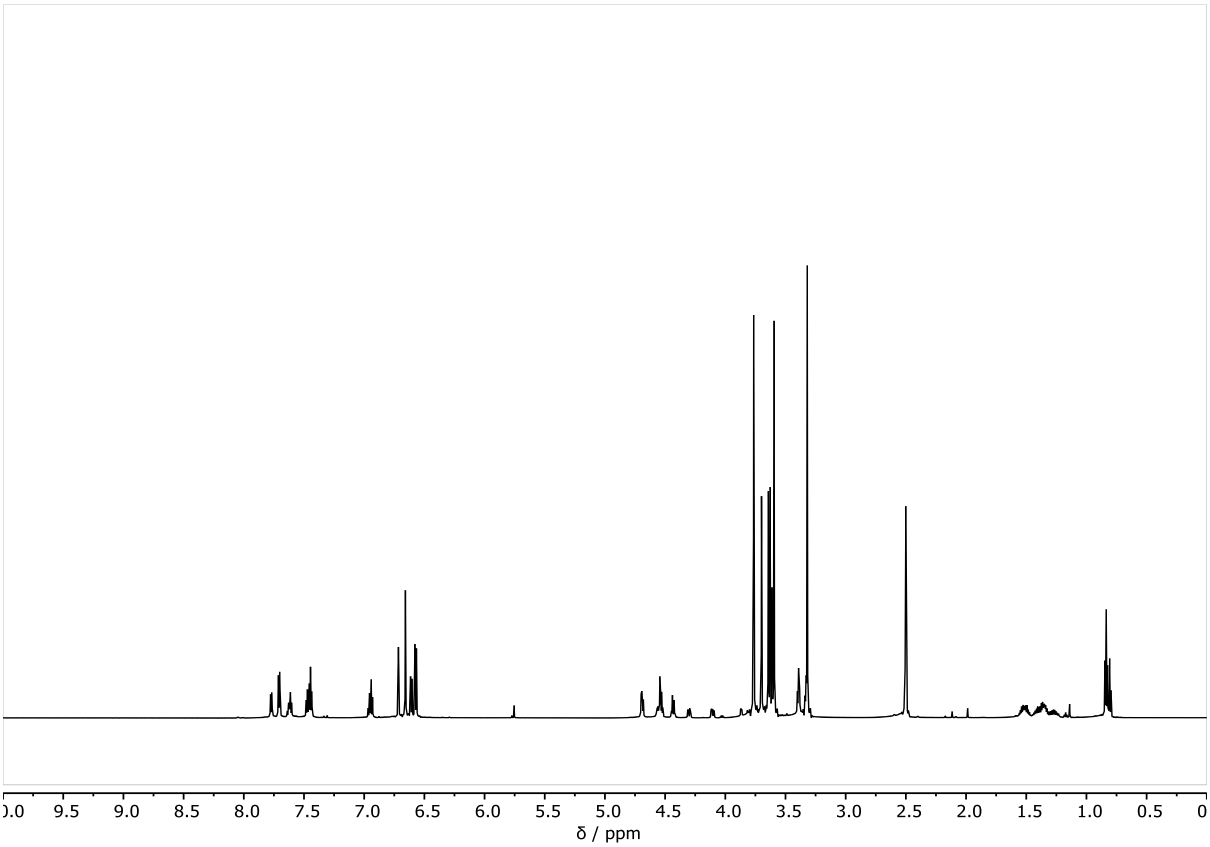

Figure S38: ^{1}H NMR (700 MHz, DMSO-d$_6$) of model compound **S1a**

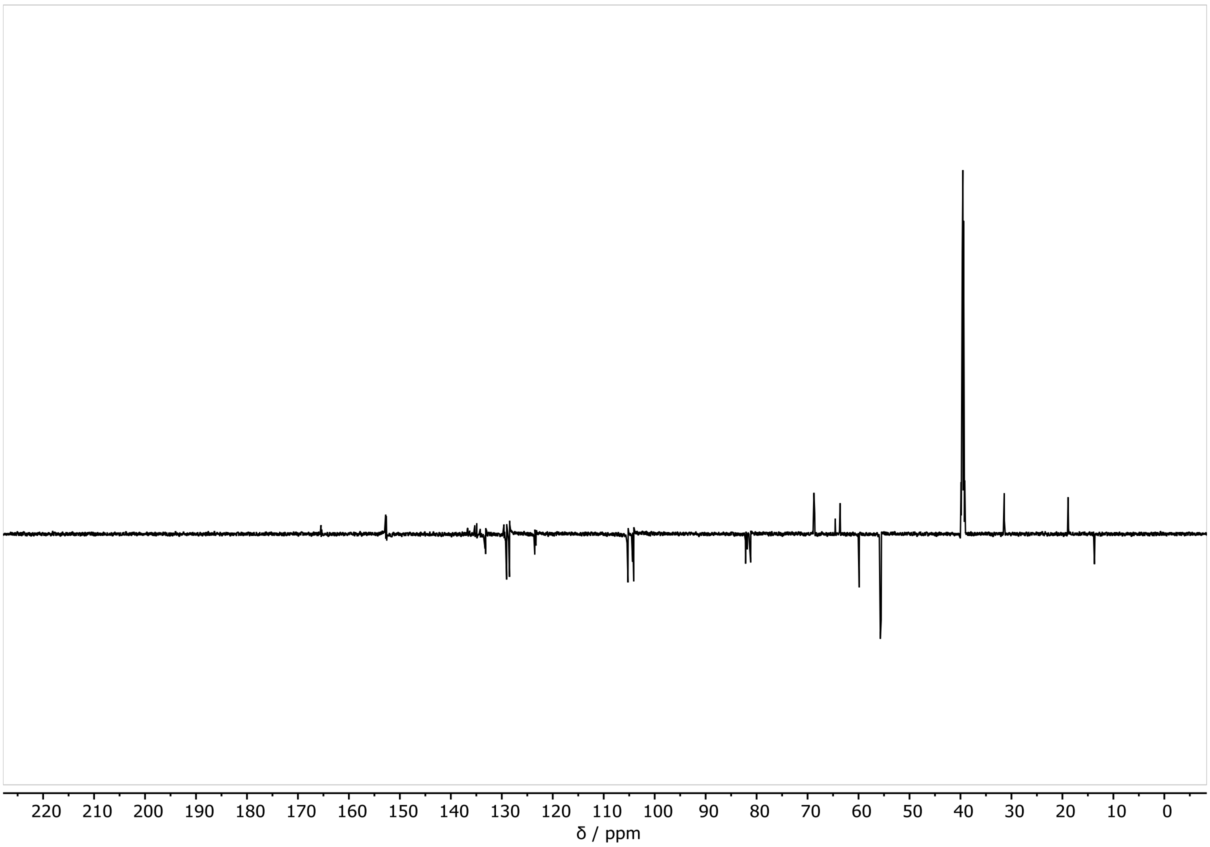

Figure S39: ^{13}C NMR (176 MHz, DMSO-d$_6$) of model compound **S1a**

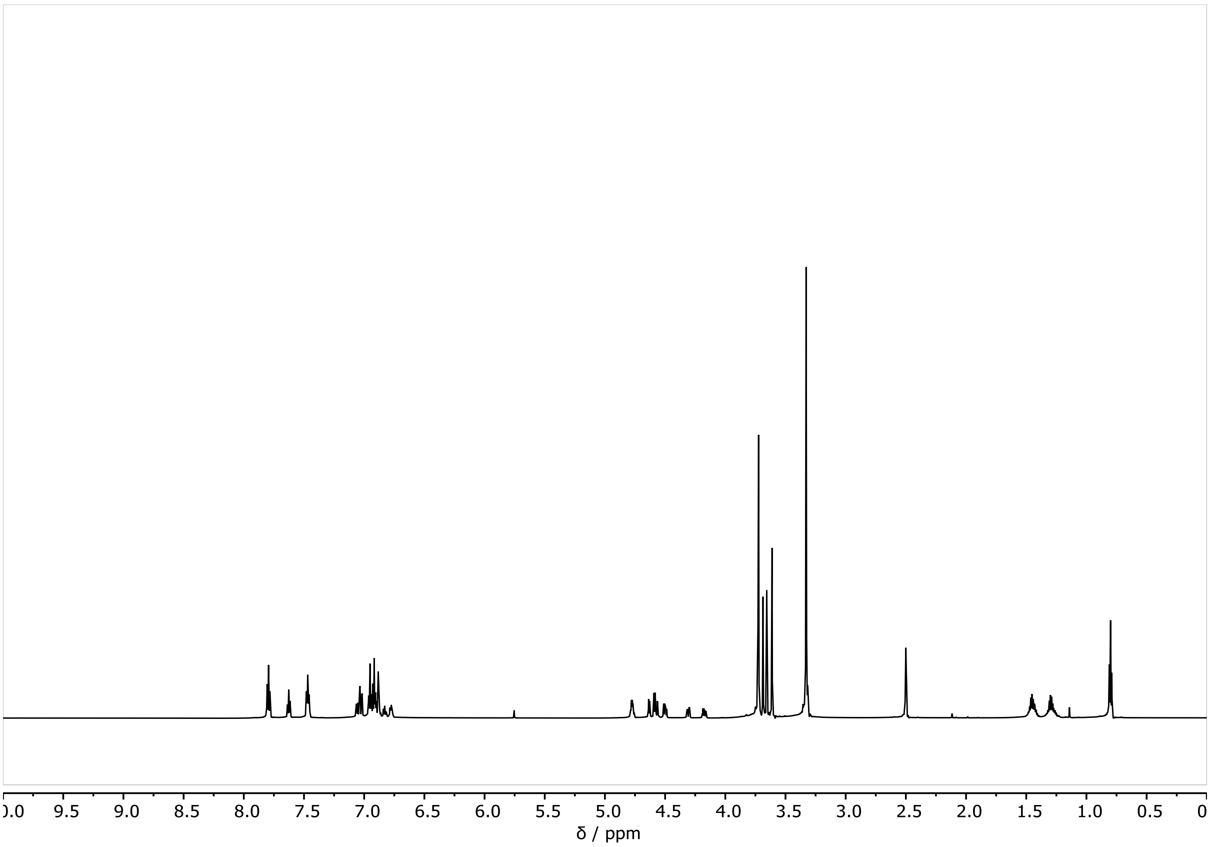

Figure S40: ^{1}H NMR (700 MHz, DMSO-d$_6$) of model compound **S1b**

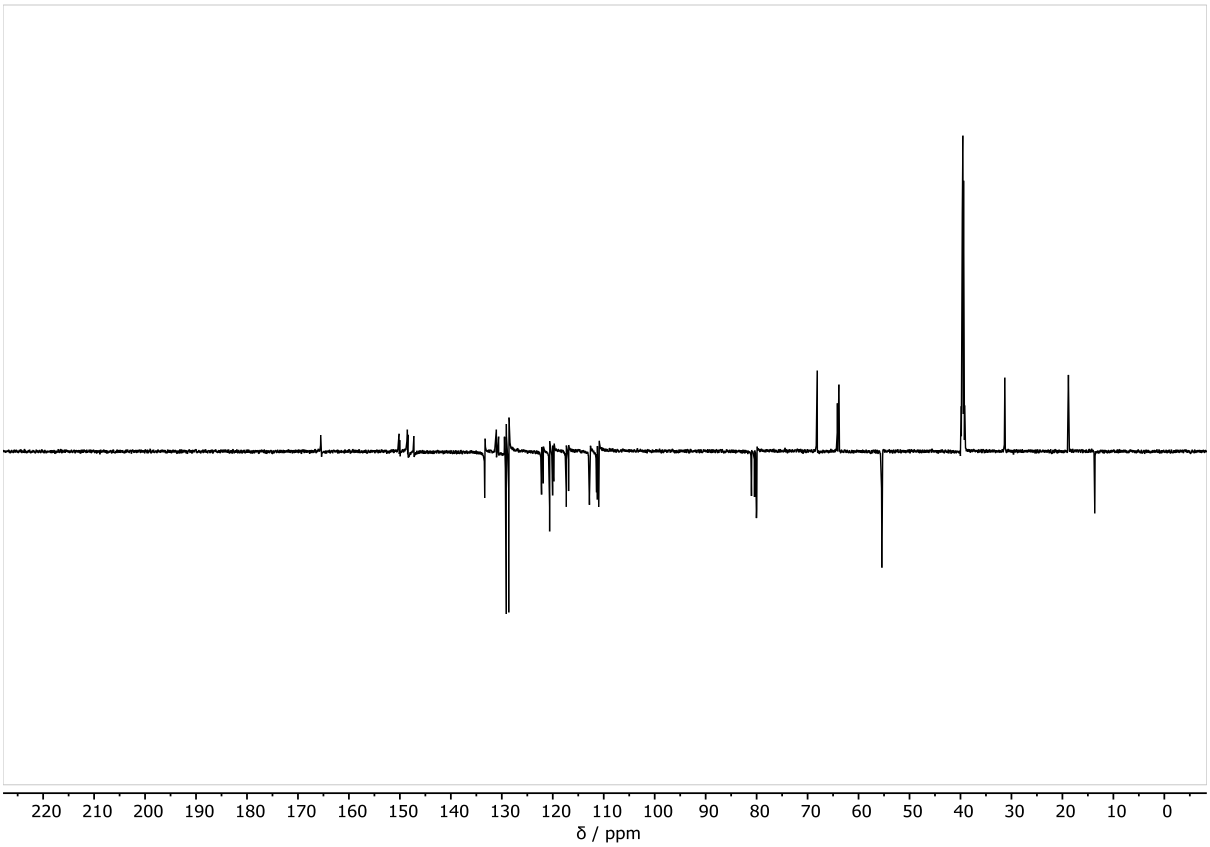

Figure S41: ^{13}C NMR (176 MHz, DMSO-d$_6$) of model compound **S1b**

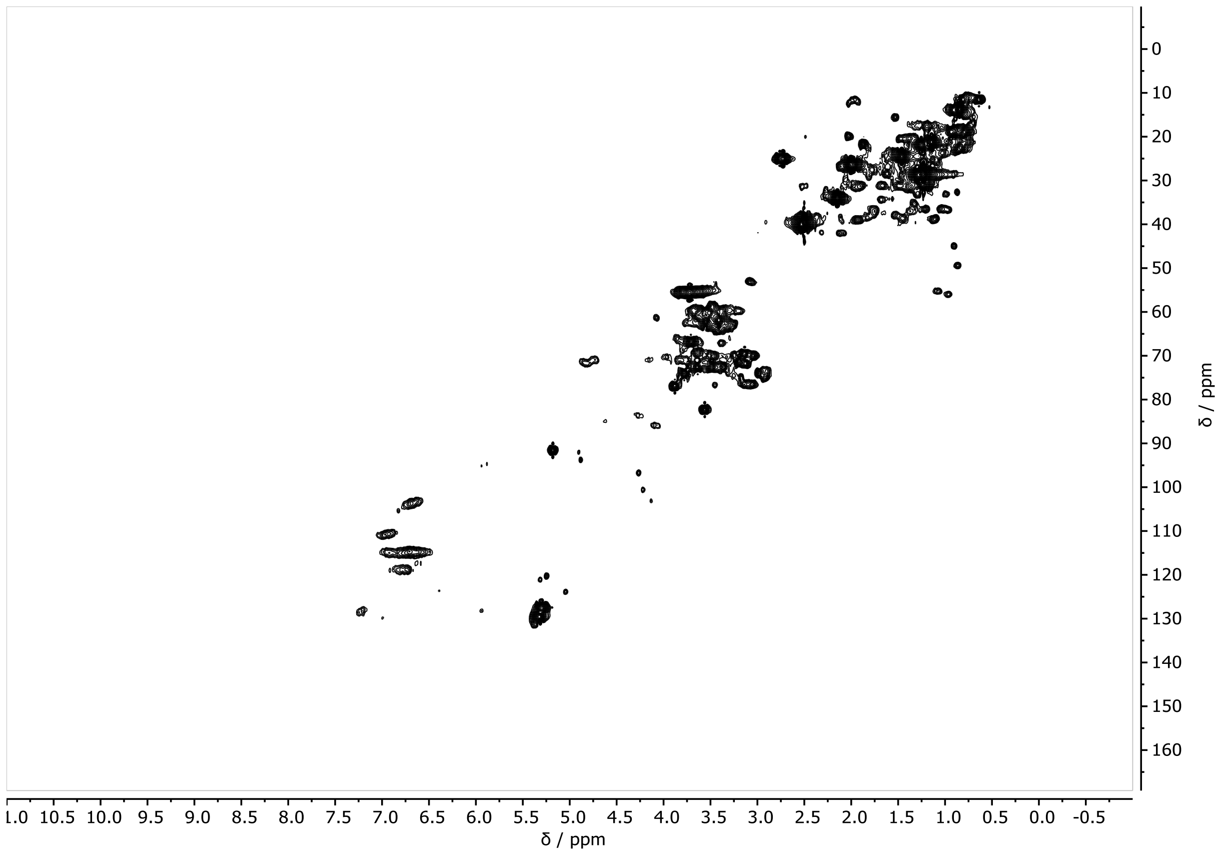

Figure S42: HSQC NMR (700 MHz, DMSO-d$_6$) of CPH 0.5M alkali lignin (Figure S1)

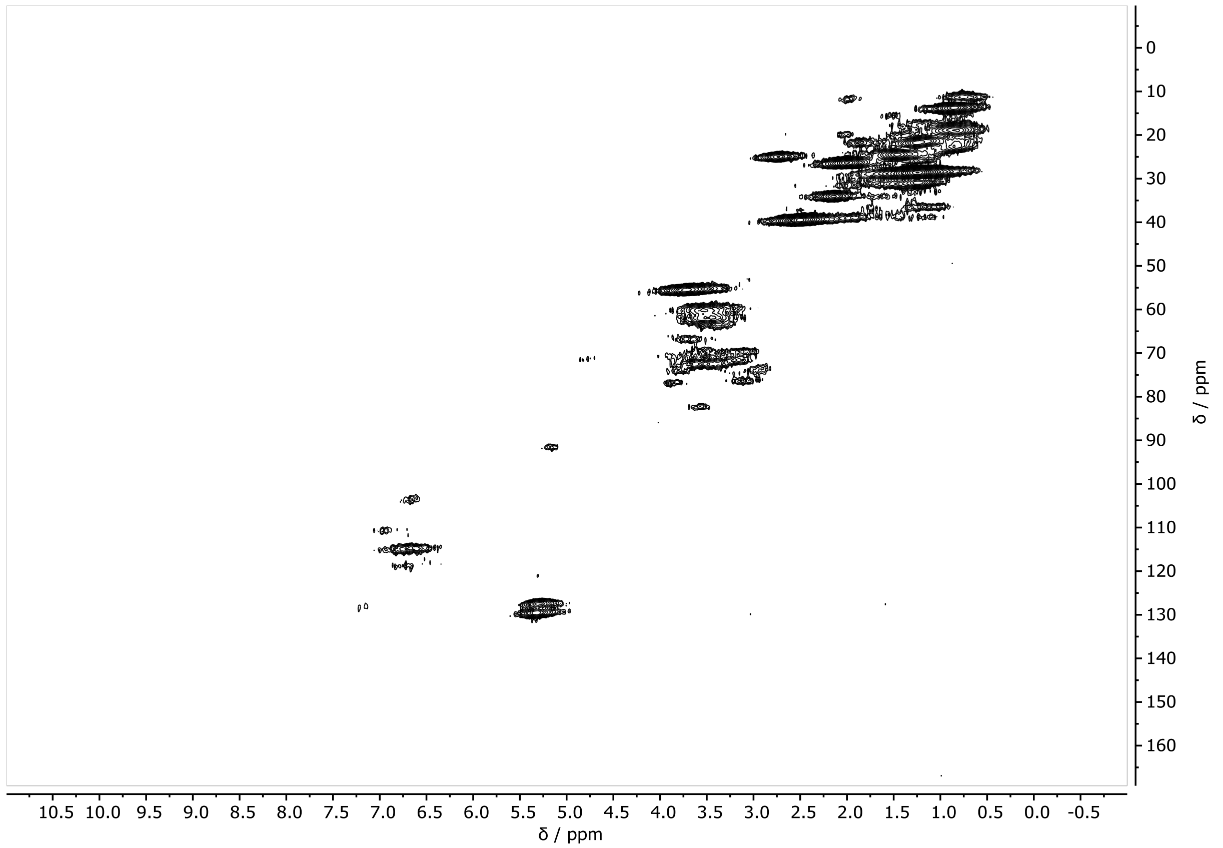

Figure S43: HSQC NMR (700 MHz, DMSO-d$_6$) of CPH 1.5M alkali lignin (Figure S1)

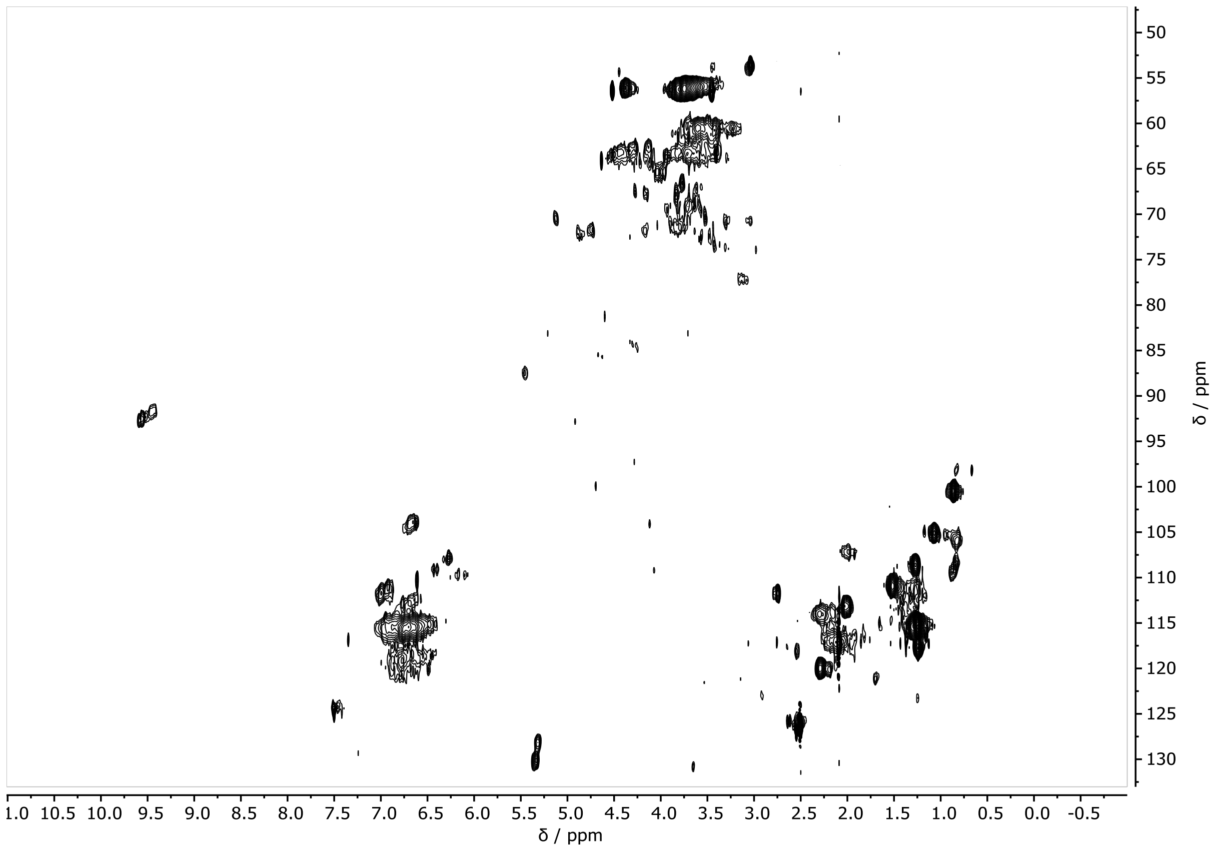

Figure S44: HSQC NMR (700 MHz, DMSO-d_6) of CPH dioxasolv lignin.

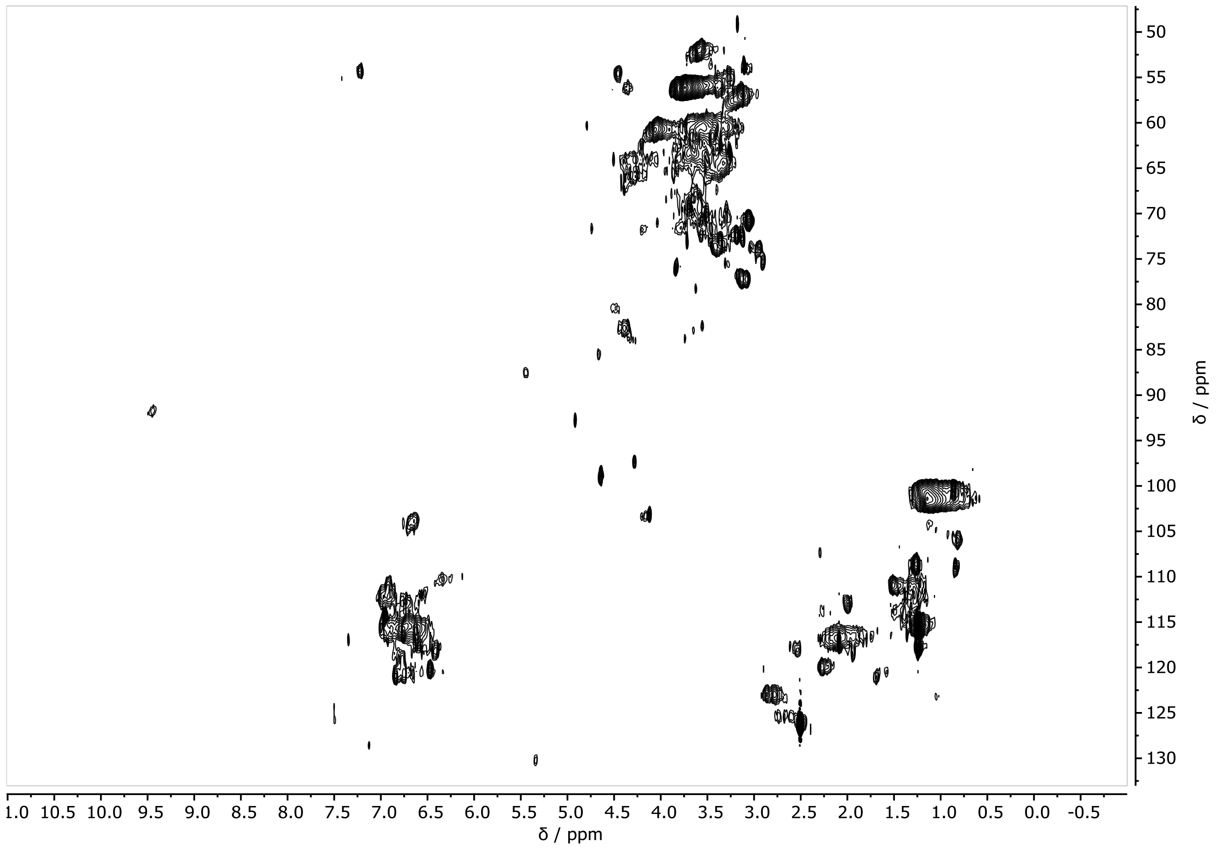

Figure S45: HSQC NMR (700 MHz, DMSO-d_6) of CPH ethanosolv lignin.

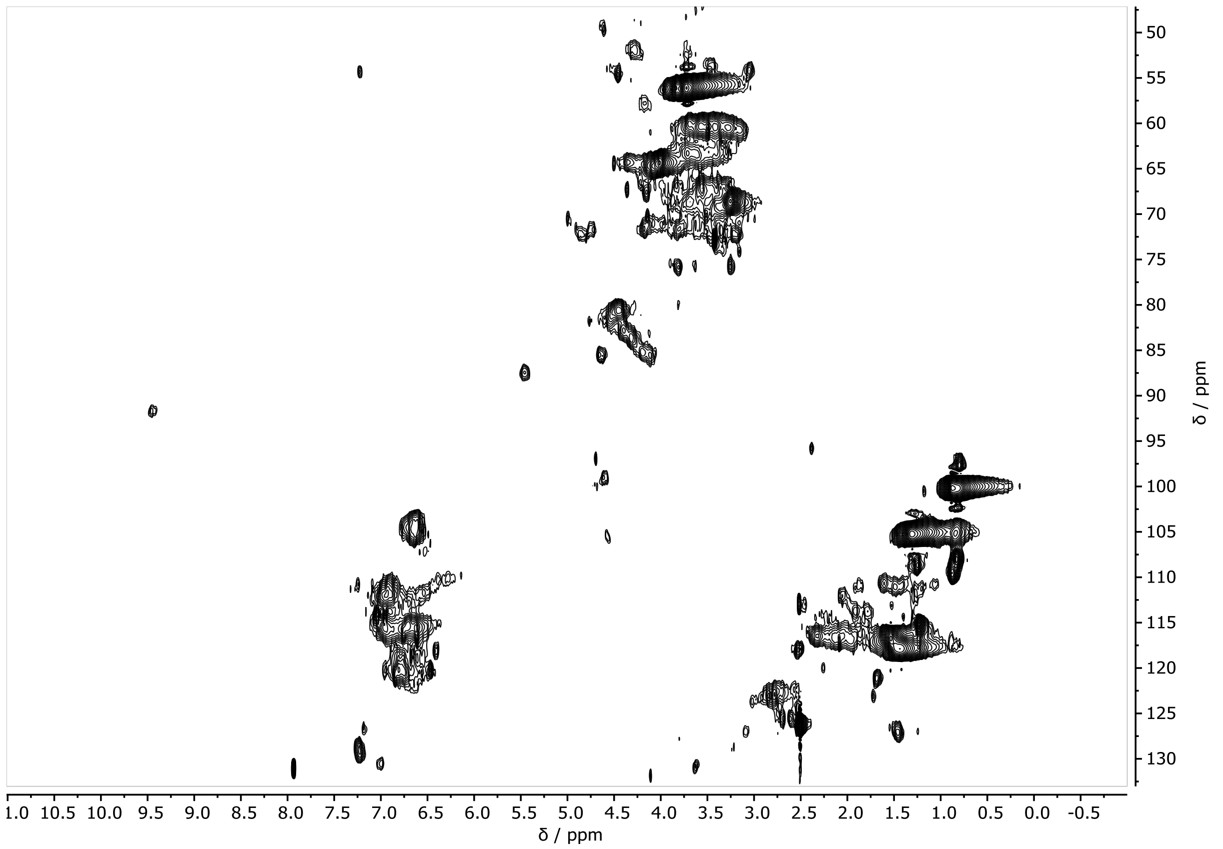

Figure S46: HSQC NMR (700 MHz, DMSO-d_6) of CPH lignin from literature[S3] butanosolv pretreatment after hexane/diethyl ether precipitation

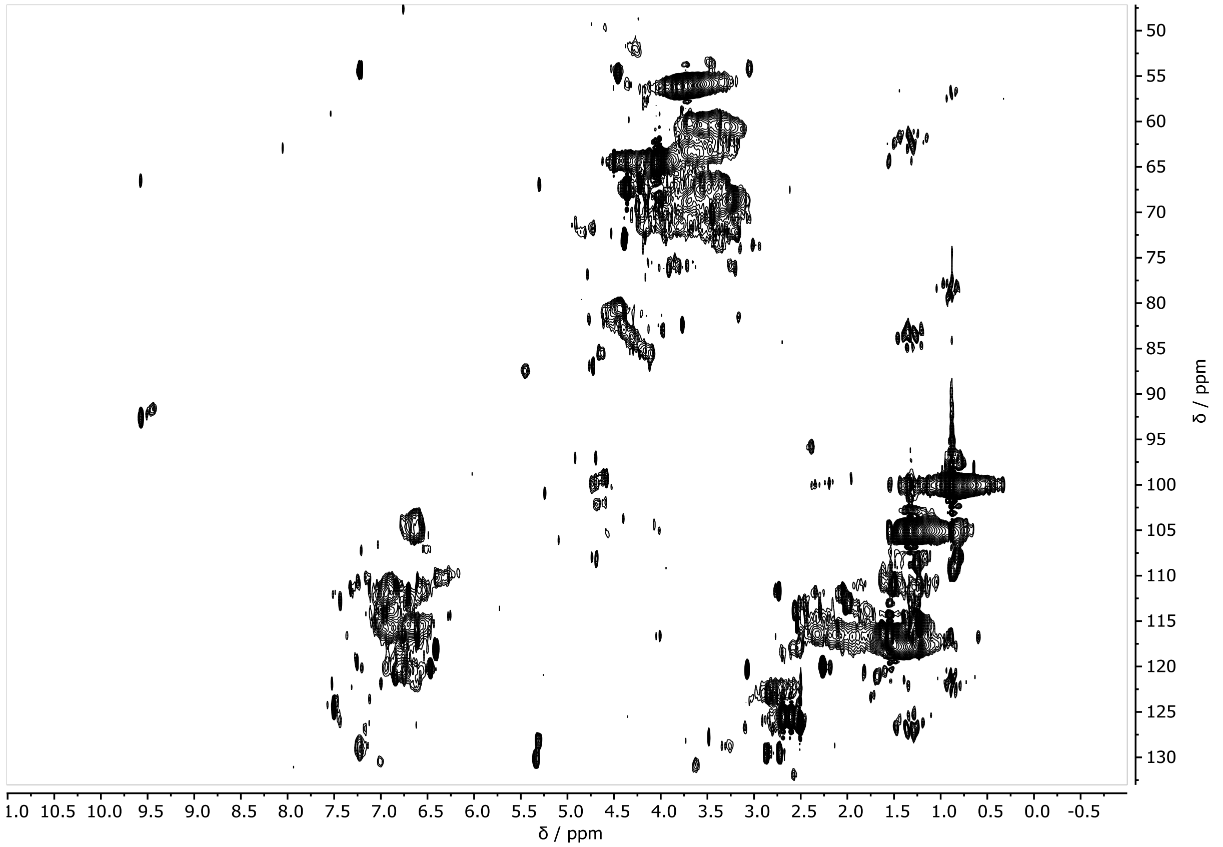

Figure S47: HSQC NMR (700 MHz, DMSO-d_6) of CPH lignin from literature[S3] butanosolv pretreatment before hexane/diethyl ether precipitation

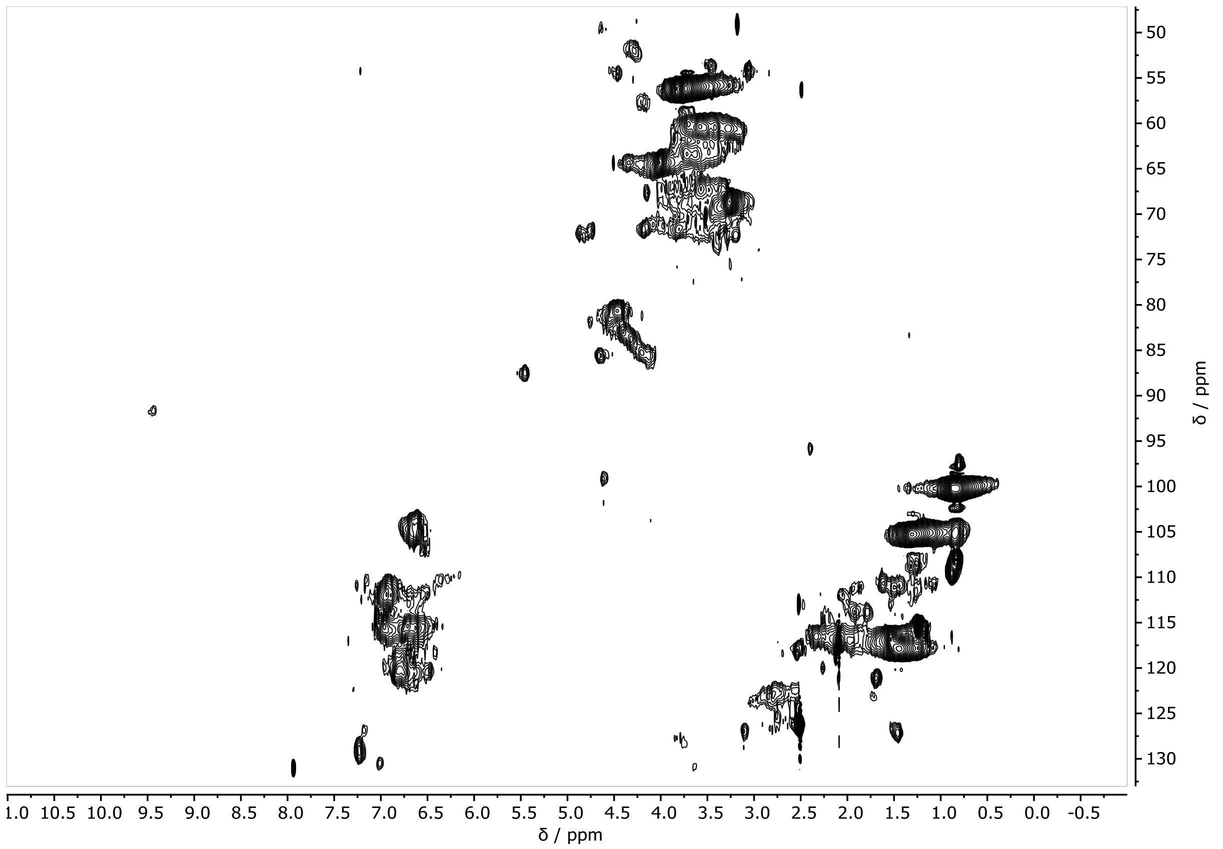

Figure S48: HSQC NMR (700 MHz, DMSO-d_6) of CPH lignin from literature[S3] butanosolv pretreatment after caustic soda treatment.

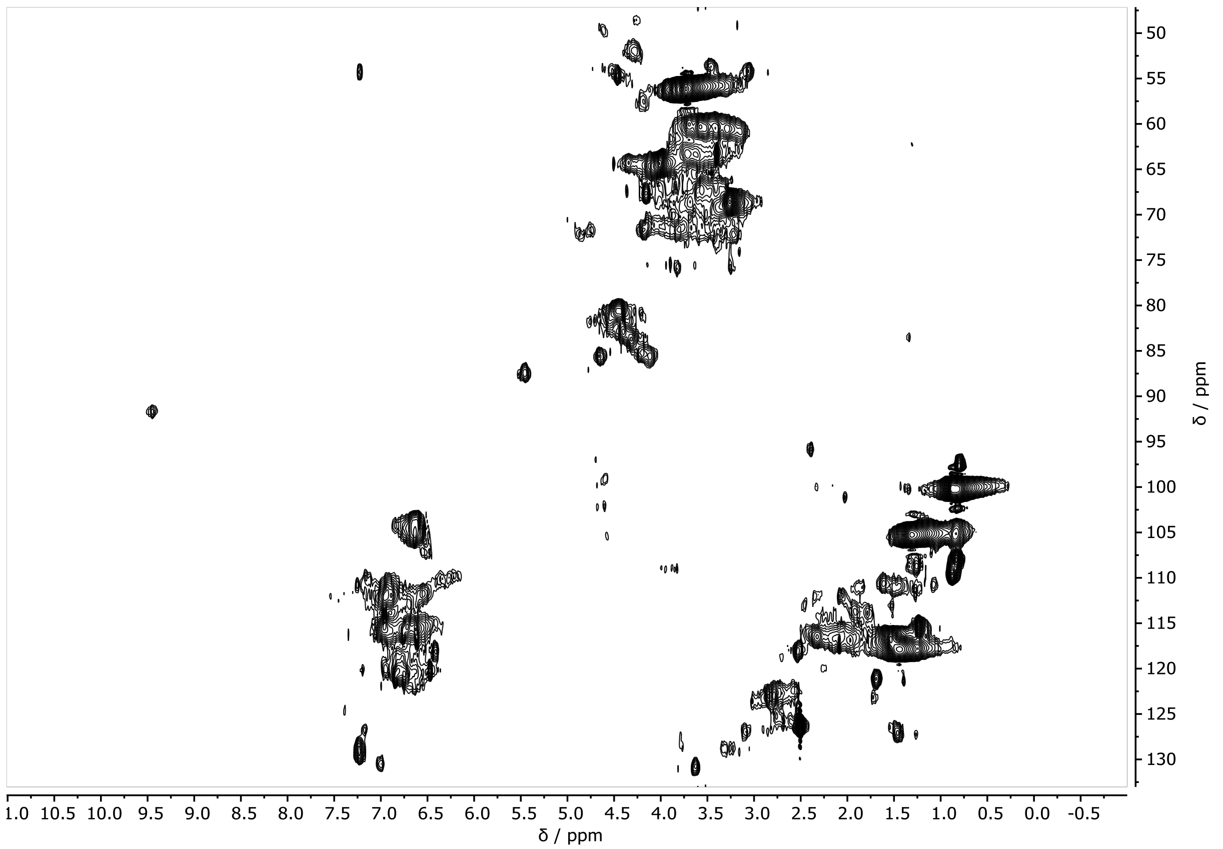

Figure S49: HSQC NMR (700 MHz, DMSO-d_6) of CPH lignin from ethanol pre-wash followed by nutanosolv pretreatment**.**

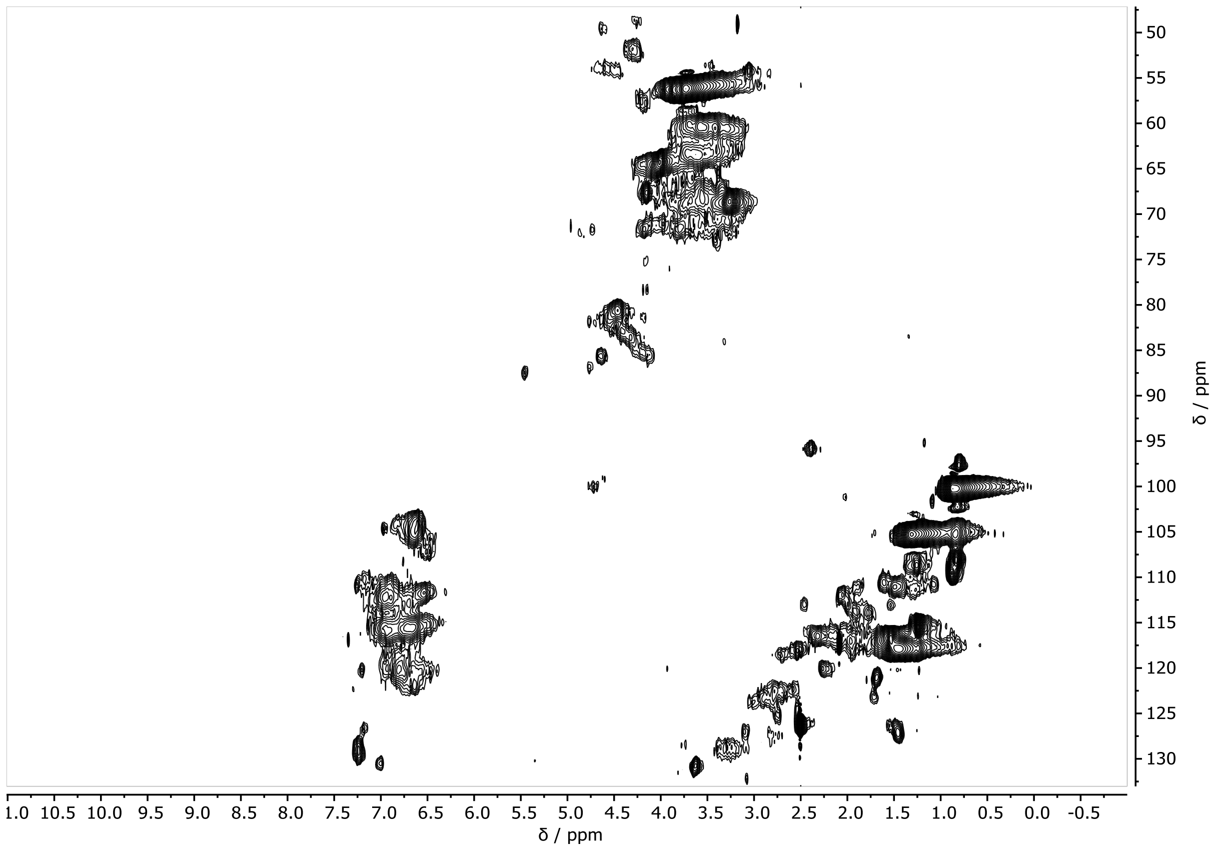

Figure S50: HSQC NMR (700 MHz, DMSO-d_6) of CPH lignin from hot water extraction followed by butanosolv pretreatment.

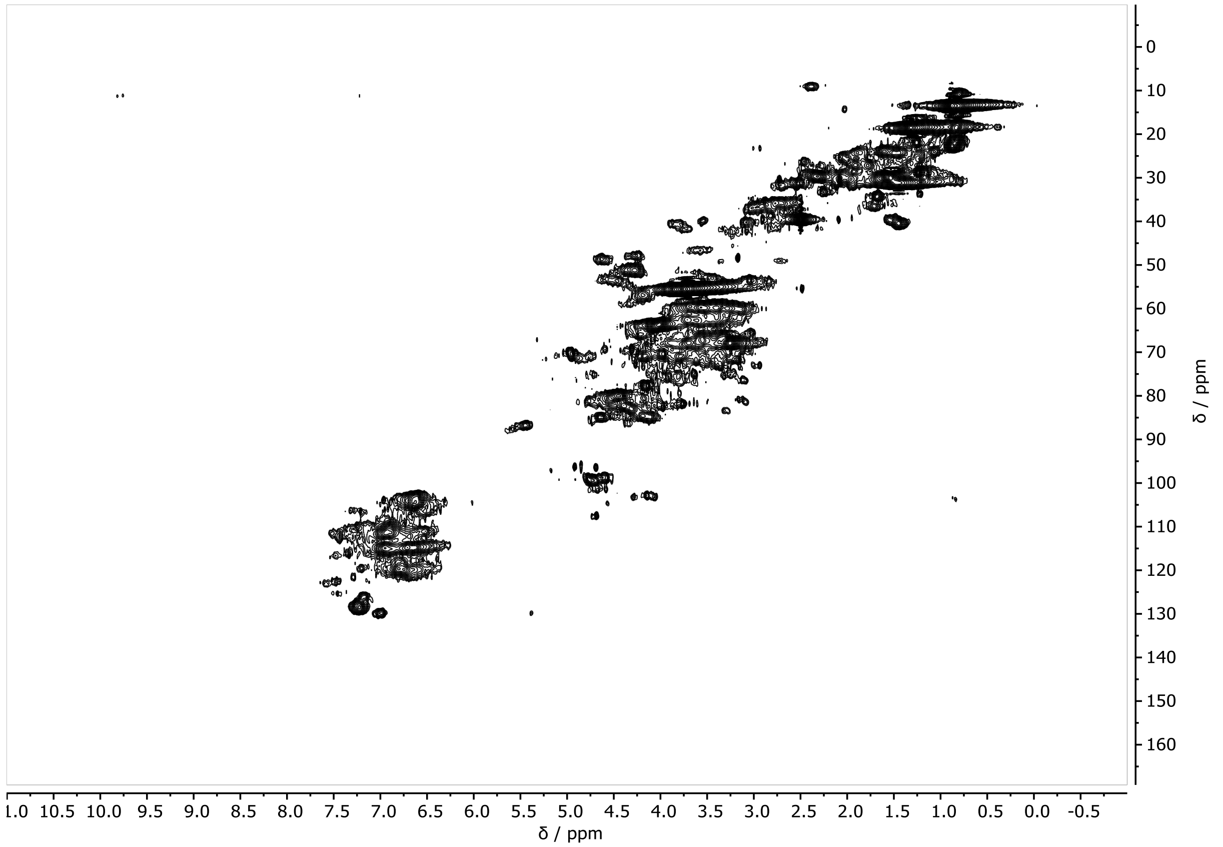

Figure S51: HSQC NMR (700 MHz, DMSO-d_6) of CPH lignin from optimised pretreatment.

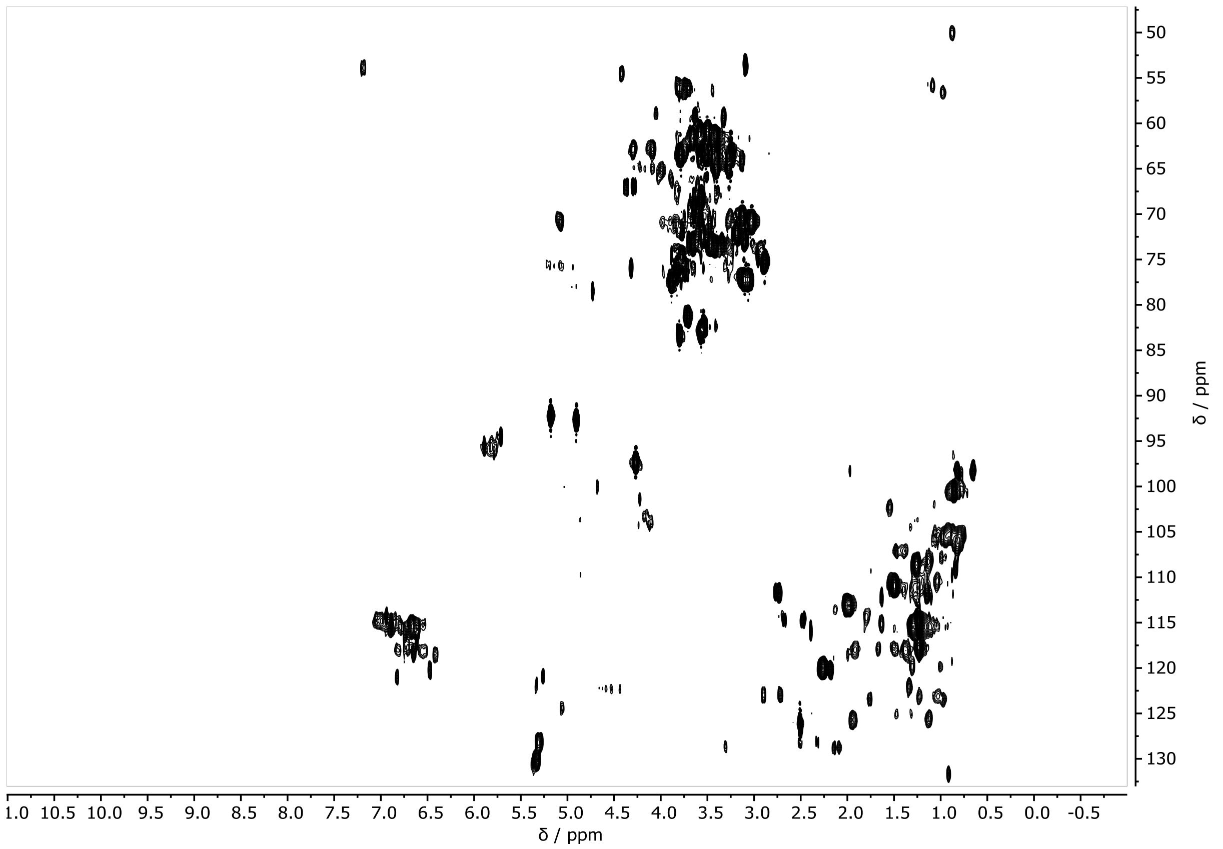

Figure S52: HSQC NMR (700 MHz, DMSO-d_6) of concentrated ethanol prewash from optimised butanosolv pretreatment.

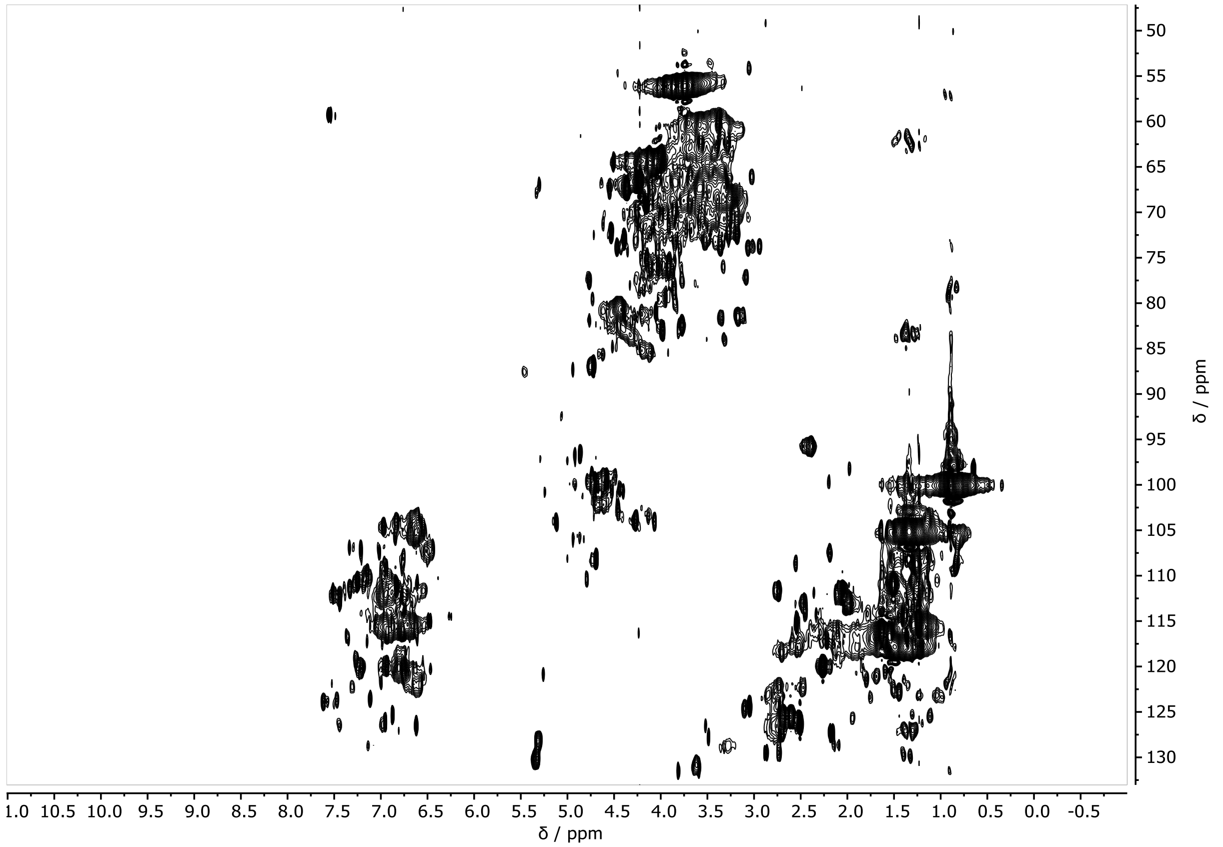

Figure S53: HSQC NMR (700 MHz, DMSO-d_6) of filtrate from final purification from optimised butanosolv pretreatment.

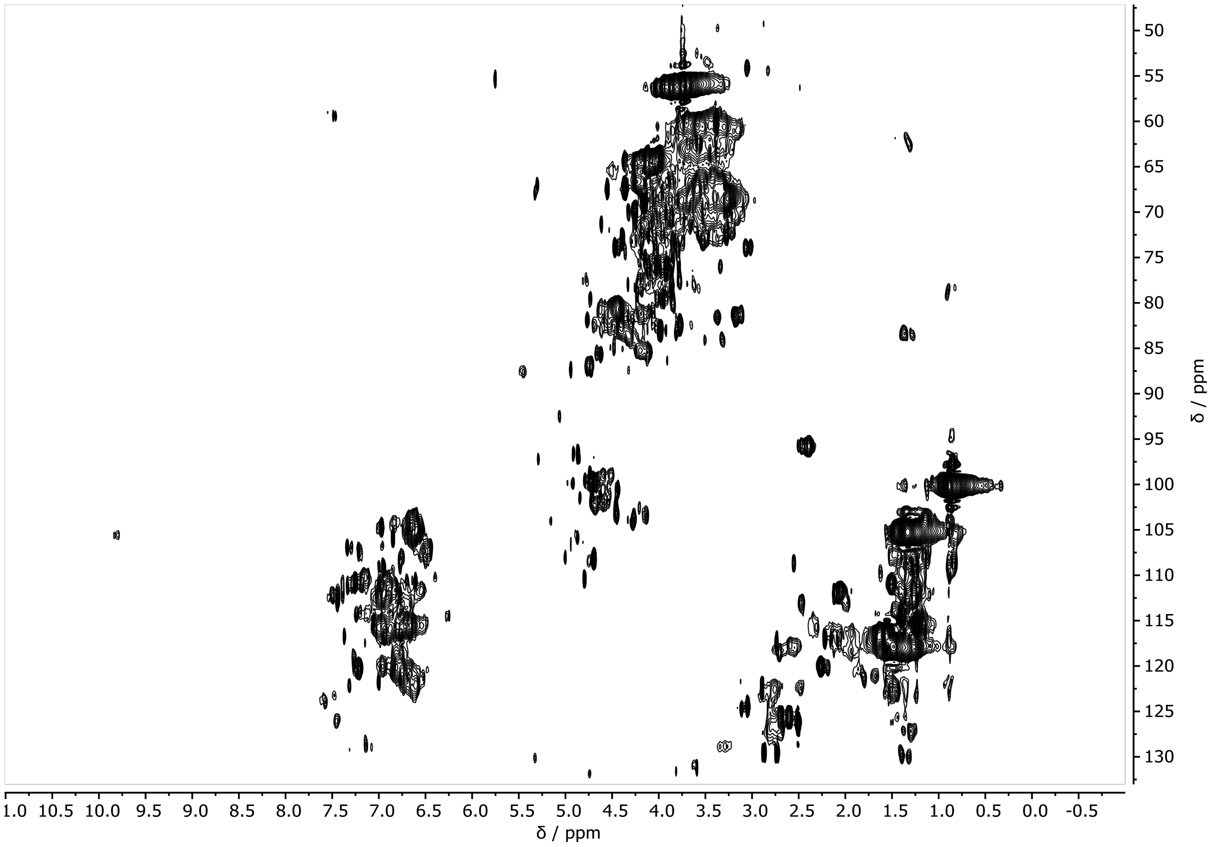

Figure S54: HSQC NMR (700 MHz, DMSO-d_6) of additional CPH lignin obtained from filtrate from final purification from optimised butanosolv pretreatment.

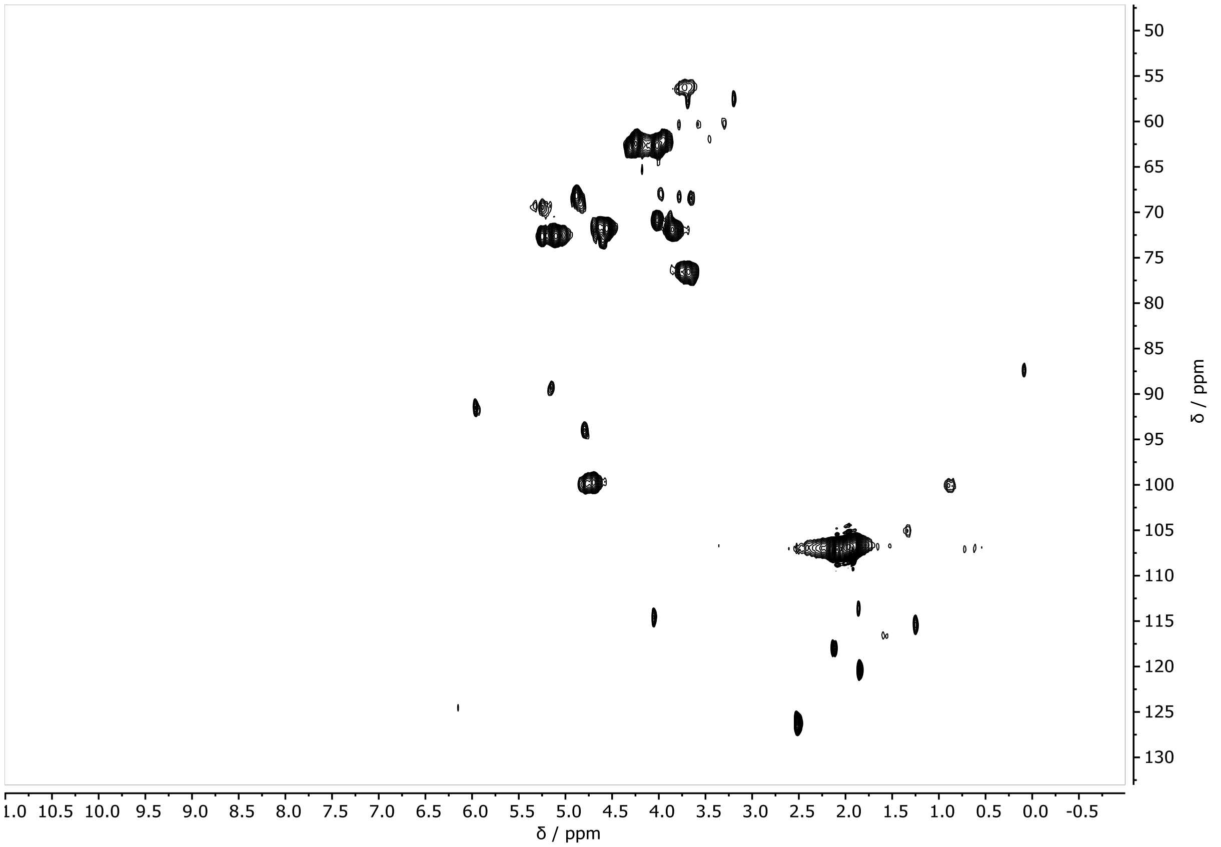

Figure S55: HSQC NMR (500 MHz, DMSO-d_6) of AcBr reaction of CPH cellulose pulp for optimised pretreatment.

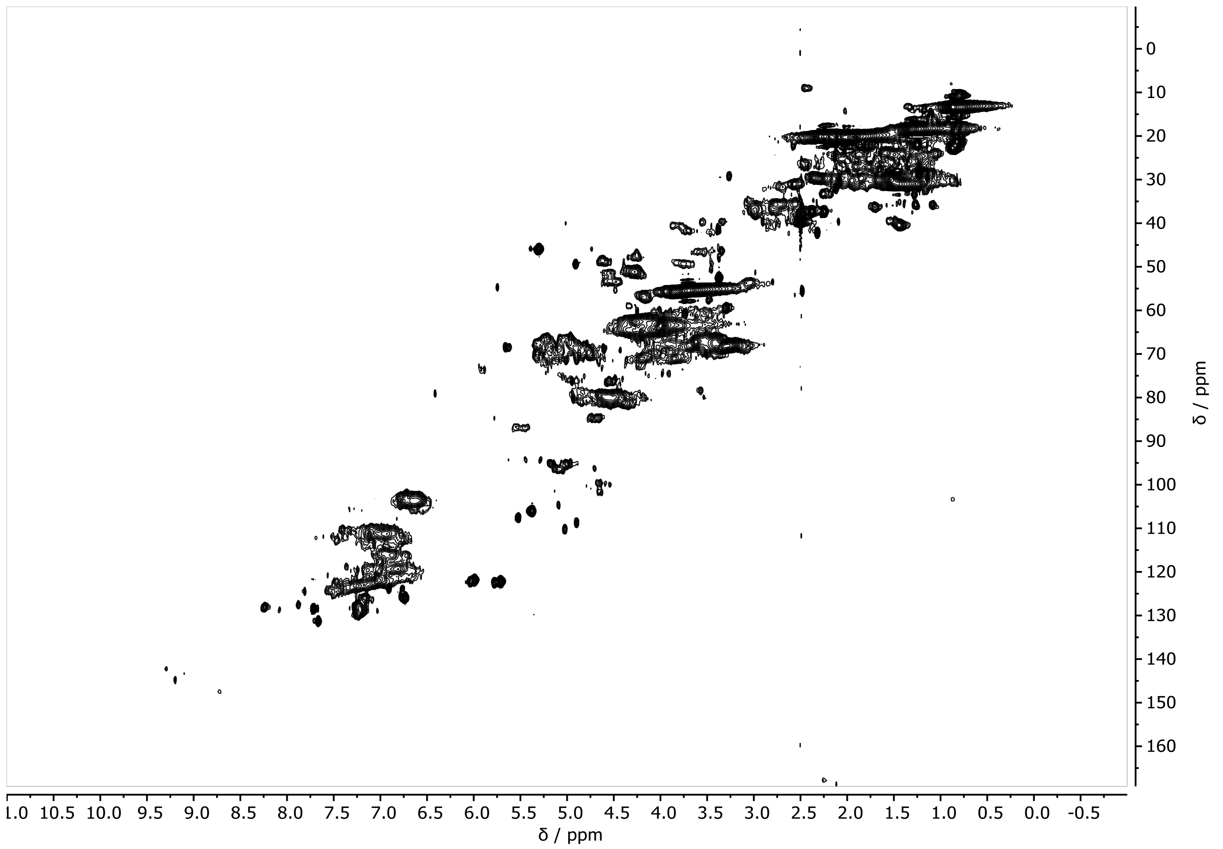

Figure S56: HSQC NMR (700 MHz, DMSO-d$_6$) of acetylated CPH lignin from optimised pretreatment

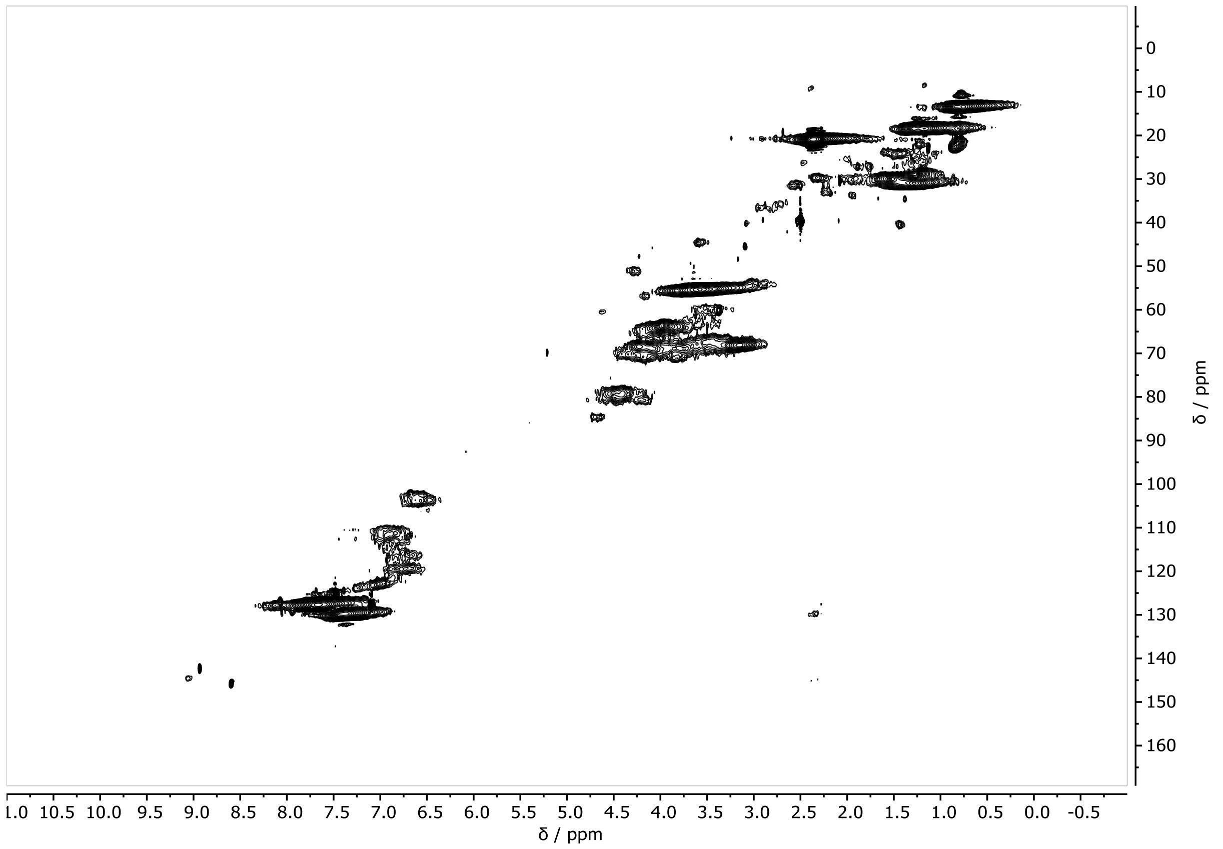

Figure S57: HSQC NMR (700 MHz, DMSO-d$_6$) of Lignin-Ts

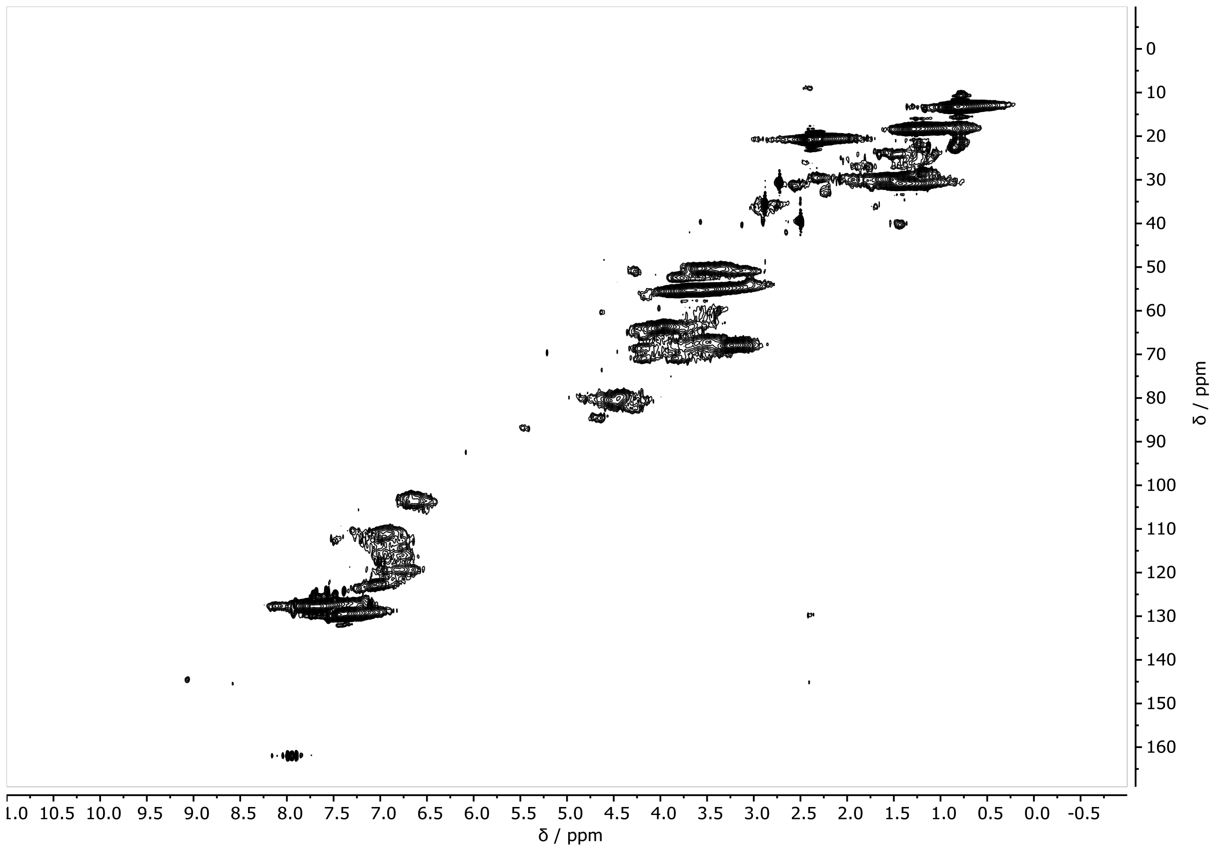

Figure S58: HSQC NMR (700 MHz, DMSO-d_6) of Lignin-N_3

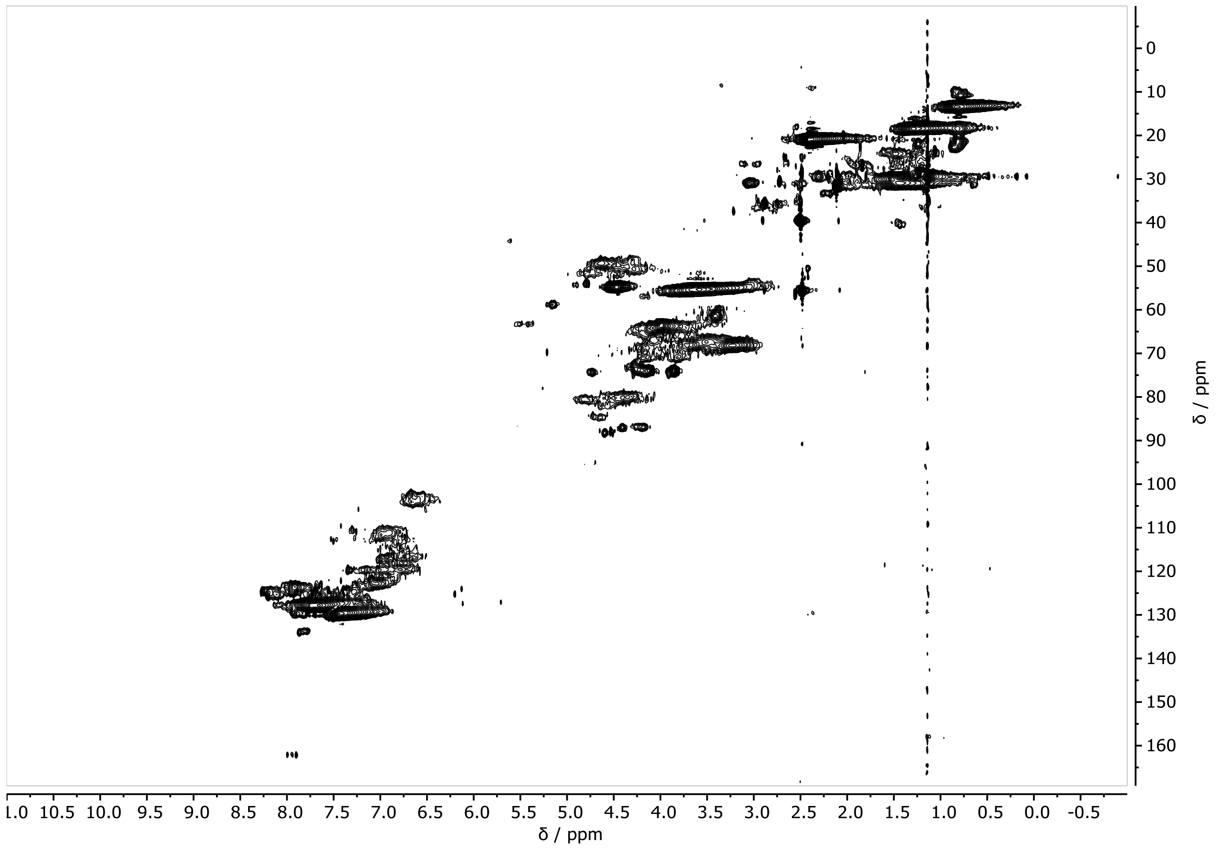

Figure S59: HSQC NMR (700 MHz, DMSO-d_6) of Lignin-DOPO

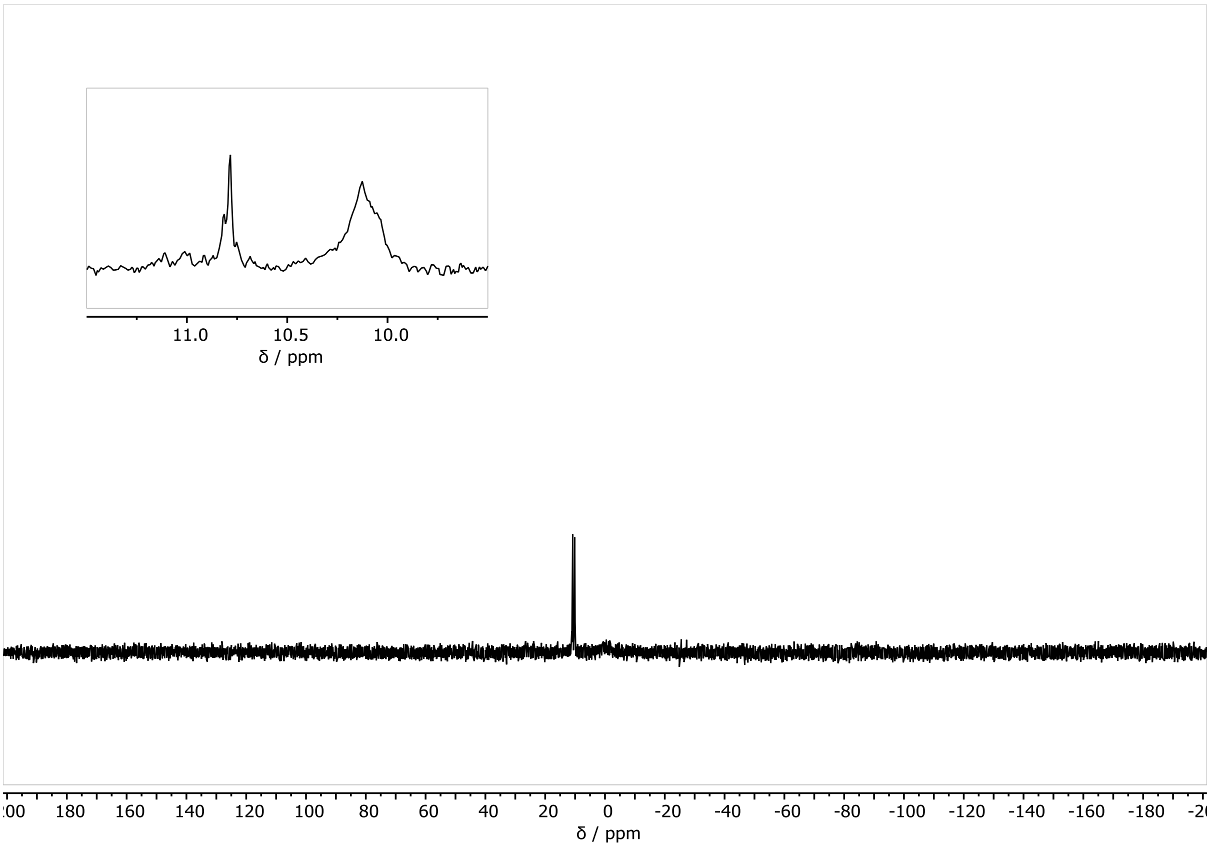

Figure S60: ^{31}P NMR (202 MHz, DMSO-d$_6$) of Lignin-DOPO

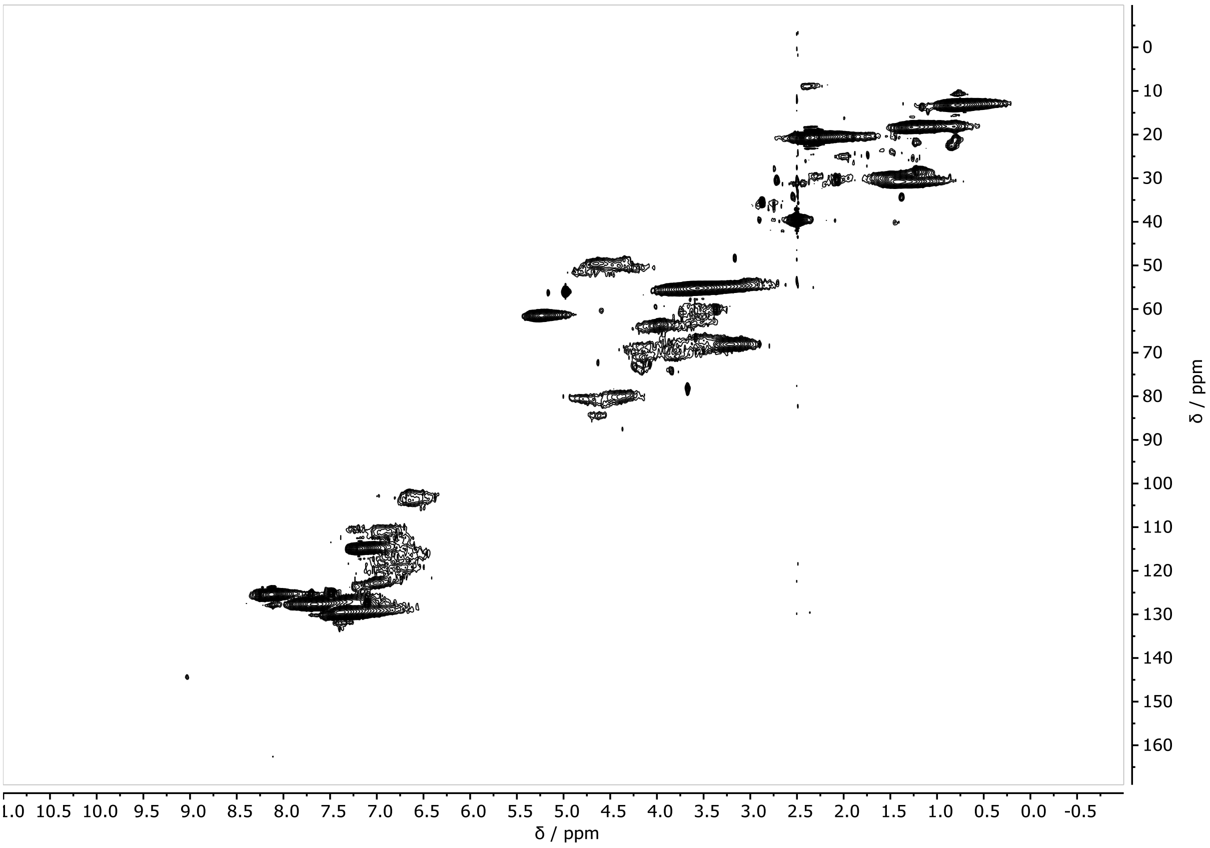

Figure S61: HSQC NMR (700 MHz, DMSO-d$_6$) of Lignin-N$_3$ clicked with 1-nitro-4-(prop-2-yn-1-yloxy)benzene

References

(S1) Xiang, Z.; Watson, J.; Tobimatsu, Y.; Runge, T. Film-Forming Polymers from Distillers' Grains: Structural and Material Properties. *Industrial Crops and Products* **2014**, *59*, 282–289. https://doi.org/10.1016/J.INDCROP.2014.05.023

(S2) Xiao, B.; Sun, X. F.; Sun, R. C. Chemical, Structural, and Thermal Characterizations of Alkali-Soluble Lignins and Hemicelluloses, and Cellulose from Maize Stems, Rye Straw, and Rice Straw. *Polymer Degradation and Stability* **2001**, *74* (2), 307–319. https://doi.org/10.1016/S0141-3910(01)00163-X

(S3) Lancefield, C. S.; Panovic, I.; Deuss, P. J.; Barta, K.; Westwood, N. J. Pre-Treatment of Lignocellulosic Feedstocks Using Biorenewable Alcohols: Towards Complete Biomass Valorisation. *Green Chemistry* **2017**, *19* (1), 202–214. https://doi.org/10.1039/C6GC02739C

(S4) Zijlstra, D. S.; de Korte, J.; de Vries, E. P. C.; Hameleers, L.; Wilbers, E.; Jurak, E.; Deuss, P. J. Highly Efficient Semi-Continuous Extraction and In-Line Purification of High β-O-4 Butanosolv Lignin. *Frontiers in Chemistry* **2021**, *9*, 329. https://doi.org/10.3389/FCHEM.2021.655983

(S5) Panovic, I.; Montgomery, J. R. D.; Lancefield, C. S.; Puri, D.; Lebl, T.; Westwood, N. J. Grafting of Technical Lignins through Regioselective Triazole Formation on β-O-4 Linkages. *ACS Sustain. Chem. Eng.* **2017**, *5* (11), 10640–10648. https://doi.org/10.1021/acssuschemeng.7b02575

(S6) Kim, H.; Padmakshan, D.; Li, Y.; Rencoret, J.; Hatfield, R. D.; Ralph, J. Characterization and Elimination of Undesirable Protein Residues in Plant Cell Wall Materials for Enhancing Lignin Analysis by Solution-State Nuclear Magnetic Resonance Spectroscopy. *Biomacromolecules* **2017**, *18* (12), 4184–4195. https://doi.org/10.1021/acs.biomac.7b01223

(S7) Shen, Q.; Mu, D.; Yu, L. W.; Chen, L. A Simplified Approach for Evaluation of the Polarity Parameters for Polymer Using the K Coefficient of the Mark-Houwink-Sakurada Equation. *J. Colloid Interf. Sci.* **2004**, *275* (1), 30–34. https://doi.org/10.1016/j.jcis.2004.01.041

(S8) Montgomery, J. R. D.; Lancefield, C. S.; Miles-Barrett, D. M.; Ackermann, K.; Bode, B. E.; Westwood, N. J.; Lebl, T. Fractionation and DOSY NMR as Analytical Tools: From Model Polymers to a Technical Lignin. *ACS Omega* **2017**, *2* (11), 8466–8474. https://doi.org/10.1021/acsomega.7b01287

(S9) Ralph, J.; Hatfield, R. D.; Quideau, S.; Helm, R. F.; Grabber, J. H.; Jung, H. J. G. Pathway of P-Coumaric Acid Incorporation into Maize Lignin As Revealed by NMR. *J Am Chem Soc* **1994**, *116* (21), 9448–9456. https://doi.org/10.1021/JA00100A006

(S10) Wu, Y.; Huang, Z.; Lv, K.; Rao, Y.; Chen, Z.; Zhang, J.; Long, J. Producing Methyl P-Coumarate from Herbaceous Lignin via a "Clip-Off" Strategy. *Journal of Agricultural and Food Chemistry* **2022**. https://doi.org/10.1021/ACS.JAFC.1C08353

(S11) Hilgers, R.; Vincken, J. P.; Kabel, M. A. Facile Enzymatic Cγ-Acylation of Lignin Model Compounds. *Catalysis Communications* **2020**, *136*, 105919. https://doi.org/10.1016/J.CATCOM.2019.105919.

(S12) Zhu, X.; Liu, B.; Zheng, S.; Gao, Y. Quantitative and Structure Analysis of Pectin in Tobacco by 13 C CP/MAS NMR Spectroscopy. *Anal. Methods* **2014**, *6*, 6407–6413. https://doi.org/10.1039/c4ay01156b

(S13) Massiot, D.; Fayon, F.; Capron, M.; King, I.; le Calvé, S.; Alonso, B.; Durand, J. O.; Bujoli, B.; Gan, Z.; Hoatson, G. Modelling One- and Two-Dimensional Solid-State NMR Spectra. *Magnetic Resonance in Chemistry* **2002**, *40* (1), 70–76. https://doi.org/10.1002/MRC.984

(S14) Rahimi, A.; Azarpira, A.; Kim, H.; Ralph, J.; Stahl, S. S. Chemoselective Metal-Free Aerobic Alcohol Oxidation in Lignin. *J Am Chem Soc* **2013**, *135* (17), 6415–6418. https://doi.org/10.1021/JA401793N
